# Darkness and body size shaped end-Cretaceous marine extinction patterns

**DOI:** 10.1038/s41586-026-10541-4

**Published:** 2026-05-27

**Authors:** Rui Ying, Fanny M. Monteiro, James D. Witts, Daniela N. Schmidt

**Affiliations:** 1 School of Earth Sciences, University of Bristol, Bristol, UK; 2 School of Environmental Sciences, University of East Anglia, Norwich, UK; 3 School of Geographical Sciences, University of Bristol, Bristol, UK; 4 iC3: Centre for Ice, Cryosphere, Carbon and Climate, Department of Geosciences, UiT The Arctic University of Norway, Tromsø, Norway; 5 Natural History Museum, London, UK

**Keywords:** Palaeoecology, Ecological modelling, Palaeoceanography, Biodiversity, Palaeontology

## Abstract

The Chicxulub asteroid impact at the Cretaceous–Paleogene (K–Pg) boundary (66 Ma) is thought to have caused the extinction of around 75% of species in the fossil record by triggering catastrophic environmental changes^1^. However, despite decades of research, the mechanisms linking the environmental changes to the selective extinction patterns observed in the marine fossil record remain unresolved. Here we use a global trait-based ecosystem model^2,3^ to establish this causality for the marine plankton community beyond the fossilized groups. Our model simulates diversity dynamics during the initial 100 years after the K–Pg boundary and represents explicitly extinction based on biomass thresholds that scales with body size. Under K–Pg climatic forcings, the model reproduces successfully key observed extinction patterns, including the high vulnerability of planktic foraminifera and other zooplankton, the survival of small mixotrophs^4^ and phytoplankton^5,6^, and potential for reduced diversity loss in high-latitude settings^7^. Our analysis suggests that impact-driven darkness and body-size-dependent extinction thresholds drove most of the observed extinction patterns. These results suggest that plankton ecologies enhance survival through differences in energy demand and acquisition. Our study bridges the gap between fossil evidence of extinction patterns and the K–Pg impact winter hypothesis, highlighting the value of trait-based models for understanding past biodiversity crises.

## Abstract (web summary)

Trait-based ecosystem modelling shows that impact-driven darkness and body-size-dependent extinction thresholds, rather than carbon-dioxide increase or acidification, drove most of the observed end-Cretaceous marine extinction patterns.

## Main

The Cretaceous–Paleogene (K–Pg) boundary (66 Ma) is marked by a mass extinction event^1^ that altered Earth’s terrestrial and marine biosphere profoundly. Both the emplacement of the Deccan Trap^8^ and the Chicxulub asteroid impact on the Yucatán carbonate platform were recognized as the potential drivers of this mass extinction. However, overwhelming evidence supports the latter triggering the marine extinction through abrupt environmental changes^9,10^. The bolide impact and associated wildfires released silicate dust, soot and sulfur aerosols into the atmosphere^9^, blocking solar radiation and causing the reduction of light and global cooling^11^. Simultaneously, global wildfires and the vaporization of carbonate-rich target rock increased CO_2_ concentrations by approximately 700 ppm across K–Pg^12^.

Despite advances in reconstructing the sequence of events across the K–Pg boundary, it is still unclear how environmental changes caused observed extinction patterns^13^. In the ocean, nearly all nannoplankton, planktic foraminifera, all rudist bivalve and ammonoid cephalopod molluscs went extinct^14–16^. By contrast, dinoflagellates, diatoms, radiolarians and benthic foraminifera were less affected^17,18^. Notably, high-latitude nannoplankton^7^, particularly in the Southern Ocean, have exhibited lower extinction rates than their low-latitude counterparts, and a similar pattern might exist for planktic foraminifera^19^. However, Northern Hemisphere high-latitude data remain limited^7^ and such a latitude-dependent extinction was not found in molluscs^20^. Furthermore, surviving nannoplankton and foraminifera were small and opportunistic^4,21^, with similar size reductions found in other marine organisms^22^.

Several hypotheses have been proposed to explain these ecological and geographical selective extinction patterns across the K–Pg boundary. Alvarez and colleagues^23^ suggested a dramatic loss of marine primary production due to reduced solar radiation, leading to a cascading trophic collapse. However, subsequent observations show that the reduction in productivity across the K–Pg was relatively modest^24^ and spatially heterogeneous^25^. The basin-dependent productivity change across the K–Pg also does not match the latitude-dependent extinction^25^. Instead, ocean acidification might have contributed to the higher extinction rate of calcifying organisms compared with silicifying organisms^26^. However, ocean acidification at the K–Pg was limited^12^ compared with other geological events^27^, similar to the Paleocene-Eocene Thermal Maximum where comparable acidification levels did not result in a global extinction of calcifiers^28^. These studies show that a mechanistic understanding is still lacking to reconcile the ecological and geographical selectivity observed in the fossil record of the K–Pg extinction.

Mechanistic ecosystem and biogeochemical models within Earth system models provide a powerful tool for linking K–Pg environmental changes to marine plankton dynamics. However, existing marine biogeochemical models do not simulate mass extinction explicitly, as these models focus typically on biogeochemical cycling and allow plankton populations to recover immediately under favourable conditions, even from extremely low biomass levels, which could obscure true extinction^4,29^. Furthermore, extinction thresholds for different marine groups remain poorly constrained^30^, hindering the reproduction and consequent investigation of observed patterns of extinction selectivity.

Body size is recognized widely as a master trait that shapes organism biology and strongly influences extinction thresholds of marine organisms in the Phanerozoic^31^. Larger organisms tend to be more vulnerable during mass extinctions due to their higher energy demands, lower population density and slower mass-specific metabolic rates^32,33^. This suggests that body size-dependent extinction thresholds could provide a mechanistic link between climate change, plankton ecology and extinction risk, yet these are not explored for the end-Cretaceous crisis.

**Fig. 1:**
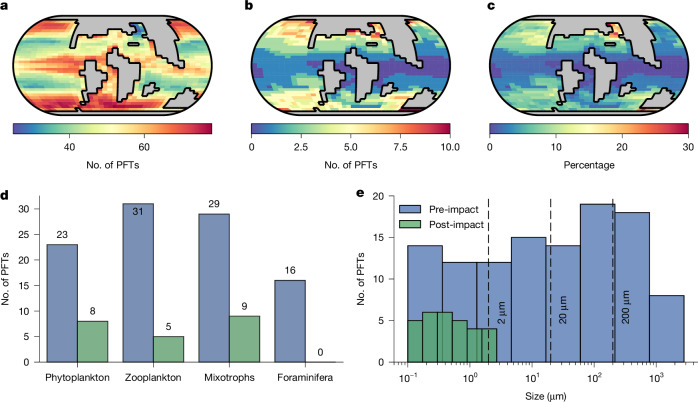
Modelled plankton extinction selectivity in response to K–Pg climate changes. **a**,**b**, PFT richness before (**a**) and after (**b**) the K–Pg impact. **c**, Percentage of plankton survivors. **d**, Diversity of various PFTs before and after the K–Pg impact; this excludes the Arctic Ocean, which the model does not represent due to limited grid resolution. **e**, Size distribution across all existing plankton types before and after impact. Dashed vertical lines represent 2, 20 and 200 µm. Note that the colour scale varies between **a**, **b** and **c** to highlight geographical patterns.

Here we use a size-based mechanistic ecosystem model (EcoGENIE) to investigate the causes of observed extinction patterns in plankton communities across the K–Pg. EcoGENIE resolves size-dependent plankton ecophysiological processes explicitly within a three-dimensional ocean circulation and biogeochemistry framework (Supplementary Fig. 1). To overcome limitations of previous modelling approaches, we implement an extinction mechanism in EcoGENIE based on a size-dependent biomass threshold, defined as the biomass of a single individual of a given size. Larger plankton thus have higher biomass threshold and higher extinction vulnerability. We initialize the model with a diverse plankton community (32 phytoplankton, 32 generic zooplankton, 32 mixotrophs and 16 foraminifera functional types across size classes; Supplementary Table 1)^34,35^. This model allows environmental conditions to select which Late Cretaceous plankton functional types to go extinct in response to K–Pg environmental changes (Fig. 1). Such an approach enables us to examine extinction selectivity across the entire plankton community, including those without mineralized shells and hence absent from the fossil record. Furthermore, the explicit inclusion of planktic foraminifera within the zooplankton group offers an opportunity to validate model results directly against fossil data.

## Late Cretaceous climate and diversity

**Extended Data Fig. 1:**
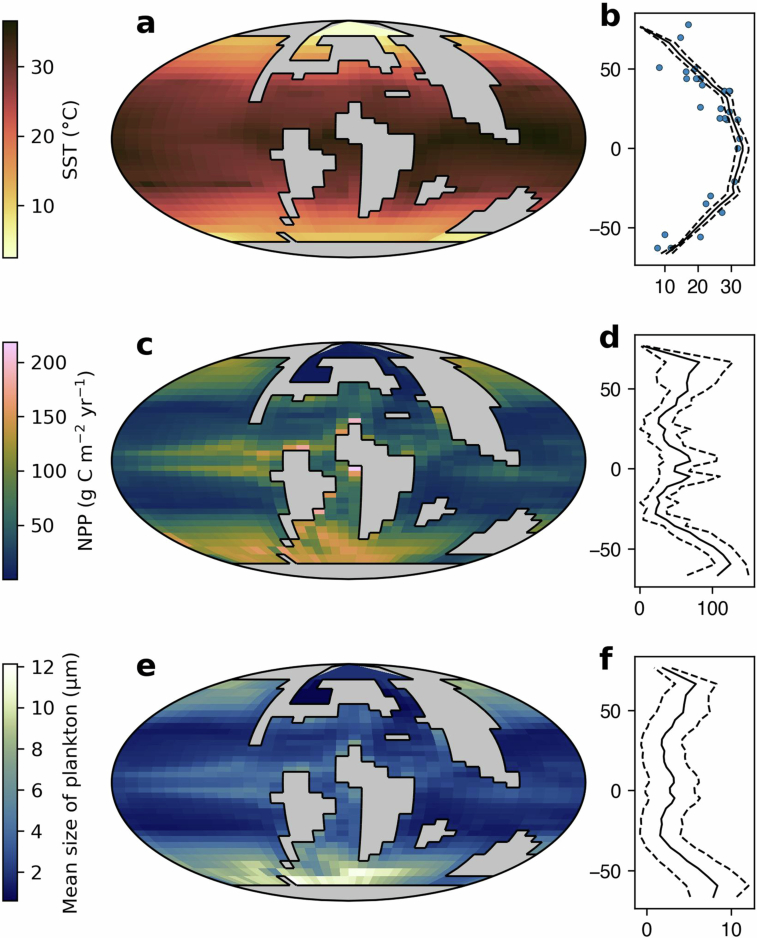
Spun-up model ocean states for the Late Cretaceous (Maastrichtian Stage). (a) Annual mean sea surface temperature (°C); (c) Annual mean net primary production (g C m^−2^ yr^−1^) and (e) plankton community biomass-weighted mean body size. The right panels (b,d,f) show the zonal mean and standard deviation equivalents. Blue dots in (b) represent Late Cretaceous proxy sea-surface temperature data from Zhang et al.^36^.

We applied the cGENIE model to a Late Cretaceous climate state (834 ppm CO_2_) and paleogeographic configuration (Supplementary Fig. 2 and Methods). The spin-up simulation produced a global mean sea surface temperature of around 26.4 °C, closely matching the latest data compilation based on TEX_86_ and δ^18^O proxies^36^ (Extended Data Fig. 1). The modelled deep-water temperature (deeper than 2,000 m) and surface pH are 9.3 °C and 7.7, respectively, both in line with Late Cretaceous oxygen and boron isotope estimates (9 °C and 7.7–7.8 pH units)^12,37^. In contrast to other models^29,38^, cGENIE succeeds in simulating a reduced equator-to-pole temperature gradient, in agreement with proxy data. Hence, it provides a realistic climatic environment to test extinction selectivity.

In the spin-up experiment, 99 plankton functional types survived, representing around 88% of the initial community. The emergent plankton diversity (defined as the coexisting number of plankton functional types) was highest in equatorial and coastal upwelling regions and at high latitudes (Fig. 1a). This spatial pattern aligns with the theory that the number of supported plankton size classes increases with nutrient supply rate^39^, which captures modern plankton observations successfully^40^. Therefore, although fossil data are spatially incomplete and biased towards groups with tests or shells, our model provides a plausible estimate of global plankton functional diversity in the Late Cretaceous.

## Modelling the K–Pg extinction

**Extended Data Fig. 2:**
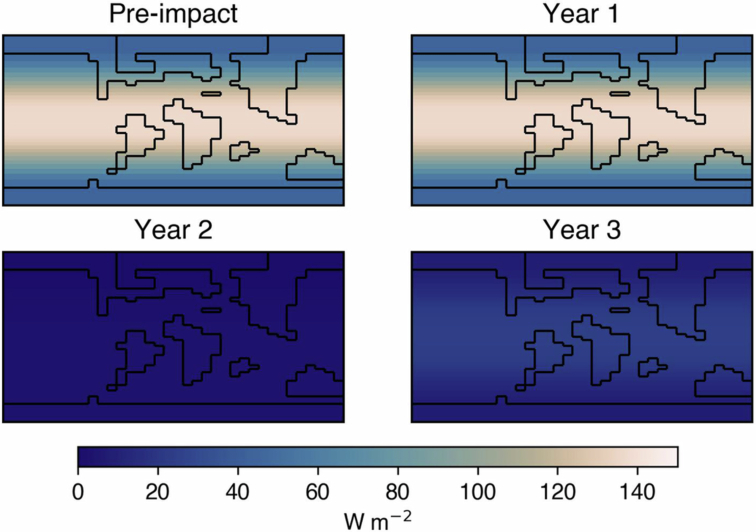
Temporal evolution of Photosynthetically Active Radiation (PAR; W m^−2^) forcing following the first 3 years after the K-Pg asteroid impact. The PARs for the first three years post-impact are roughly 0%, 20%, and 80% of the pre-impact value, respectively. Since the fourth year, the PAR has fully recovered to the pre-impact value.

**Extended Data Fig. 3:**
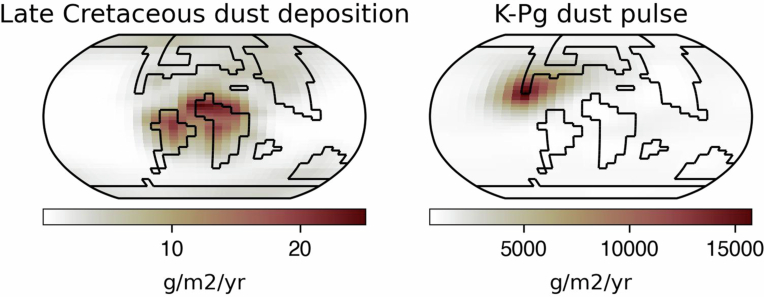
Dust deposition before and during the K-Pg impact. Late Cretaceous dust deposition distribution (left) and impact-driven dust pulse (right). Note the different colour scales between the two panels.

We then perturbed the Late Cretaceous baseline simulation for 100 years following the impact, using a 7.30-h timestep for ecosystem dynamics. Three K–Pg climatic forcings derived from previous studies^11,12^ were applied to ensure consistency (Supplementary Information): (1) a CO_2_ pulse of 700 ppm, based on boron isotope data from foraminifers^12^; (2) a reduction in solar radiation (Extended Data Fig. 2); and (3) an ejecta-derived nutrient (iron and phosphorus) flux (Extended Data Fig. 3). The CO_2_ perturbation affects both climate and seawater carbonate chemistry (for example, pH); however, we note that potential acidification impacts on plankton are missing as the model does not incorporate directly the effect of pH on plankton physiology and biomineralization (Methods). The solar radiation forcing follows estimates from Senel and colleagues^11^, which combined sedimentological evidence with a climate model capturing the effects of sulfur, soot and silicate dust. The projectile-derived nutrient flux and distribution were re-gridded from a previous modelling study^29^, with peak deposition concentrated near the Chicxulub impact site.

**Fig. 2:**
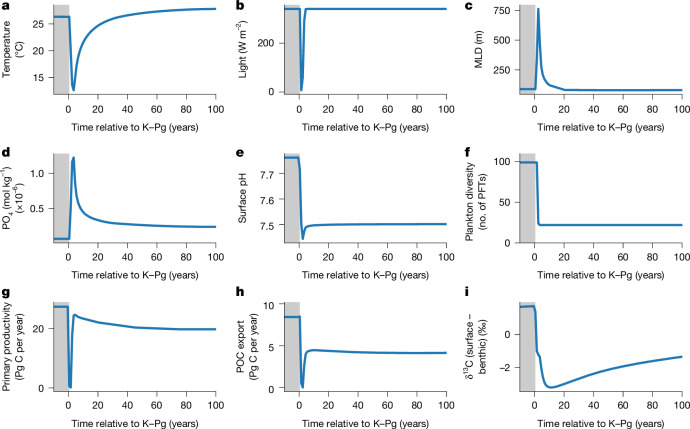
Ocean environmental and plankton community responses to abrupt K–Pg climate change within a century (100 years) of the Chicxulub impact. **a**, Global mean surface ocean temperature. **b**, Global mean insolation. **c**, Global mean MLD. **d**, Ice-free global mean phosphate concentration. **e**, Global mean ocean surface pH. **f**, Global coexisting PFTs. **g**, Total marine annual primary productivity. **h**, Annual particular carbon export (at 80.8 m). POC, particulate organic carbon. **i**, δ^13^C difference between surface (80.8 m) and benthic (deeper than 2,000 m) layers. Shaded areas, pre-impact Cretaceous.

**Extended Data Fig. 4:**
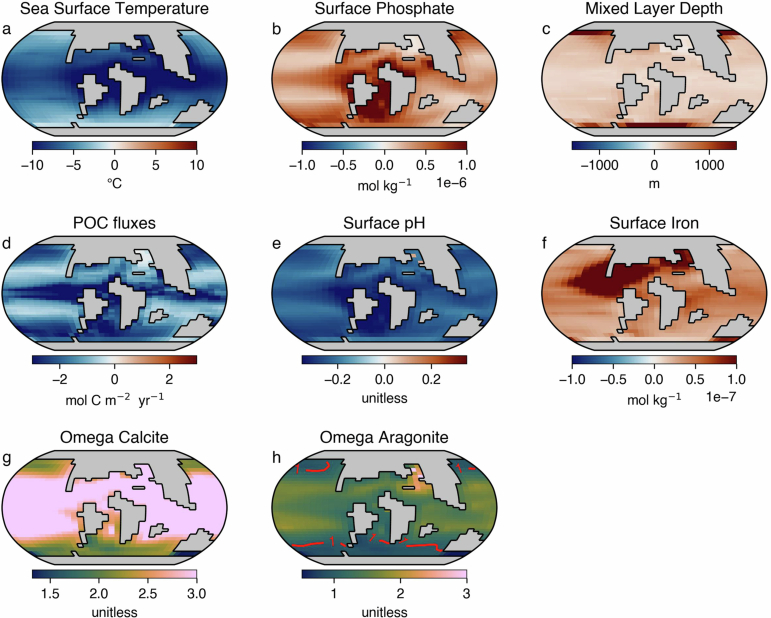
Changes in sea surface environmental conditions following the rapid K-Pg climate perturbation. All subplots show anomalies of the first two years post-impact compared to the pre-impact baseline. (a) Sea surface temperature, (b) surface PO_4_ concentration (mol kg^−1^), (c) mixed layer depth (m), (d) particulate organic carbon flux at 80.8 m depth (mol C m^−2^ yr^−1^), (e) surface ocean pH, (f) surface ocean dissolved iron concentration (mol kg^−1^). The saturation state of calcite and aragonite (omega) (g, h) are absolute values of the two-year average after the impact. Red contour lines indicate a saturation state of 1.

The transient experiment shows that global annual mean sea surface temperature dropped from 26.4 °C to a minimum of 12.3 °C within the first 3 years post-impact (Fig. 2 and Extended Data Fig. 4). Recovery to pre-impact temperatures took around 30 years, resulting in a prolonged global impact winter, despite an intense CO_2_ release (1,750 Pg C; Fig. 2a). This abrupt cooling nearly eliminated the vertical seawater density gradient (Supplementary Fig. 3), leading to the global mean ocean mixed layer depth (MLD) deepening dramatically to 750 m after 2 years (Fig. 2c). Such a loss of a stratified ocean would have impacted the vertical niche separation of plankton dramatically. The deepest mixed layer occurs at approximately 60° N/S, similar to results from a more complex ocean model^41^ (Supplementary Fig. 4). This increased vertical mixing, combined with enhanced dust-derived nutrient fluxes, led to a substantial rise in surface nutrient availability (Fig. 2d), with modelled PO_4_ concentrations increasing by 17 times within the first 2 years following the impact (from 0.07 to 1.2 μmol P kg^−1^).

**Extended Data Fig. 5:**
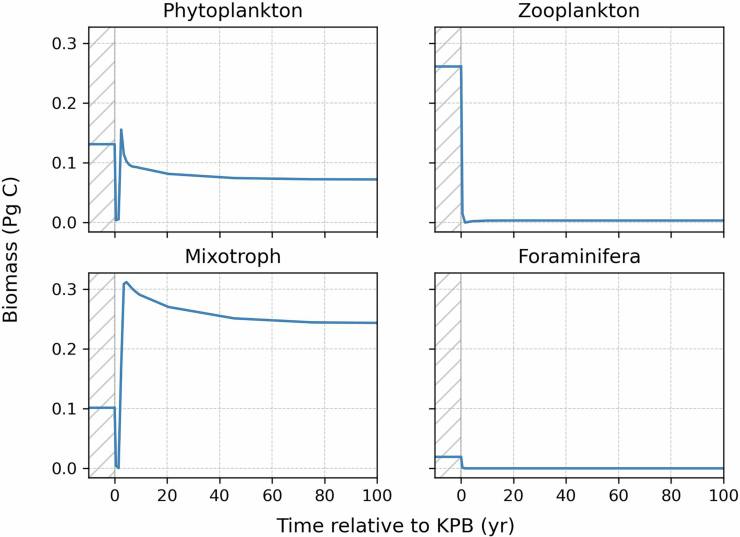
Evolution of plankton group-specific biomass in the K-Pg transient simulation. Biomass is integrated across all size classes within each plankton group. Following the extinction event, mixotrophs outcompete phytoplankton and become dominant, while generic zooplankton and foraminifera experience a near-complete biomass collapse.

**Extended Data Fig. 6:**
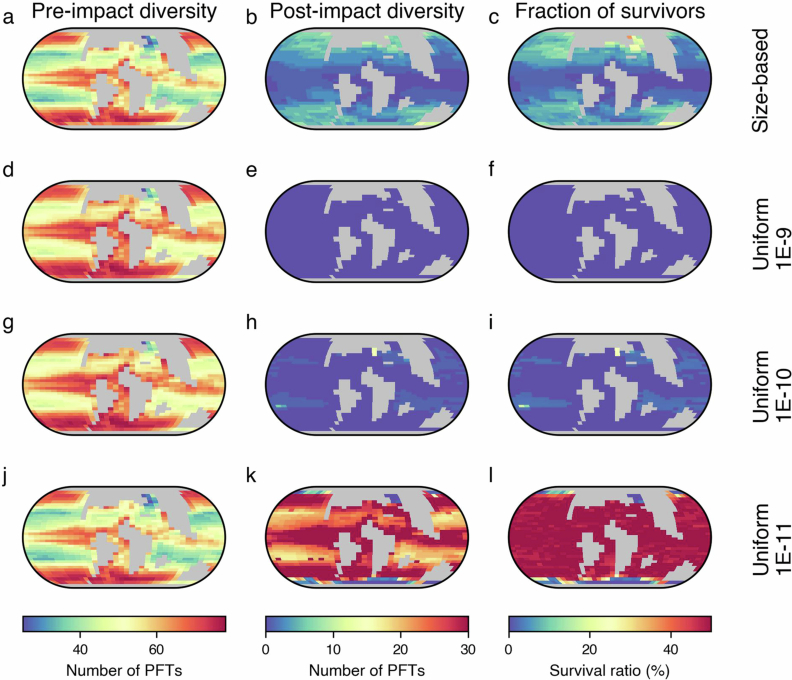
Simulated extinction patterns under different extinction threshold approaches. **(a-c)** Pre-impact diversity, post-impact diversity, and fraction of survivors using size-based extinction thresholds (as in Fig. 2). **(d-f, g-i, j-l)** use uniform extinction thresholds of 1 × 10^−9^, 1 × 10^−10^, 1 × 10^−11^ mmol C m^−3^, respectively, applied to all plankton functional types (PFTs). Only the size-based approach (top row) reproduces the observed extinction selectivity, while uniform thresholds fail to capture realistic extinction patterns.

In response to environmental changes, the model shows strong extinction selectivity, with a 78% loss of plankton functional types (PFTs; Fig. 2f) and a consequent 99.5% reduction in global primary production and carbon export (Fig. 2g,h). Only small-sized plankton (less than or equal to 2.5 μm) survived, particularly in the high latitudes (Fig. 1b,c). Extinction magnitude also varied across plankton functional types (Fig. 1d), with foraminifera experiencing complete extinction (100%), followed by other zooplankton (84%), with the lowest rates in mixotrophs (69%) and phytoplankton (65%). Although the model resolves functional diversity rather than taxonomic diversity, these results reproduce the extinction severity observed in the fossil records^7,19,21^ and the collapse in CaCO_3_ flux export^13^. The simulated extinction ratio of planktic foraminifera and nannoplankton is slightly overestimated, probably due to the lack of coastal/neritic trait in the model. The preferential survival of small plankton at high latitudes also agrees with the fossil record^4,5^ (Extended Data Fig. 5). Crucially, these results appear in the model only when extinction thresholds scale with body size. Simulations using uniform thresholds across all plankton types fail to reproduce the observed extinction-selectivity patterns (Extended Data Fig. 6). These findings demonstrate the key role of body size in determining the extinction pattern of marine plankton ecosystems across the K–Pg boundary.

Despite the simulated loss in functional diversity, plankton biomass and export production recovered rapidly with the return of light, causing a global plankton bloom (Fig. 2g,h). This bloom is driven primarily by mixotrophs, which outcompeted phytoplankton (Extended Data Fig. 5), even though the model assumes their photosynthesis and grazing efficiency to be 70% lower than those of specialized autotrophs or heterotrophs. This rapid resurgence in productivity and community structure change within years of the K–Pg boundary are in line with organic biomarker records^5,8,42^ and sedimentary evidence^5^, indicating that cyanobacteria outcompeted other eukaryotic phytoplankton in the earliest Danian^6,42–44^.

**Extended Data Fig. 7:**
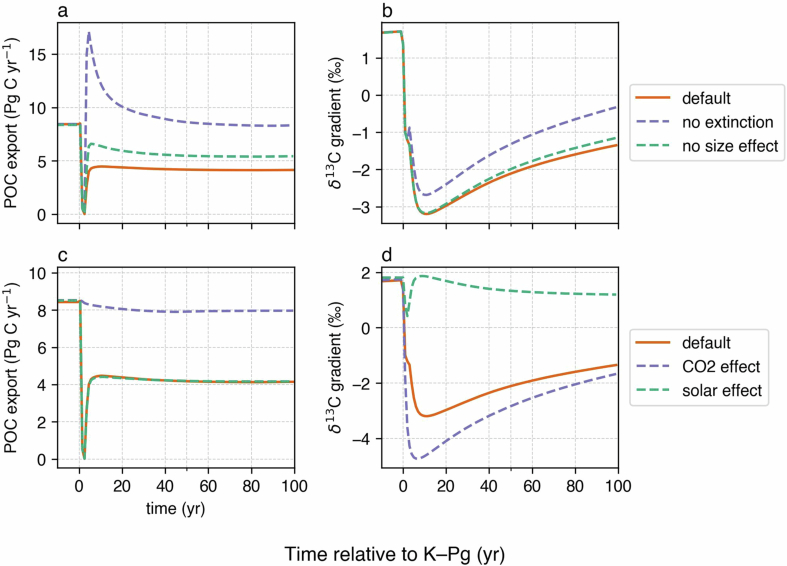
Simulated carbon export and carbon isotope excursion in sensitivity experiments. The default runs in both rows (solid lines) are identical to the combined experiment report in main text. These runs have size-based plankton extinction mechanism and size-based ratio of POC/DOC export. Sensitivity experiments in the first row show that disabling extinction causes an overshoot of carbon export flux as in Brugger et al. (2021). Making the POC/DOC export ratio independent of plankton body size could similarly partially prevent the collapse of biological pump. Both effects are reflected in the carbon isotope gradient (b). The second row examines the effect of solar radiation and CO_2_ forcing. The default run is forced with both of solar radiation and CO_2_ forcing, whilst the sensitivity run (dashed line) only has one of each forcing enabled. Figures (c-d) show that solar radiation change is the primary cause of productivity collapse. The observed reduction of the δ^13^C vertical gradient reflects not only a weakened biological pump, but also the injection of light CO_2_ into the atmosphere.

The collapse of plankton size structure reduces the biological pump efficiency by 30% in the model (Supplementary Information), resulting in greater nutrient retention in the upper ocean than pre-impact conditions (Extended Data Fig. 7). However, we do not observe the primary and export production overshoot reported by Brugger and colleagues^29^. We attribute this difference to the lack of explicit plankton extinction mechanism in their model, as EcoGENIE showed a similar productivity overshoot when the newly introduced plankton extinction mechanism is disabled (Supplementary Information and Extended Data Fig. 7).

**Extended Data Fig. 8:**
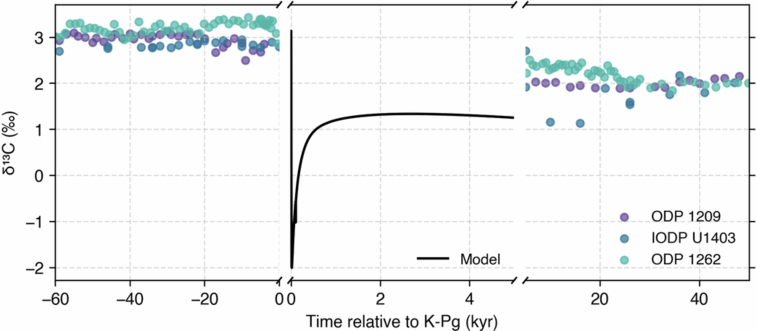
Modelled global mean surface ocean carbon isotope excursion compared with high-resolution bulk carbonate carbon isotopic data from Hull et al.^10^. Data shown were collected from Sites ODP 1209 (Shatsky Rise), ODP 1262 (Walvis Ridge), and IODP U1403 (J-Anomaly Ridge)^10^. The model shows reasonable agreement with proxy data, but we caution that the simulated ocean surface carbon isotope values during the 5 kyrs after the K-Pg boundary are ultimately subject to the total carbon emission and associated carbon isotope value set in the model. Brugger et al.^29^ performed model sensitivity analysis to show that the total amount of 1615 Pg C matches the proxy record the best. Our value of 1750 Pg C generally agrees with Brugger et al.^29^ but suggests a higher carbon emission is required to fully match the observation given the same carbon isotope signature. We did not conduct further sensitivity experiment as this is out of the scope of our study.

**Extended Data Fig. 9:**
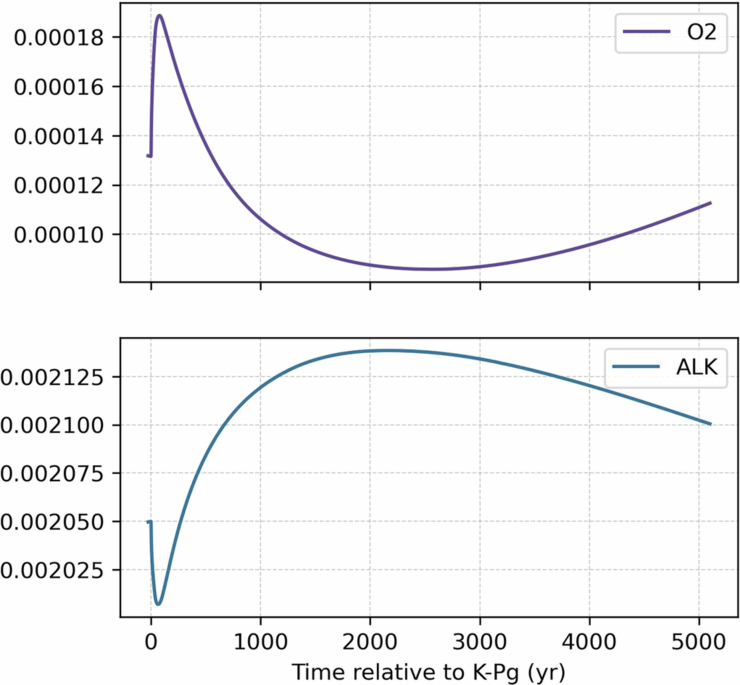
Modelled benthic oxygen and alkalinity after the K-Pg boundary. Modelled benthic oxygen and alkalinity concentrations (mol kg^−1^) after the K-Pg boundary. The ‘benthic’ here is defined here as ocean depths greater than 2000 m.

EcoGENIE also compares well with carbon cycle proxies. The model reproduces the carbon isotope record of the Late Cretaceous (Supplementary Fig. 5) and captures a K–Pg carbon isotope excursion of similar magnitude to that seen in the δ^13^C proxy data^10^ (Extended Data Fig. 8). Henehan and colleagues^12^ combined carbon isotope and pH data to constrain the post-extinction carbon export production level to 50% of the pre-impact level. This is close to our result of 61% based on a dynamic plankton model. Finally, the model suggests an increase of benthic alkalinity (deeper than 2,000 m) after the K–Pg (Extended Data Fig. 9), which aligns with the observed high foraminifera preservation and low fragmentation in the early Danian^45^. These agreements with multi-faceted K–Pg observations further confirm the validity of our transient K–Pg extinction experiments.

## Drivers of selective extinction

**Fig. 3:**
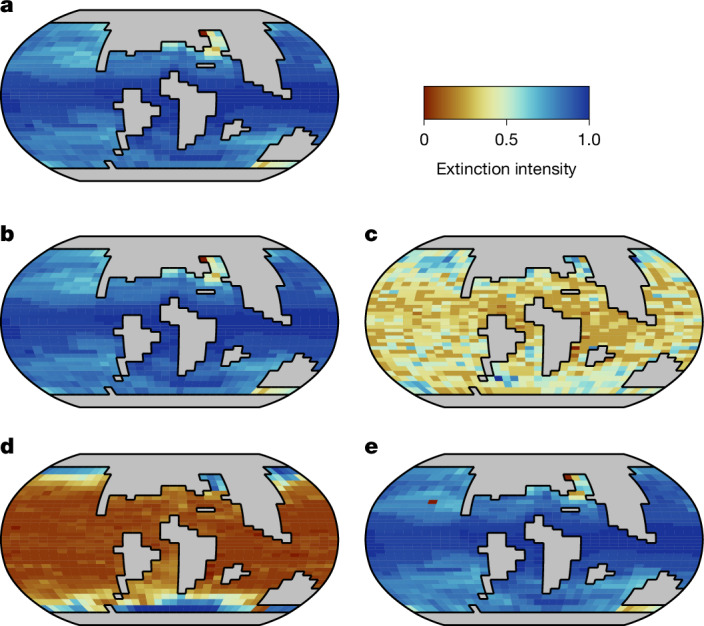
Breakdown of the drivers of K–Pg extinction patterns. **a**–**c**, Extinction intensity (main result (**a**), solar forcing (**b**) and CO_2_ forcing (**c**)) is measured by the ratio of extinct PFTs to the pre-impact plankton diversity. **d**,**e**, Subplots further disentangle the light (**d**) and temperature (**e**) effects from solar radiation change by forcing the plankton ecosystem with unchanged sea surface temperature (light effect only) and PAR (temperature effect only). These figures show that light was the ultimate driver of K–Pg extinction.

The overall model–data agreements allow us to explore the climatic drivers of the K–Pg extinction in detail. Specifically, we ran sensitivity experiments isolating the impacts of solar radiation and CO_2_ forcing to evaluate their individual contributions (Fig. 3). The results show that solar radiation alone reproduces most of the extinction patterns present in the combined forcing experiment, whereas CO_2_ forcing has a negligible impact despite a pronounced emission rate. This indicates the primary role of reduced solar radiation in causing the K–Pg extinction. However, we acknowledge that our model may underestimate the overall impact of volatile gas due to the lack of an explicit mechanistic calcification process and sulfuric and nitric acids^46^.

The influences of solar radiation change on plankton responses are multi-dimensional, affecting temperature, light availability and ocean mixing through changes in the density of the ocean. To further understand the contributions of these different factors, we first forced the whole plankton community with a constant pre-impact annual mean sea surface temperature while keeping other parameters the same, exploring the impacts of the global winter. Against expectations, temperature had a limited impact on extinction patterns (Fig. 3e), despite temperature’s exponential effect on growth rates^47^ and the well-documented correlation between temperature change and extinction risk^31^. By contrast, when the plankton community was forced with pre-impact photosynthetic activity radiation (PAR), pre-K–Pg plankton diversity was sustained except in high latitudes such as the Southern Ocean and North Pacific, where temperature change also contributed to extinction. This result indicates the primary role of light limitation in causing productivity loss and subsequent mass extinction through trophic cascades (Fig. 3d).

**Fig. 4:**
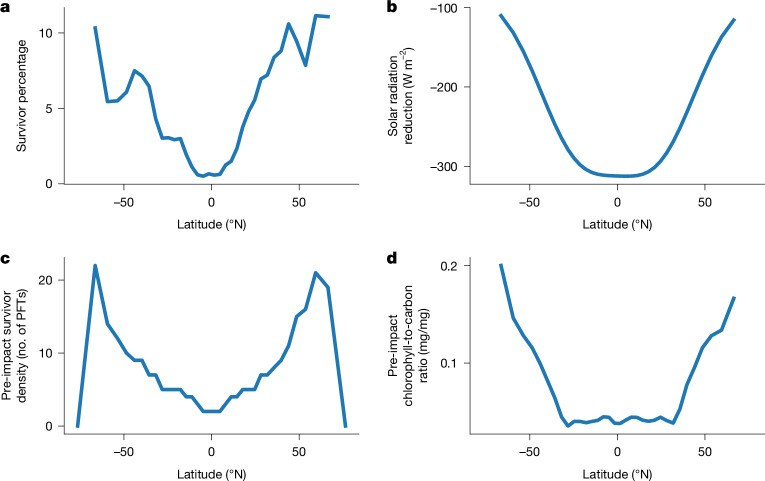
Latitudinal selectivity of plankton extinction in the model. **a**, Zonal mean survivor percentage of total PFTs. **b**, Absolute solar radiation reduction in the first year following the K–Pg, which is highest in the low latitudes. **c**, Zonal mean of all plankton survivor’s richness. **d**, Zonal mean total chlorophyll-to-carbon ratio implies the plankton survivors’ ability to live in low-light environments.

As light deficiency emerges as the primary driver of the simulated extinction, regional differences in light reduction explain the highest extinction risk in the tropics, where irradiance drops the most (Fig. 4a,b). This mechanism also accounts for the higher extinction rates of modelled zooplankton compared with mixotrophs and phytoplankton (Fig. 1d), reflecting their different ability in using light energy. Planktic foraminifera, which belongs to the microzooplankton community, have additional metabolic costs associated with calcification, making them particularly vulnerable relative to other zooplankton. For phytoplankton, light demand seems to be more important than body size, because smaller nannoplankton went extinct, whereas larger diatoms survived the extinction, probably due to different light demand and geographical distribution.

Besides the difference in absolute light change, the pre-impact distribution and photo-acclimatization of phytoplankton might also contribute to the extinction selectivity. For instance, the simulated plankton survivors tend to live in the high latitudes and have high chlorophyll-to-carbon ratios (Fig. 4c,d), indicating a greater energy investment in light absorption and pre-acclimatization to low-light conditions^48^. This observation corroborates previous hypotheses for nannoplankton extinction selectivity^7,15^, and corresponds to fossil evidence of higher extinctions in groups reliant on high light conditions, including coccolithophores, symbiont-bearing foraminifera^21^, colonial corals^49^ and photosymbiotic rudist bivalves^49^, whereas diatoms and dinoflagellates are less impacted due to their tolerance to low-light turbulent environments^50^.

Darkness and body size-based thresholds collectively explain the observed marine plankton extinction selectivity. These drivers can be unified within an energy balance framework: darkness results in a reduced environmental energy supply, whereas body size sets energy demands. This framework can be applied across organisms, including unexamined traits and higher trophic levels. For instance, many diatoms and dinoflagellates enter dormancy to reduce their metabolic cost, and hence their energy demand, thereby increasing their chance of survival^51^. All foraminiferal and calcareous nannoplankton survivors in the fossil record have several energy pathways (for example, mixotroph and detritus feeding)^4,21^. Similarly, traits of surviving sharks, rays, molluscs (for example, feeding mode and mobility)^52,53^ and benthic foraminifera (for example, size)^54^ also reflect strategies of energy (food) acquisition and requirement. These examples highlight that differential energy supply, acquisition and demand provide a universal explanation for the selective pattern of the K–Pg marine extinction.

## Reconciling with previous studies

**Extended Data Fig. 10:**
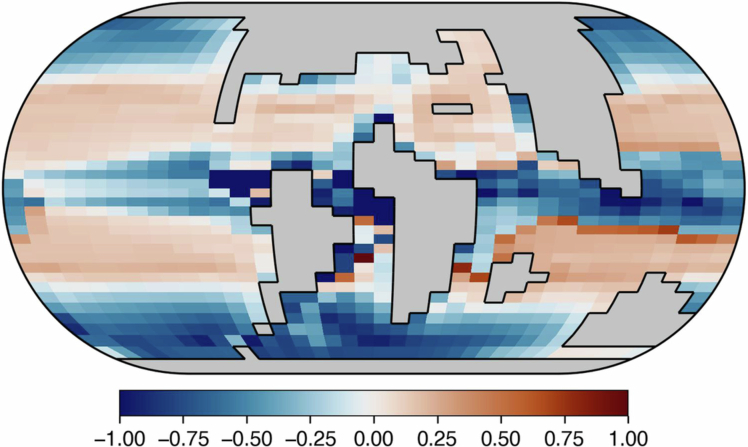
Simulated change in marine particulate organic carbon export 5,000 years after the K-Pg. Subtropical gyres are characterized by higher carbon export after the extinction events, whereas other regions record a decline in export.

Our results of light loss and the subsequent productivity collapse in causing marine extinction is consistent with the ‘impact winter’ theory initially proposed by Alvarez and colleagues^23^. However, studies based on benthic foraminiferal community^55^ and fish teeth data^56^ suggest limited evidence for productivity decline in deeper ocean communities. This apparent discrepancy can be reconciled by our model results, which show a rapid (year-scale) post-K–Pg productivity recovery^29,42,57^ (Fig. 2), probably too brief to be fully captured by the relatively coarse temporal resolution of most geological proxies, which often average over several millennia due to low sedimentation rates or non-deposition after the boundary combined with mixing due to bioturbation. Once the model reaches a quasi-steady state (5,000 years after the impact), the simulated productivity trends agree with observational data^25,43,45^ and reveal similar spatial heterogeneity (Extended Data Fig. 10). In the subtropical gyres, carbon export increases due to enhanced mixing and nutrient supply to the surface (Extended Data Fig. 10). By contrast, the upwelling regions and high latitudes which were already eutrophic, experienced biodiversity loss, reducing nutrient uptake and carbon export efficiency (Extended Data Fig. 10).

Previous studies have used the vertical δ^13^C gradient to infer changes in the biological pump at the K–Pg boundary^13,21,58^. However, this proxy is influenced not only by biological pump efficiency but also by other processes. For instance, our simulated changes in the δ^13^C gradient across the K–Pg do not solely reflect variations in carbon export productivity but is also affected by the injection of isotopically light carbon from wildfire and/or fossil carbon burning (Extended Data Fig. 7). Such an argument is supported by the recent evidence of rapid warming during the first decade of Danian^59^ and was proposed in earlier studies^29,46^. Note this argument is not in conflict with a previous study^12^ suggesting the impacts of weakening biological pump on δ^13^C gradient at millions of years after the boundary, because the solubility effect could be buffered by photosynthesis or sedimentary burial within the million-year window. Therefore, we suggest relying solely on δ^13^C data as a proxy for marine productivity may underestimate the timing of recovery of the biological pump^60^.

Our results do not support ocean acidification as the primary driver of the K–Pg extinction^12^. The EcoGENIE model reproduces the observed trends in foraminiferal and nannofossil extinction successfully without implementing any acidification impact. Moreover, the modelled saturation state of calcite and aragonite remains above 1 in most regions (Extended Data Fig. 4), similar to a previous modelling study that relied on a more comprehensive analysis of CO_2_ and SO_2_ forcings^29^ and more simplistic box models^46^. Modelled acidification is most pronounced in the polar regions, whereas some calcifiers (for example, coccolithophores^7^) experienced lower extinction in high latitudes. Overall, although we cannot rule out a potential synergistic effect of ocean acidification, our results suggest that, rather than changes in carbonate chemistry alone, darkness and starvation probably played a dominant role in the extinction of marine calcifiers at the K–Pg boundary.

## Conclusion

We address the long-standing question of the physiological drivers underlying extinction patterns in the aftermath of the K–Pg mass extinction using a coupled a trait-based ecosystem model with independent climatic forcings to simulate marine plankton community dynamics at century scale. By introducing a body-size-dependent extinction threshold, our model reproduces successfully key features observed in the K–Pg fossil record. We are therefore able to reconcile selective extinction patterns and the dominance of small plankton and mixotrophs in the post-K–Pg ocean. Sensitivity experiments identify darkness arising from global atmospheric aerosol cover and the size-based extinction threshold as the primary drivers of marine extinction selectivity. The pre-impact distribution of taxa and their photo-acclimatization during the impact winter modulated ecological and geographical outcomes. Trait-based ecosystem modelling provides a powerful hypothesis generator for investigating past events with fossil constraints, identifying mechanisms that drive vulnerability and drivers for selective survival in response to environmental changes.

## Methods

### cGENIE Earth System Model

We use the cGENIE Earth System Model of Intermediate Complexity (EMIC) to simulate the climate changes and plankton ecosystem dynamics across the K–Pg boundary. The cGENIE model comprises three-dimensional ocean physics (GOLDSTEIN) and marine biogeochemistry (BIOGEM) (for example, C, P, O, Fe, Si), a two-dimensional atmosphere (EMBM), a trait-based plankton ecosystem model (EcoGENIE) and a sediment component (SEDGEM). The application of this model to the modern climate has demonstrated its ability to capture realistic ocean physics including deep-water formation and large-scale ocean circulation (Supplementary Figs. 6 and 7).

Previous K–Pg studies have used the cGENIE model to constrain the post-impact carbon cycle (on million-year timescales) using proxy data^12^ and to assess the impact of volcanic sulfur deposition from flood basalt eruptions^61^. These studies provide an important foundation for us because the Late Cretaceous model has been tuned to reproduce realistic climate (Extended Data Fig. 1), ocean circulation (Supplementary Fig. 8) and ocean biogeochemistry (Supplementary Figs. 5 and 9). However, these studies were assessed in steady-state conditions and without an explicit ecosystem component. In this work, we ran the cGENIE model with transient solar radiation, pCO_2_ and nutrient forcings and coupled with EcoGENIE at a century scale, expanding on these studies. The ecosystem model has a small timestep (7.3 h) to capture the trophic cascaded suggested by Alvarez and colleagues^23^. The plankton ecosystem, as the base of the marine food web, also provides an indication of the status of, and changes to, higher trophic levels.

### Trait-based plankton ecosystem model

EcoGENIE is a trait-based mechanistic (forward) plankton ecosystem model integrated within the cGENIE framework. ‘Traits’ here refer to organism characteristics that influence ecophysiological processes and fitness. EcoGENIE receives environmental inputs, such as temperature, light availability and nutrient concentrations, from cGENIE’s physical (GOLDSTEIN) and biogeochemical (BIOGEM) modules. These inputs inform plankton food web dynamics within a variable mixed layer, driving the biomass evolution for each plankton functional type.

EcoGENIE captures ecosystem dynamics explicitly by simulating each plankton’s metabolic processes including photosynthesis, grazing, respiration and mortality (Supplementary Information). These processes are governed by abiotic factors (temperature, nutrient availability and light), plankton traits (for example, size, heterotrophy, calcification and Chl:C ratio based on the photo-acclimatization model of Geider and colleagues^62^) and biotic interactions (for example, competition and predator–prey relationships) (Supplementary Information). Through these interactions, the plankton community structure emerges naturally, reflecting resource competition and adaptation to specific local environmental conditions. Because the model aggregates species into functional types (defined by similar traits), the diversity represented in this study is functional rather than species-specific and arises directly from environmental selection rather than imposed a priori.

The emergent plankton ecosystem structure influences organic matter export. Specifically, larger plankton typically have a higher particulate organic carbon (POC) to dissolved organic carbon (DOC) export ratio relative to smaller plankton (Supplementary Fig. 1), thereby affecting the efficiency of the biological carbon pump and the overall biogeochemical cycling of the marine environment.

EcoGENIE simulates the plankton community as vertically integrated over the mixed layer. This simplified strategy improves the computational efficiency and reflects the nature that most plankton abundance^63^ and diversity (particularly phytoplankton diversity^64^) are found in the upper ocean. The MLD is calculated using a Kraus–Turner scheme and influences nutrient supply and light availability (with deeper MLD corresponding to higher nutrient supply and lower light availability). In the modern ocean, the model’s MLD compares well with the ECCOv.4 data product (Supplementary Fig. 6). A recent study^35^ further supports the use of this simplified strategy, showing similar model performance compared with higher-complexity plankton models in response to climate change. However, EcoGENIE does not explicitly resolve the direct impact of water structure change (for example, destratification) on plankton specific vertical habitats. This represents a potentially important limitation, as the physical loss of depth habitats with higher mixing may have contributed to the K–Pg extinction selectivity, particularly for deep-dwelling zooplankton^65^.

A key advantage of EcoGENIE is its trait-based feature, which uses allometric (size-based) relationships to parameterize plankton ecology (for example, growth rate; Supplementary Fig. 1), thereby avoiding subjective bias or over-reliance on modern taxa, making it particularly well-suited for deep-time studies^66^. The trait-based framework also allows us to account for the trait diversity of marine plankton in our model. For instance, the recent development of the calcification trait enables us to model calcareous zooplankton (that is, foraminifera) by modifying generic zooplankton’s ecological parameters given assumed trade-offs^3^. The model also shows realistic trait distributions in the modern^3^ and the Last Glacial Maximum^35^ oceans, constraining the simulated latitudinal distribution of functional diversity and extinction selectivity in taxon distributions (Supplementary Fig. 10). However, the model does not yet explicitly simulate shell mineralogy and sensitivity to changes in carbon chemistry. As a result, our interpretation of calcareous plankton’s extinction risk is probably conservative. Instead, calcite production in our model is parameterized using a constant CaCO_3_:POC rain ratio.

### Extinction process

Marine biogeochemical models typically simulate plankton population biomass without explicitly including the extinction process. To address this limitation, we incorporate a size-dependent extinction mechanism into EcoGENIE based on a population biomass threshold. We link extinction to biomass because many potential surviving strategies (for example, adaptation) are dependent inherently on the effective population size.

For a population, extinction occurs when the last individual disappears. Therefore, to sustain ecosystem functions, the plankton population size must be greater than one individual, and biomass must exceed a critical threshold (referred to as the extinction threshold), equivalent to the minimum carbon biomass per individual. Such a minimal biomass threshold is size dependent^67^, reflecting the nature that minimal energy demand (for resting metabolic activities) of an individual scales with its body size^47,68^ (Supplementary Fig. 1). In our size-based model, such implementation is natural because the model pre-defines the biomass content of each cell (termed as cell quota) for each plankton functional type, which is linked to its biovolume using an observed scaling law^67^. When biomass falls below the extinction threshold, the model functionally ‘kills’ the plankton group by disabling key processes such as photosynthesis and grazing. We do not include the re-diversification process due to the uncertainty surrounding the mechanisms of evolution and thus do not assess the potential impact of adaptive evolution. We also do not assess the role of ocean transport in causing the extinction pattern as the K–Pg oceans were characterized with strong mixing and previous study^69^ has suggested environmental selection places stronger constraints than dispersal.

It is critical to note that body size and trophic strategy are the main traits used to characterize our plankton community. We do not assign differential sensitivities to temperature or light across plankton functional types, nor do we impose a pre-defined environmental niche range. Consequently, extinction risk is determined solely by the emergent biomass. However, the model does allow the differentiation of photosynthetic ability through the photo acclimatization processes, which dynamically adjust the Chl:C ratio of autotrophic plankton according to light environments. We expand and the extinction mechanism details in the Supplementary Information.

### Reporting summary

Further information on research design is available in the Nature Portfolio Reporting Summary linked to this article.

## Online content

Any methods, additional references, Nature Portfolio reporting summaries, source data, extended data, supplementary information, acknowledgements, peer review information; details of author contributions and competing interests; and statements of data and code availability are available at 10.1038/s41586-026-10541-4.

## Supplementary information

Supplementary Information This file contains Supplementary Methods describing the EcoGENIE model configuration, extinction setup, climate boundary conditions and model validation; Figs. 1–10 support the model setup, ocean physics, ecology, and biogeochemistry; Table 1 lists plankton functional type size ranges

Reporting Summary

Peer Review File

## Data Availability

The model outputs are available at Zenodo (10.5281/zenodo.17742290)^70^. The ForCenS data (10.1594/PANGAEA.873570) were used to validate modern foraminifera trait distribution.

## Code availability

The model source code is available at https://github.com/ruiying-ocean/cgenie.muffin/tree/rui_kpg. The specific configuration can be found at https://github.com/ruiying-ocean/cgenie.muffin/tree/rui_kpg/genie-userconfigs/PUBS/submitted/Ying_et_al.Nature.2025. The documentation of how to install and compile the model can be found at https://github.com/derpycode/muffindoc and https://github.com/ruiying-ocean/install.cgenie. Scripts and data for reproducing figures are available at https://github.com/ruiying-ocean/kpg_selectivity. A open-source python package cgeniepy^71^ is available for analysing model outputs.
