# Supplementary material for: Darkness and body size shaped end-Cretaceous marine extinction patterns
Source: Nature. 2026 May 27;655(8124):957–62. doi: 10.1038/s41586-026-10541-4 (PMC13391373; doi:10.1038/s41586-026-10541-4)
Supplement: Supplementary file 1 — This file contains Supplementary Methods describing the EcoGENIE model configuration, extinction setup, climate boundary conditions and model validation; Figs. 1–10 support the model setup, ocean physics, ecology, and biogeochemistry; Table 1 lists plankton functional type size ranges [file 41586_2026_10541_MOESM1_ESM.pdf]

nature portfolio

https://doi.org/10.1038/s41586-026-10541-4

Supplementary information

Darkness and body size shaped end-Cretaceous marine extinction patterns

In the format provided by the authors and unedited

EcoGENIE model

Trait-based framework

Marine ecosystem and biogeochemical models simulate plankton biomass using a series of process-based differential equations. For instance, in EcoGENIE, phytoplankton biomass is determined by the balance of photosynthesis (μ), grazing by heterotrophs (g), mortality (*mort*), and respiration (*respir*). Zooplankton biomass depends similarly on gains and losses from grazing (g), mortality (*mort*), and respiration rate (*respir*). Among these processes, photosynthesis is a function of photosynthetically available radiation (PAR), nutrient availability (N), temperature (T), and plankton biomass (P). Grazing is a function of temperature (T), predator and prey biomass. The mortality rate linearly scales with biomass; respiration is temperature-dependent and scales with plankton biomass.

$$\frac{\mathrm{dP}}{\mathrm{dt}} = \mu(PAR, N, P, T) - \mathrm{g}(P, predator, T) - \mathrm{mort}(P) - respir(P, T)$$

$$\frac{\mathrm{dZ}}{\mathrm{dt}} = g(food, Z, T) - \mathrm{g}(Z, predator, T) - mort(Z) - respir(Z, T)$$

The parameterisations of these processes for different planktons are defined by traits (noted as θ) trade-offs, which are mostly associated with plankton Equivalent Spherical Diameter (ESD) as the following equations, in which a and b are coefficient of size-scaling rules.

$$\theta = aV^b$$

$$\mathrm{V} = \frac{\pi}{6}\mathrm{ESD}^3$$

For instance, maximum photosynthesis rate decreases with body decreases and vice visa for maximum nutrient uptake rate (Fig. S1). These size scaling parameters are taken from Marañón et al.[67] data compilation.

Some plankton traits in the model are represented as net effects on key physiological processes[71]. For instance, calcification increases respirational costs but protect calcareous plankton from virus and predator attack, reflecting as a lower mortality rate and grazing risk in the model. Similarly, the spine trait enhances spinose plankton's grazing range at the cost of higher respirational rate. Symbiosis, functionally equivalent to mixotrophy in the model, enables zooplankton to photosynthesize and graze, though at lower efficiencies than specialised autotrophic or heterotrophic plankton.

Regarding the potentially important traits for survival during the K-Pg extinction, all phytoplankton in the model are assigned the same light sensitivity (i.e., the initial slope to photosynthesis-irradiance curve, the maximum Chl:C ratio) and temperature limitation (defined by an Arrhenius-like equation with a Q_{10} of 1.65). Photosynthesis rate is further regulated by iron availability and a dynamic Chl a to C ratio, calculated using a revised Geider et al.[62] formulation. All the plankton groups are also initialised with the same biomass

and are not constrained by a a pre-defined niche range. Therefore, extinction in the model is only determined by biomass decline, without imposing trait-specific conditions.

As cGENIE explicitly resolve climate and ocean environments, the ecosystem dynamics above are characterised by geographic differences (Extended Data Fig.4), a key advancement over previous modelling studies (e.g., the idealised model in Gibbs et al.[4]). These ecosystem dynamics determines the global biogeochemical cycles, including the simulated POC export and DIC carbon isotope distribution. The plankton size in the model also influences the fraction of organic carbon export out of the euphotic zone (Fig. S1), allowing us to capture the potential effect of post-KPg picoplankton in trapping ocean surface nutrients.

Extinction and size-based biomass threshold

EcoGENIE simulates the biomass gain (e.g., photosynthesis, grazing gain) and loss (e.g., respiration) of marine plankton. However, marine biogeochemical models typically do not set a minimal value of plankton biomass (*P*) and will allow phytoplankton blooms despite showing extremely low biomass. Therefore, these models inherently prevent true extinction, making them unsuitable for studying mass extinctions. To overcome this problem, we introduce a carbon biomass threshold below which source processes (i.e., grazing and photosynthesis) are suppressed. This threshold acts as an extinction criterion, allowing the model to capture biomass collapse and community loss.

$$\mu(PAR,N,P,T) = \begin{cases} \mu(PAR,N,P,T), \text{if} P > P_{crit} \\ 0, if P < P_{crit} \end{cases}$$

$$g(food,Z,T) = \begin{cases} g(food,Z,T), if Z > Z_{crit} \\ 0, if\ Z > Z_{crit} \end{cases}$$

This carbon biomass threshold is defined as the biomass content of an individual since the individual is the smallest unit capable of sustaining metabolic and ecosystem functions. In EcoGENIE, this individual biomass content is already predefined through the Droop cellular quota[72] (i.e., the elemental concentration in a single cell of a given size), which regulates key processes such as photosynthesis, grazing efficiency, and nutrient uptake[2]. The cellular quota itself in the model follows an allometric power law relationship with body size as empirical observations[67] (Fig. S1). Therefore, the biomass threshold and extinction vulnerability increase with body size. Such an implementation also does not require any new parameters in the model, while allowing us to uniquely link plankton biomass, ecological traits, and their extinction vulnerability.

A diverse plankton community configuration in ECOGENIE

To study the selectivity of the K-Pg extinction, we created a configuration with 112 plankton functional types (PFTs), including 32 phytoplankton, 32 zooplankton (non-calcifying), 32 mixotrophic plankton, 8 symbiont-obligate foraminifera, and 8 symbiont-barren foraminifera (calcifying zooplankton). This configuration forms a sufficiently large pool of plankton ecological traits, allowing the model to simulate natural selection processes under changing environmental conditions. For comparison, most biogeochemical models contain only 2 to 5

plankton groups[73]. Including 112 PFTs makes the model computationally demanding, substantially slowing simulations. As a result, we used this configuration only in the 200-year transient experiments.

For the pre-KPg Maastrichtian experiments, we used the standard ECOGEM configuration from Ward et al.[2] (8 phytoplankton and 8 zooplankton) to spin up the marine biogeochemical cycle for the initial pre-perturbation state. All simulations use PO_4 and Fe as the limiting nutrients for phytoplankton growth.

The parameterisation of each plankton type in this new configuration has been described separately in previous studies. The parameterisation for phytoplankton and zooplankton follows Ward et al.[2]. The mixotrophic plankton parameterisation was described by Ward et al.[74] and used in Reinhard et al.[75]. The foraminifera settings are the same as Ying et al.[35]. Here, we selected only two major Late Cretaceous ecogroups (symbiont-bearing non-spinose, such as *Racemiguembelina*; symbiont-barren non-spinose, such as *Guembelitria, Heterohelix*). Each plankton functional group is represented across specific size classes (Table S1), which determine other traits, including maximal growth rate and grazing preference based on ecological allometric rules (Fig. S1). Consistent with observations in the modern ocean, symbiont-bearing foraminifera are larger than the symbiont-barren foraminifera[76] , and there is evidence in the late Cretaceous for it to be the same with large genera bearing symbionts[76,77]. This trait-based modelling framework is further explained in detail in Ward et al.[2] and Ying et al.[3].

Model experiment design

Maastrichtian experiment

We first run a Maastrichtian (70 Ma) spin-up simulation for 10,000 years. This configuration is based on previous cGENIE K-Pg studies. Bathymetry and continental configuration are taken from Schmidt et al.[61] (Fig. S2) and have 36×36 horizontal grids and 16 vertical layers. Similarly, plenary albedo and wind forcings are regridded from a general circulation model and are not modified here. The solar constant of Maastrichtian was set to 1360.33 W m^{-2}, reduced by 0.56% relative to the modern state. The Maastrichtian obliquity at the time of the impact is not well defined and hence fixed to pre-industrial value (23.5°), following the previous K-Pg modelling study of Brugger et al.[41] . Atmospheric pCO_2 was set to 834 ppm (i.e., 3 times pre-industrial levels), consistent with boron isotope estimates[12]. The carbon isotope value $\delta^{13}C_{CO2}$ was -4.5‰ as defined by Henehan et al.[12]. Other climate boundary conditions controlling atmosphere, ocean, and sea-ice physics are the same as the modern setup described in Cao et al.[78]. The ecosystem relied on Ward et al. (2018) configuration (8 Phytoplankton, 8 zooplankton; Extended Data Fig. 1) to speed up the spin-up process. We masked the Arctic Ocean in our results because cGENIE does not realistically represent this area due to its limited spatial resolution.

The biogeochemical tracers simulated include carbon, phosphate, oxygen, and iron, but do not consider other important climate-active gases such as nitrogen and sulphur. The carbon cycle has a uniform $CaCO_3$:POC rain ratio (0.2) as estimated in the Paleocene[79]. In addition,

we include a 20 Tmol yr^{-1} global weathering flux as in Henehan et al.[12] to improve the alkalinity representation. Here, we also simulated the iron cycle with the dust deposition flux regridded from the postdam2 atmosphere model[41] using Climate Data Operator while parameters such as solubility, scavenging rate and ligand tracer initial conditions are kept the same as modern[80,81]. Dust deposition is highest around Africa and South America and lowest in the central Pacific Ocean (Extended Data Fig. 3).

The Maastrichtian and post K-Pg model experiments are run with a hybrid timestep framework to accommodate the differing temporal scales of the different Earth System components. The atmospheric component is integrated with a timestep of 0.76 days, corresponding to 480 steps per model year (365.25 days). The ocean and sea-ice components operate on a coarser timestep of 3.8 days, five times longer than that of the atmosphere. To better capture rapid biological dynamics, the marine ecosystem module employs a much finer timestep of approximately 7.3 hours, whereas the ocean biogeochemistry module is updated every 7.6 days. The sediment module evolves on the longest timescale in the system, exchanging fluxes with the overlying ocean once per model year. Together, these settings allow for a realistic but efficient representation of climate, ocean environment, and plankton ecosystem dynamics over the simulated century scale.

K-Pg experiment

Starting from the spin-up model, we ran a transient experiment with the 112-plankton configuration for 200 years. We only ran the model for this short time because of the high computational cost of running such high plankton diversity configuration. However, we ran extra transient experiments to investigate ocean biogeochemistry based on the surviving plankton.

During the first 100 years, the system was let to stabilise under the new configuration. At the beginning of the second 100 years, we introduced three climate perturbations designed to mimic the effects of the impact: a decline in solar radiation, an abrupt CO_2 emission, and an extra nutrient flux. For the solar radiation reduction, we applied the forcing from Senel et al.[11] to overcome the limitations of cGENIE simplified atmosphere (2D energy moisture balance model), which cannot mechanistically simulate solar radiation changes. The Senel et al.[11] study used sediment records from the K-Pg sections of the Tanis site (United States) to constrain the size of emitted particles from the Chicxulub impact, combined with a General Circulation Model to simulate the effects of injected dust, soot, and sulphur. Their results show that photosynthetic activity radiation (PAR) dropped to zero for less than 2 years and recovered to pre-impact levels by the 4th year post-impact. To incorporate this hypothesis into cGENIE, we calculated the annual average change in net shortwave radiation and accordingly scaled the model solar constant(set at 1360.33 W m^{-2} for the Late Cretaceous), neglecting seasonal variations associated with the impact (i.e., suggested to occur during a "boreal spring")[82]. We also assumed a globally uniform distribution of ejecta materials, consistent with Senel et al.[11] , who suggest rapid global dispersal within 1 day after the impact. The resulting relative solar radiation levels for the first four years post-impact are

roughly 0%, 20%, 80%, and 100% of the pre-impact value, respectively. PAR at each grid cell was calculated as 43% of the incoming solar radiation.

To assess the impact of CO_2 emissions, we introduced 1,750 Pg C in the first year to match the rise of atmospheric CO_2 (from ~900 to ~1600 ppm) estimated by boron isotope data[12]. Recent studies support such a CO_2 emission, citing rapid warming within the first decade of the Danian [83]. We assumed the emission was spatially uniform, given the rapid diffusion and transport of vaporised carbonate and fuelled organic carbon. This carbon release falls within previous estimates between 0 to 4,115 Pg C[29,46,84] and is close to the carbon isotope constrained value (1615 Pg)[29]. The $\delta^{13}C$ of the emitted carbon was set to −27‰, a typical value for organic matter and fossil carbon, following previous model practice[29] . This approach reflects that most emitted carbon likely originated from organic material (1,500 Gt C from wildfires, 2500 Pg C from soil organic carbon, compared to 115 Pg C from carbonate rock as tested in Brugger et al.[29]). However, these values remain coarse estimates due to uncertainties in the magnitude and relative contribution of different carbon sources. The uncertainty of the carbon isotope values does not influence our conclusion, because the biological pump rapidly recovered, leaving the vertical carbon isotope gradient largely unchanged (Fig. 2).

The impact of dust provided an additional nutrient flux to the ocean surface[29]. To assess such impact on the observed selective extinction, we regridded the dust distribution from the Potsdam model (Brugger et al.[29])(Extended Data Fig. 3) into the cGENIE grid resolution, assuming the same fraction (Fe: 22.5%; P: 0.104%) and solubility (Fe: 0.2%; P: 2%). This is equivalent to an input of 5.38 Tmol Fe and 0.45 Tmol PO_4 in the first year after the K-Pg impact.

<u>Early Danian experiment</u>

To compare our model results with biogeochemical proxies, we further ran the model using only the surviving plankton functional types for 5,000 years following the K-Pg experiment. The modelled long-term decline in benthic oxygen is consistent with the benthic foraminifera[85,86], pyrite evidence[20], and sulphate isotope evidence[87,88] (Extended Data Fig. 9). The modelled benthic alkalinity also increased as shown by the observed high foraminifera preservation and low fragmentation in the Early Danian[45]. The recovery of surface carbon isotopic values in the model are comparable with the high-resolution proxy observations in Hull et al.[10] (Extended Data Fig. 8). However, the simulated $\delta^{13}C$ during the Early Danian is sensitive to the total carbon emitted into the atmosphere, as demonstrated by Brugger et al.[29]. We also note that our model focuses on hundreds and thousands of years after the boundary, whereas the low sedimentation rates of the limited open ocean sites cover, considering bioturbation, more than 1,000 years in one centimetre.

<u>Sensitivity tests of extinction drivers</u>

We ran a series of sensitivity experiments to investigate the primary drivers of extinction. First, we applied solar radiation and CO_2 forcings separately to isolate their individual impacts on the marine ecosystem. Second, we disentangled the relative contributions of temperature, light, and nutrient availability, following Wilson et al.[66]. Specifically, this was

achieved by imposing fixed pre-impact annual mean fields of temperature or PAR on the plankton community, thereby removing their respective influence on plankton activity. This approach allowed us to isolate the effects of temperature, PAR, and nutrients independently, quantifying their relative contribution to extinction pressures (Fig. 3). For example, if temperature change were the primary driver of extinction, then maintaining a fixed temperature field would prevent the collapse of plankton biomass, productivity, and other ecosystem functions in the model.

Sensitivity tests of extinction impacts

We tested the impact of extinction and plankton community size-structure collapse on the biological pump and vertical carbon isotope gradients by turning off the extinction mechanism or fixing the POC:DOC production ratio at a global average value (0.66). The results show that, without extinction, the biological pump rapidly recovers and even exceeds pre-impact values (Extended Data Fig. 7), consistent with results in Brugger et al. (2021). Similarly, making POC:DOC export ratio independent from plankton body size allows carbon export to be 30% (5.43 Pg C yr^{-1}) higher than the default one (4.15 Pg C yr^{-1}) 200 yr after the K-Pg boundary.

Sensitivity tests of extinction thresholds

We tested universal extinction thresholds across all plankton groups as an alternative to our size-based approach to evaluate the robustness of our results. In these sensitivity tests, we applied constant biomass thresholds (1×10^{-9}, 1×10^{-10}, and 1×10^{-11} mmol C m^{-3}) uniformly across all plankton functional types, rather than scaling thresholds by organism size. The results demonstrate that these universal thresholds fail to reproduce the observed extinction selectivity patterns observed in the fossil record (Extended Data Fig. 6). While the size-based approach shows clear latitudinal gradients in survival with preferential preservation in high latitudes, the universal thresholds exhibit either minimal extinction at low thresholds (1×10^{-11} mmol C m^{-3}) or complete global extinction at high thresholds (1×10^{-9} mmol C m^{-3}). The intermediate threshold (1×10^{-10} mmol C m^{-3}) instead shows maximal survival in the subtropical gyres, which is not in line with fossil data. These results confirm that incorporating size-dependent metabolic constraints is essential for accurately modeling marine ecosystem responses to environmental perturbations.

Validation of cGENIE model results

General features of cGENIE ocean physics

The cGENIE ocean model relies on linear friction approximation and reduced spatial resolution to increase the computational efficiency. Despite these simplifications, the model reproduces key features of large-scale ocean circulation. For instance, cGENIE accurately captures the location of modern deep-water formation in the North Atlantic and Southern Ocean (Fig. S7). Similarly, the pre-industrial mixed layer depth of the model compares well with the ECCOv4 ocean state estimate (Fig. S6). Comparing the K-Pg model result with the fully prognostic model (MOM3) used in Brugger et al.[41] shows similarly pronounced changes of mixed layer depth in the high latitudes (Fig. S4). This agreement supports the robustness of cGENIE mixed layer response to K-Pg cooling (Fig. 2). Together with the validation of our Maastrichtian experiments below demonstrate cGENIE provides a reliable representation of ocean physics to investigate the K-Pg extinction event.

Maastrichtian model-data comparisons

After running the model for 10,000 years, the equilibrium simulation reaches a global mean temperature of 22.8 °C, consistent with recent data-assimilated reconstruction range (21.5 to 26.8 °C; 5-95 percentile interval)[89] for the Late Cretaceous. The modelled global mean ocean surface temperature is 26.4 °C, again in agreement with the proxy data[36,37] (Extended Data Fig. 1). Global organic and inorganic carbon export are 6.7 Pg C yr^{-1} and 1.8 Pg C yr^{-1}, respectively. The resulting dissolved inorganic carbon and total alkalinity of surface seawater are 1815 and 1946 μmol kg^{-1} respectively, close to the estimates from Zeebe and Tyrrell[90]. The model simulates deep-water formation in the North and South Pacific, consistent with the neodymium isotope evidence[91] and higher-resolution General Circulation Model outputs (Fig. S7). The global meridional overturning circulation (GMOC) stream function shows that the strong south-sourced deep water dominates the Maastrichtian deep ocean (Fig. S8, similar to the recent study using the CCSM4 model[92]). The vertical distribution of carbon isotopes is consistent with proxy data (Fig. S5). We also find nutrient trapping and anoxic conditions in the modelled Atlantic (Fig. S9), supported by the high Maastrichtian $\delta^{15}N$ observations that infer anaerobic denitrification[93]. For the plankton ecosystem, the simulated symbiosis trait of planktic foraminifera in the Late Cretaceous is similar to what found in modern (Fig. S10): symbiotic foraminifera prefer the low-latitude oligotrophic environment, while non-symbiotic foraminifera dominated other regions. This is supported by several references[77,94,95] that suggested Late Cretaceous symbiotic taxa *Pseudoguembelina*, *Racemiguembelina*, and *Planoglobulina acervulinoides* were generally limited to tropical and subtropical open oceans. The trait distribution that more non-symbiotic generalists exist in the pre-impact high latitudes[96] also supports our interpretation that light loss caused the selective extinction and preferentially kill the taxa relying on photosymbiosis. Overall, these validations collectively demonstrate the model's strong capacity in simulating Late Cretaceous climate, ocean circulation, biogeochemistry and plankton ecology.

K-Pg model-data comparisons

Across the K-Pg boundary, the marine plankton ecosystem experienced pronounced selective extinction[97]. Planktic foraminifera and calcareous nannoplankton with excellent fossil records show the highest global extinction rates. Planktic foraminifera are reported to have 95% extinction rate[30], with only small and opportunistic *Guembelitria cretacea* and *Guembelitria blowi* survived the extinction. Other taxa like *Muricohedbergella* and *Heterohelix* sometimes have also been recognised as potential survivors, although this remains debated[98]. Calcareous nannoplankton similarly have 91% (120 out of 131) species extinction rate[99]. Bown et al.[15] showed that the calcareous nannoplankton survivors (e.g., *Biscutum* and *Zeugrhabdotus*) were mostly small and adapted to eutrophic and neritic environments. The similar neritic ecology exists in planktic foraminifera survivors and non-calcifying haptophytes which did not experience major extinction[100]. Jiang et al.[7] also reported that some moderately calcified taxa *Cyclagelosphaera* also survived the extinction, suggesting a secondary impact of ocean acidification. Overall, both key plankton calcifying groups show nearly complete loss of abundance and calcite production across the boundary. These features are well captured by the model, which simulates functional richness and suggests complete functional extinction of foraminifera and survival of only one nannoplankton type (Fig. 1), consistent with the extremely low $CaCO_3$ accumulation rate in the lowest Danian sediments[13]. The slight overestimation of extinction in the model can be attributed to two main limitations: the model does not represent (1) the neritic feeding ecology of plankton and the complex coastal dynamics; and (2) the potential speciation mechanism that some planktic foraminifera evolved from benthic ancestors after the extinction[101,102].

Another important feature of plankton extinction selectivity is its strong latitude dependence. Overall, high-latitude marine ecosystems are less severely impacted than low-latitude ecosystems. This pattern seems to be widespread including marine invertebrates, dinoflagellates, diatoms, calcareous nannoplankton[7,103], and benthic foraminifera (as reviewed in Alegret and Thomas[104]). Planktic foraminifera extinction has also been suggested to be largely restricted to tropical and subtropical species[105]. However, the Antarctic sediment cores (ODP Site 689/690 on Maud Rise, Weddell Sea) are intensively reworked (Huber, 1990 and Scott et al. 1990 in the ref[106]), hampering us to give a firm conclusion for high-latitude planktic foraminifera survival. Nonetheless, pre-extinction high-latitude assemblages were dominated by non-symbiotic opportunist taxa such as *Guembelitria* (Keller and Pardo[96]). Such pre-existing trait distribution explains the reduced extinction selectivity at high latitudes and is consistent with our hypothesis of light limitation, rather than reflecting an artifact of sparse fossil records and low local trait diversity at high latitudes. Overall, the model reproduces this latitudinal selectivity in marine ecosystem extinction (Fig. 1), with the notable exception that some large phytoplankton (diatoms and dinoflagellates) survived in the model. This mismatch is discussed in the main text and is likely because of the dormancy strategy, which the model does not represent. Modern laboratory and field experiments show these taxa can enter resting stages for up to a century[51], far beyond the length of darkness period shown in the model.

The model also agrees with several other features of the post-K-Pg fossil data. For example, the success of mixotroph nannoplankton is well documented in fossil tests[4]. Our model likewise shows consistently high mixotroph biomass after the boundary. The bloom of "disaster acmes"[107,108] and the loss of planktic foraminifera's symbiosis[21] suggest that the post-K-Pg environments were eutrophic, as seen in our model results (Fig. 2). In addition, the model reproduces the proliferation of picophytoplankton observed in the fossil record after the K-Pg boundary. For example, Bralower et al.[5] analysed global micrite layers deposited after the boundary and found calcite crystals highly similar to those precipitated by modern cyanobacteria. Biomarker studies (e.g., Sepúlveda et al.[42], Schaefer et al.[44]) also indicate rapid recovery of non-calcareous bacterial production after the impact. In our model, the success of these small phytoplankton is linked to their smaller body size and corresponding lower metabolic demand. Overall, despite some mismatches due to the limited model complexity, the model reproduces many of the key features observed in the fossil records. Because additional fossilised groups like molluscs and benthic foraminifera are not explicitly represented, further direct comparisons for these taxa are not currently possible.

Supplementary Figures

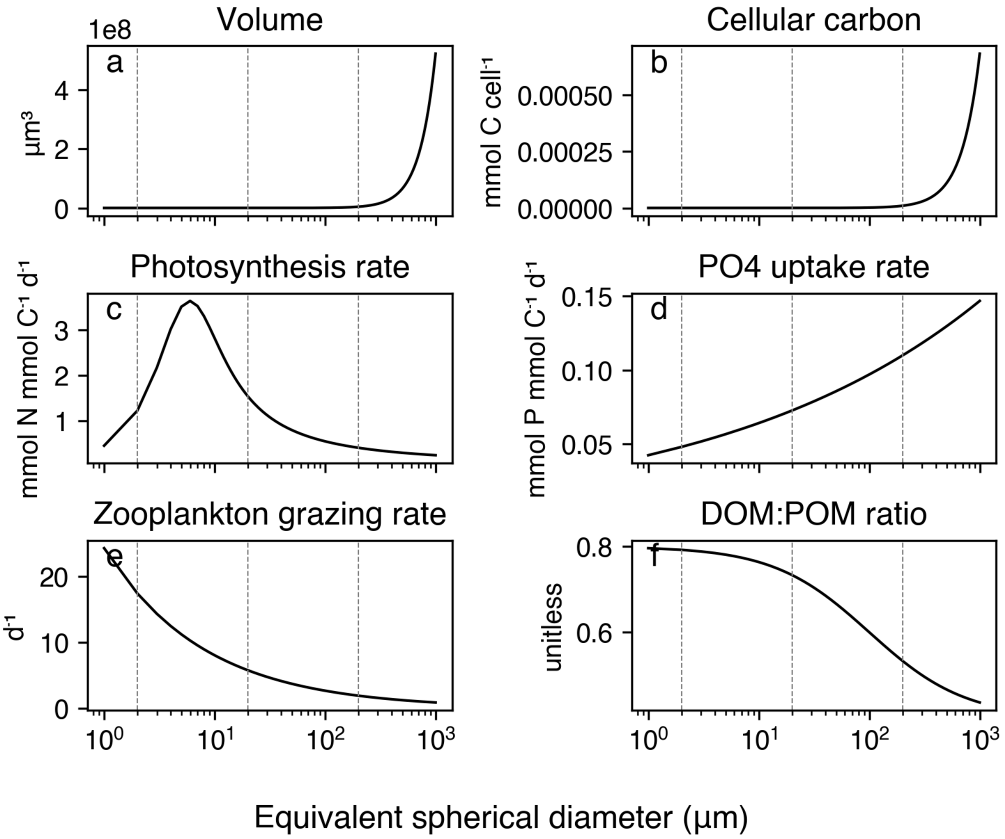

Fig. S1. The trait-based framework in EcoGENIE model. The body size set up in the model is used to calculate plankton volume (a) and other physiological traits, including (b) carbon quota, (c) maximal photosynthesis rate, (d) maximal PO_4 uptake rate, (e) maximal zooplankton grazing rate, and (f) exported ratio between dissolved organic matter and particular organic matter. The x axis is shown in log scale. The grey lines at 2, 20, and 200 µm are set marked to categorise picoplankton, nanoplankton, microplankton, and mesoplankton.

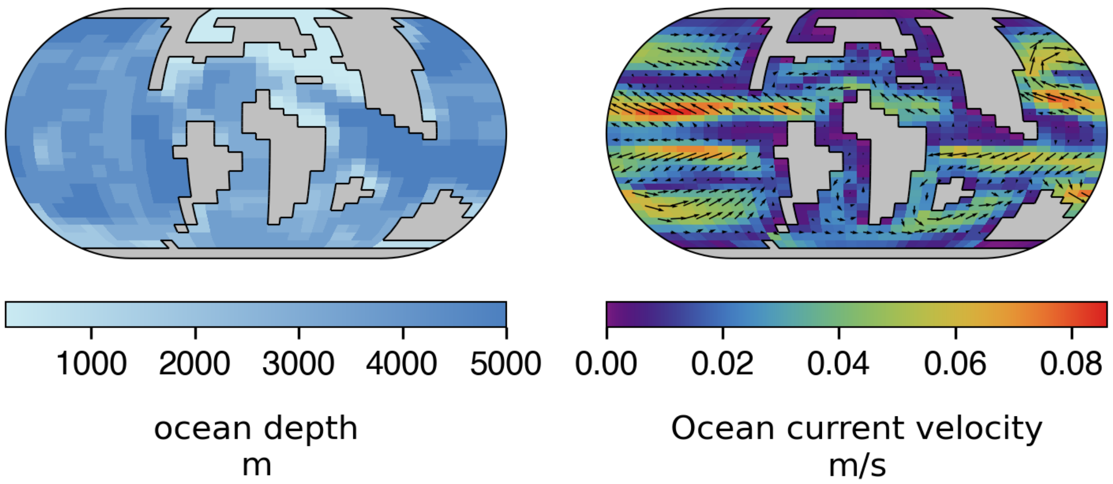

Fig. S2. **The cGENIE Late Cretaceous model configuration**. (a) Continental configuration and bathymetry and (b) Surface ocean current velocity. Note the model does not include an Antarctic Circumpolar Current in the spin-up simulation because of the shallow bathymetries in the Drake Passage and Tasman Seaway.

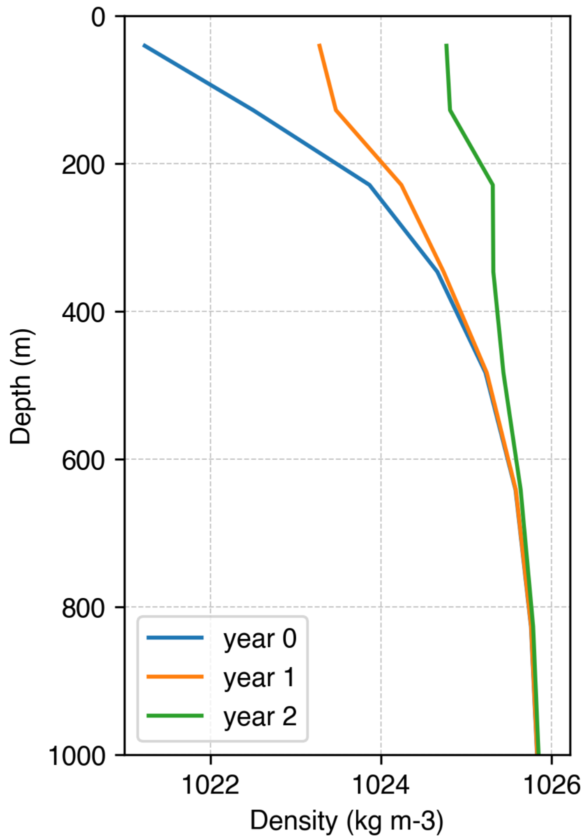

Fig. S3. Globally averaged vertical seawater density gradient. Year 0: Pre-impact control run; Years 1 and 2 represent the first and second year after applying the K-Pg climate forcings. The K-Pg impact strongly increases ocean density in the top 1,000 meters as indicated by the change away from the blue line.

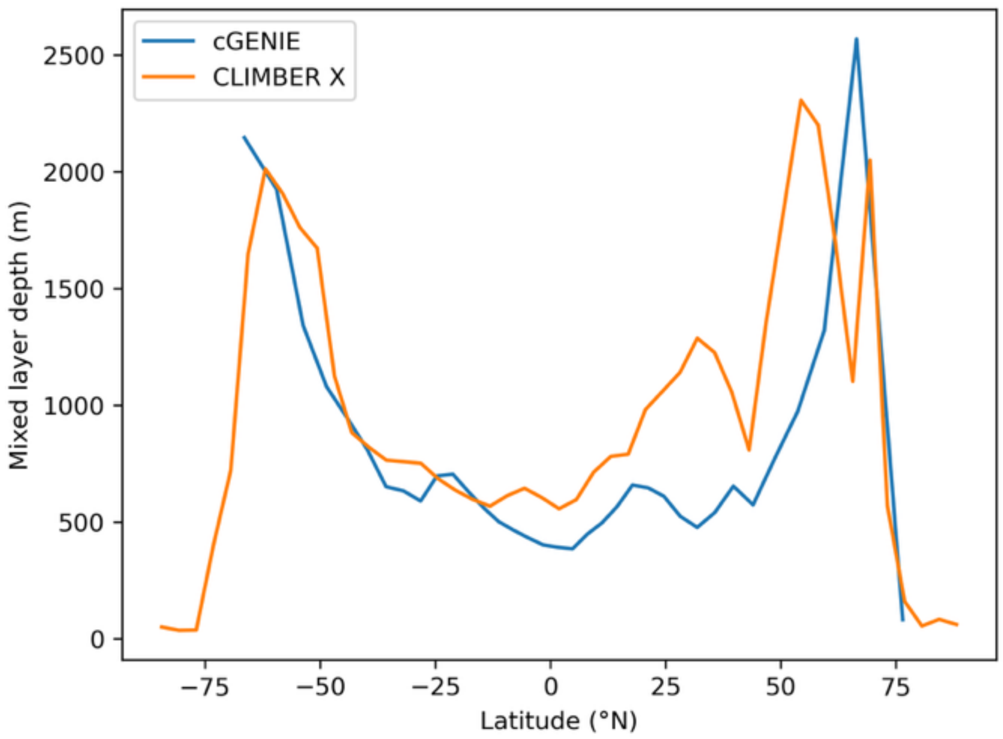

Fig. S4. Mixed layer depth in the second year after the K-Pg boundary. The cGENIE model with a simple ocean physics compares well with CLIMBER-3+C's MOM3 ocean model[24].

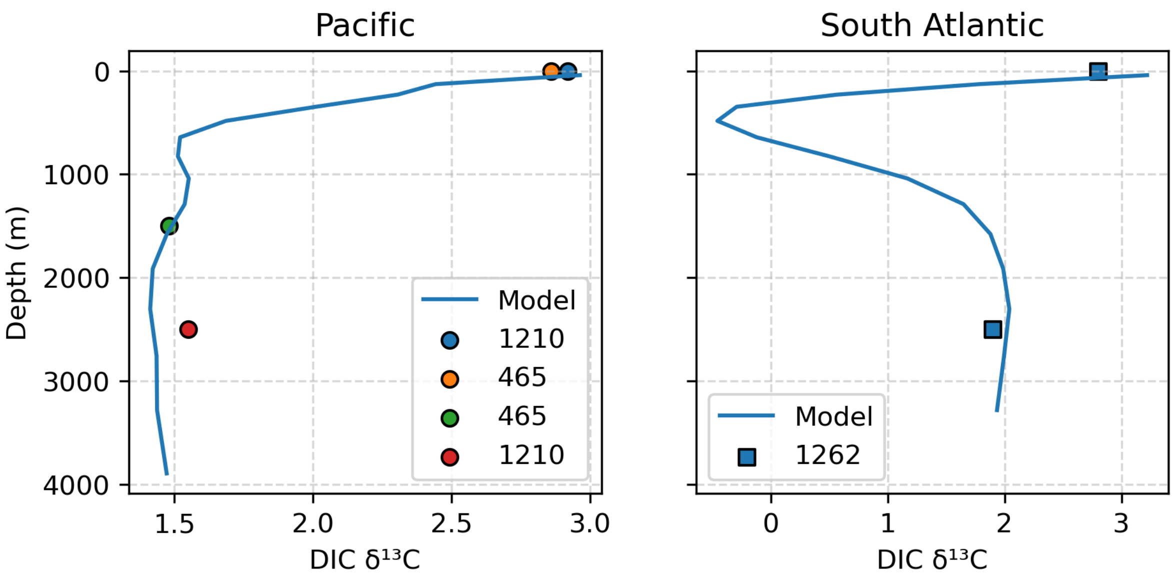

Fig. S5. Simulated carbon isotopes in the Late Cretaceous compared to proxy data. Pacific data from Alegret et al.[68] (surface: bulk $\delta^{13}C$; deep: benthic foraminifera $\delta^{13}C$) and Atlantic data from Birch et al.[11] (surface: averaged planktic foraminifera $\delta^{13}C$; deep: benthic foraminifera $\delta^{13}C$).

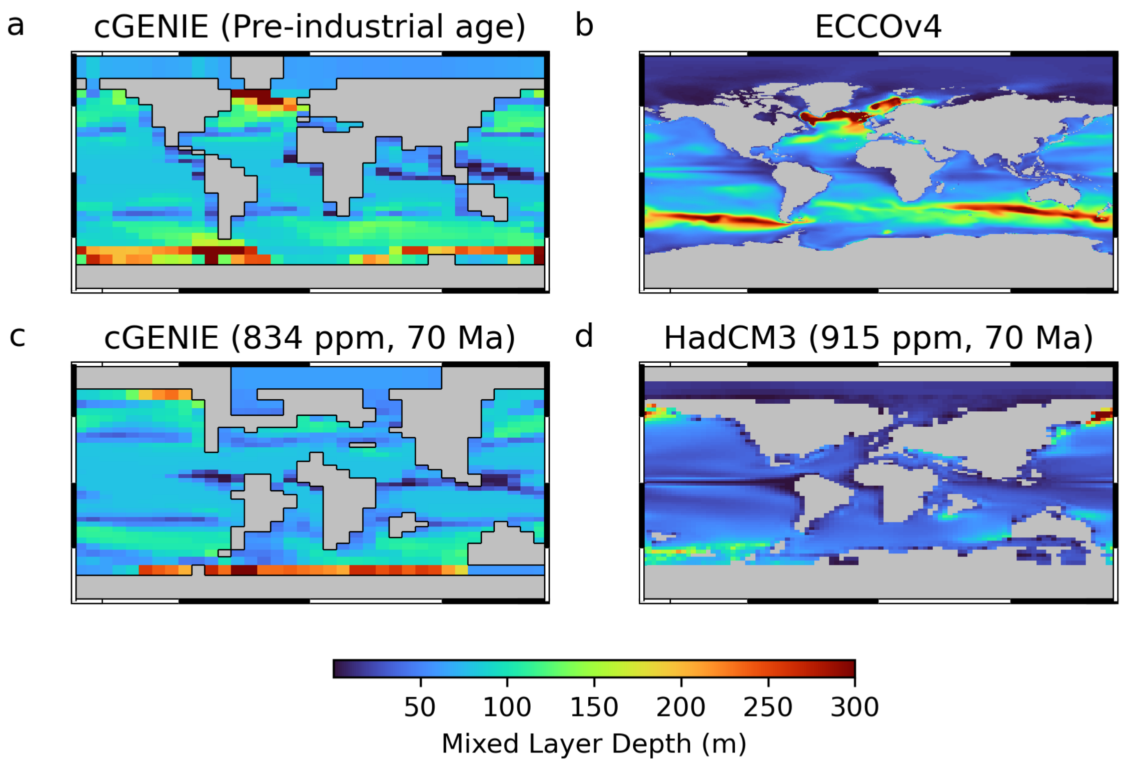

Fig. S6. Marine mixed layer depth in modern and Late Cretaceous. (a-b) The cGENIE model output compares well to the ECCOv4 climatology (1992-2017); (c-d) and the cGENIE model generates a reasonable agreement with HadCM3 model (scotese_07 series in Judd et al.[67]), despite using different Maastrichtian CO_2 given the proxy uncertainty .

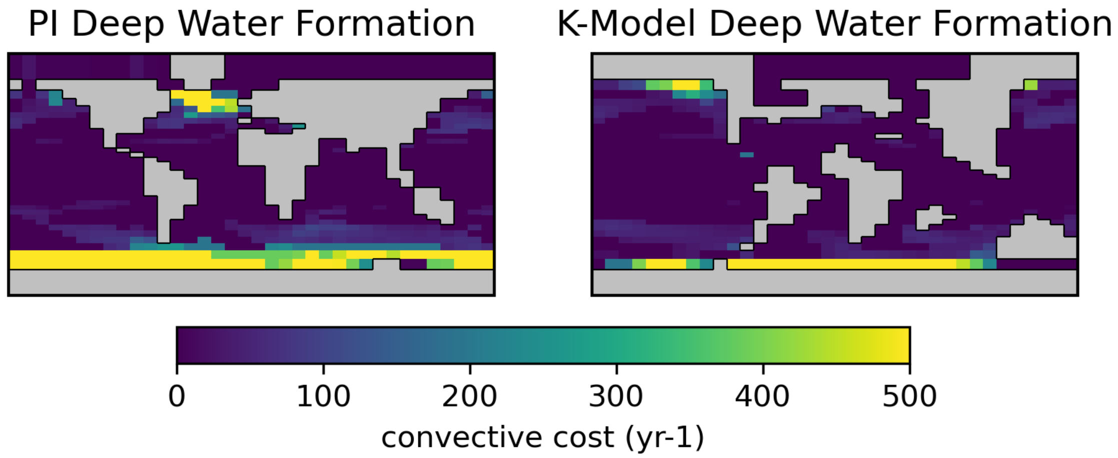

Fig. S7. The locations of deep-water formation in the modern and Late Cretaceous. In the pre-industrial model configuration, regions of high convective cost correspond to deep water formation in the North Atlantic and Southern Ocean, specifically the North Atlantic Deep Water and Antarctic Bottom Water. In contrast, the Late Cretaceous model suggests North Pacific and Southern Ocean as source of deep water in agreement with isotopic evidence[39,66].

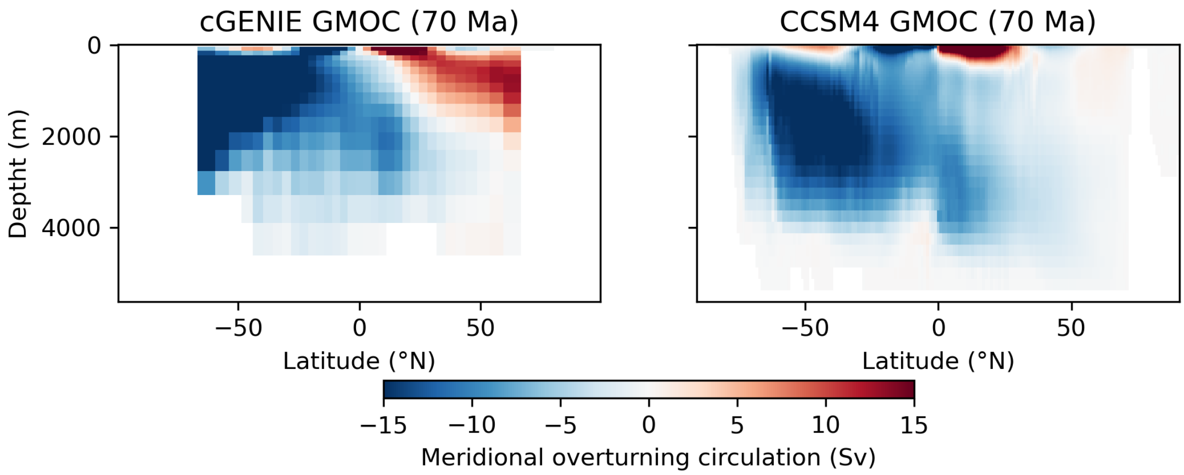

Fig. S8. Global meridional overturning stream function in the Late Cretaceous. (left) cGENIE; (right) CCSM4 model with 4×CO_2 (1112 ppm) from Ladant et al.[40].

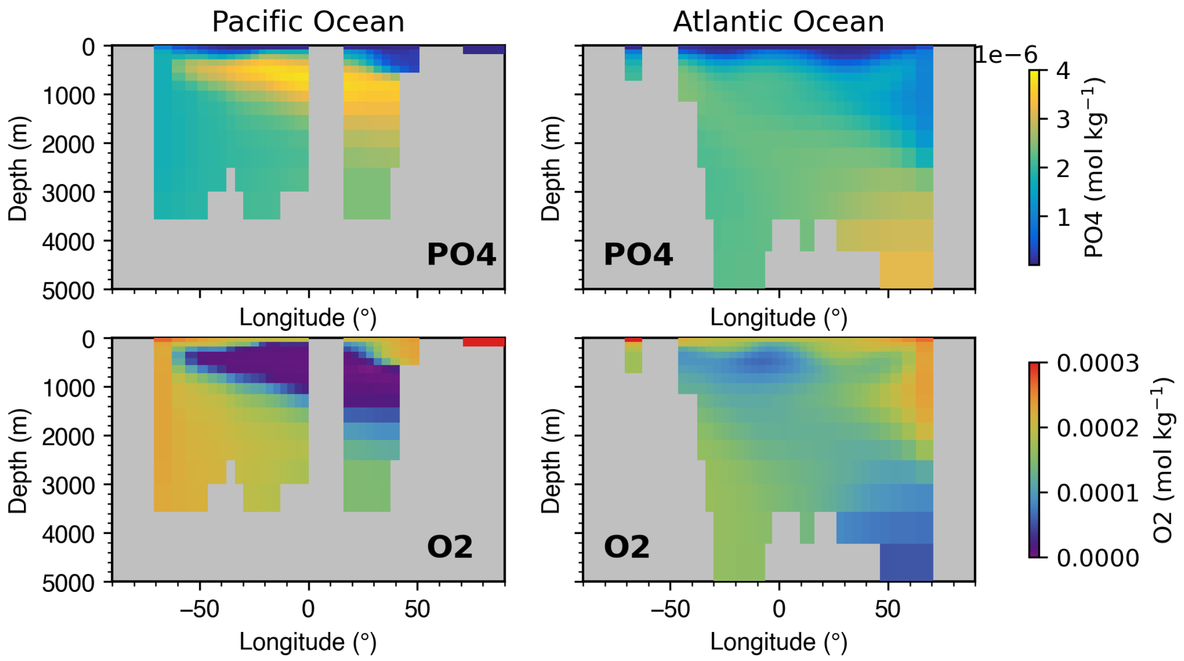

Fig. S9 Vertical transects of dissolved oxygen and phosphate concentrations along meridional sections in the Atlantic Ocean (5°W) and Pacific Ocean (175°E) in the Late Cretaceous equilibrium simulations.

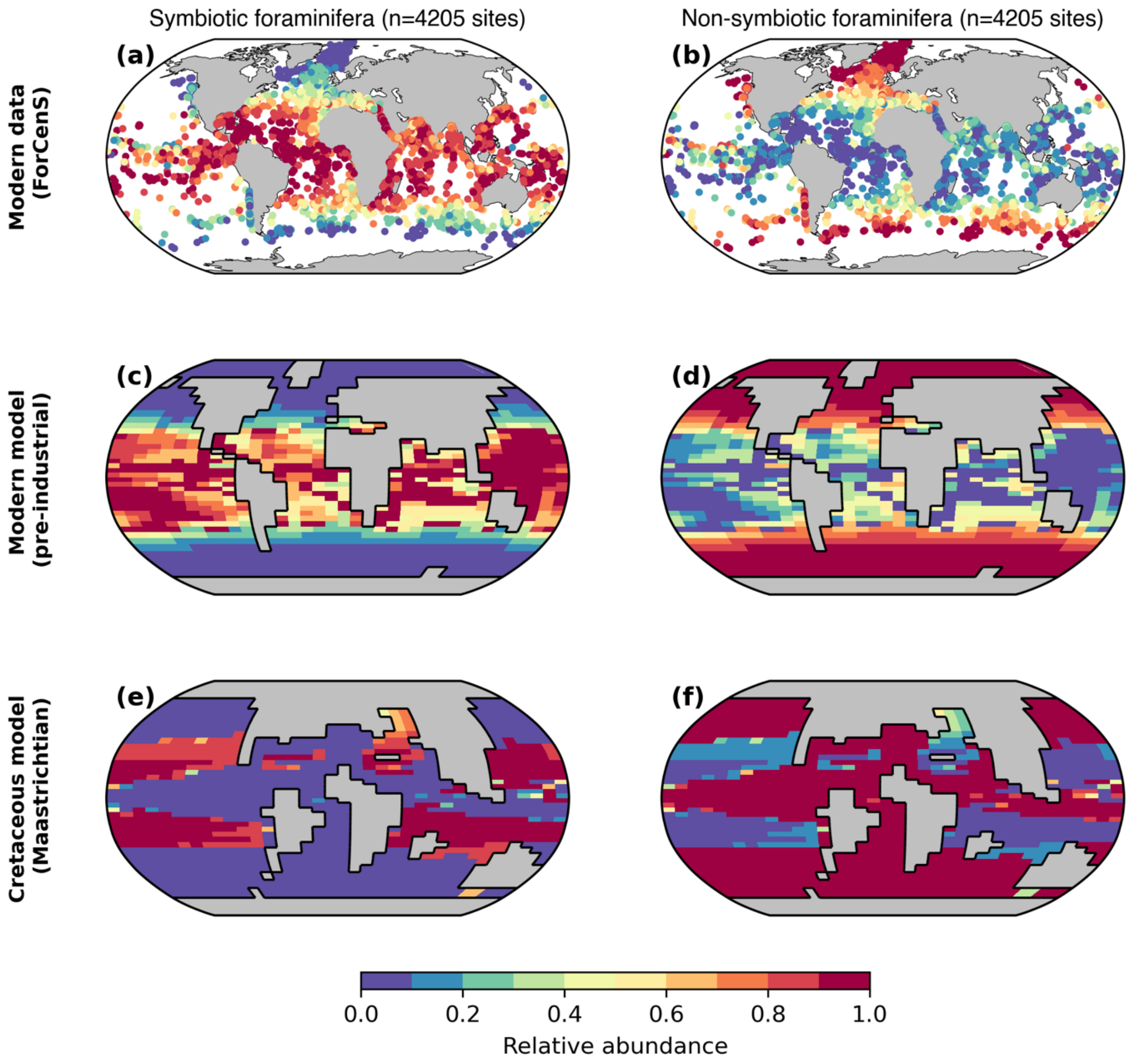

Fig. S10 The simulated Late Cretaceous symbiosis trait distribution for planktic foraminifera, compared with modern observations and model. (a, b) Modern observed relative abundance of symbiotic and non-symbiotic foraminifera from the ForCenS database. (c, d) Pre-industrial model simulation using EcoGENIE. (e, f) Late Cretaceous (Maastrichtian) model using the same trait-based framework. The modern symbiotic and non-symbiotic foraminifera include both spinose and non-spinose functional types, while the Late Cretaceous only has non-spinose functional types. Consistent with modern observations, symbiotic foraminifera are concentrated at low-latitude open-ocean in both model configurations, while non-symbiotic foraminifera dominate at high latitudes.

Table S1. The size range of seeded plankton functional groups in K-Pg experiment

	Minimum size	Maximum size	No.
Phytoplankton	0.1	1224.0	32
Zooplankton	0.1	1224.0	32
Mixotroph	0.1	1224.0	32
Symbiont-barren foraminifera	40.4	330.4	8
Symbiont-bearing foraminifera	100.0	874.2	8

71. Edgar, K. M. *et al.* Reviews and syntheses: a trait-based approach to constrain controls on planktic foraminiferal ecology – key trade-offs and current knowledge gaps. *Biogeosciences* **22**, 3463–3483 (2025).

72. Droop, M. R. Vitamin B12 and Marine Ecology. IV. The Kinetics of Uptake, Growth and Inhibition in Monochrysis Lutheri. *J. Mar. Biol. Ass.* **48**, 689–733 (1968).

73. Burd, A. B. Modeling the Vertical Flux of Organic Carbon in the Global Ocean. *Annual Review of Marine Science* **16**, 135–161 (2024).

74. Ward, B. A. & Follows, M. J. Marine mixotrophy increases trophic transfer efficiency, mean organism size, and vertical carbon flux. *Proc Natl Acad Sci USA* **113**, 2958–2963 (2016).

75. Reinhard, C. T. *et al.* The impact of marine nutrient abundance on early eukaryotic ecosystems. *Geobiology* **18**, 139–151 (2020).

76. Birch, H. S., Coxall, H. K., Pearson, P. N., Kroon, D. & Schmidt, D. N. Partial collapse of the marine carbon pump after the Cretaceous-Paleogene boundary. *Geology* **44**, 287–290 (2016).

77. Abramovich, S., Keller, G., Stüben, D. & Berner, Z. Characterization of late campanian and maastrichtian planktonic foraminiferal depth habitats and vital activities based on stable isotopes. *Palaeogeogr. Palaeoclimatol. Palaeoecol.* **202**, 1–29 (2003).

78. Cao, L. *et al.* The role of ocean transport in the uptake of anthropogenic CO_2. *Biogeosciences* **6**, 375–390 (2009).

79. Panchuk, K., Ridgwell, A. & Kump, L. R. Sedimentary response to Paleocene-Eocene Thermal Maximum carbon release: A model-data comparison. *Geology* **36**, 315–318 (2008).

80. Naidoo-Bagwell, A. A. *et al.* A diatom extension to the cGEnIE Earth system model – EcoGEnIE 1.1. *Geoscientific Model Development* **17**, 1729–1748 (2024).

81. Tagliabue, A. *et al.* How well do global ocean biogeochemistry models simulate dissolved iron distributions?: GLOBAL IRON MODELS. *Global Biogeochem. Cycles* **30**, 149–174 (2016).
82. During, M. A. D. *et al.* The Mesozoic terminated in boreal spring. *Nature* **603**, 91–94 (2022).
83. MacLeod, K. G. *et al.* Isotopic evidence from a brazos river (texas, USA) cretaceous/paleogene boundary section consistent with a pulse of greenhouse warming shortly after the chicxulub impact. *Global Planet. Change* **253**, 104924 (2025).
84. Artemieva, N., Morgan, J. & Party, E. 364 S. Quantifying the Release of Climate-Active Gases by Large Meteorite Impacts With a Case Study of Chicxulub. *Geophysical Research Letters* **44**, 10,180-10,188 (2017).
85. Vellekoop, J. *et al.* Shelf hypoxia in response to global warming after the cretaceous-paleogene boundary impact. *Geology* **46**, 683–686 (2018).
86. Kajiwara, Y. & Kaiho, K. Oceanic anoxia at the cretaceous/tertiary boundary supported by the sulfur isotopic record. *Palaeogeogr. Palaeoclimatol. Palaeoecol.* **99**, 151–162 (1992).
87. Rodiouchkina, K. *et al.* Reduced contribution of sulfur to the mass extinction associated with the chicxulub impact event. https://eartharxiv.org/repository/view/7548/ (2024).
88. Witts, J. D. *et al.* The impact of the cretaceous–paleogene (K–pg) mass extinction event on the global sulfur cycle: evidence from seymour island, antarctica. *Geochim. Cosmochim. Acta* **230**, 17–45 (2018).
89. Judd, E. J. *et al.* The PhanSST global database of Phanerozoic sea surface temperature proxy data. *Sci Data* **9**, 753 (2022).

90. Zeebe, R. E. & Tyrrell, T. History of carbonate ion concentration over the last 100 million years II: revised calculations and new data. *Geochim. Cosmochim. Acta* **257**, 373–392 (2019).

91. Thomas, D. J., Korty, R., Huber, M., Schubert, J. A. & Haines, B. Nd isotopic structure of the Pacific Ocean 70–30 ma and numerical evidence for vigorous ocean circulation and ocean heat transport in a greenhouse world. *Paleoceanography* **29**, 454–469 (2014).

92. Ladant, J.-B. *et al.* Paleogeographic controls on the evolution of late cretaceous ocean circulation. *Clim. Past* **16**, 973–1006 (2020).

93. Du, Y. *et al.* Tectonic controls on nitrogen cycling and ocean ventilation dynamics in the late cretaceous equatorial atlantic. *Earth Planet. Sci. Lett.* **667**, 119517 (2025).

94. D'Hondt, S. & Zachos, J. C. Cretaceous Foraminifera and the Evolutionary History of Planktic Photosymbiosis. *Paleobiology* **24**, 512–523 (1998).

95. Houston, R. M. & T. Huber, B. Evidence of photosymbiosis in fossil taxa? Ontogenetic stable isotope trends in some Late Cretaceous planktonic foraminifera. *Marine Micropaleontology* **34**, 29–46 (1998).

96. Keller, G. & Pardo, A. Disaster opportunists Guembelitrinidae: index for environmental catastrophes. *Marine Micropaleontology* **53**, 83–116 (2004).

97. Macleod, N. *et al.* The cretaceous-tertiary biotic transition. *J. Geol. Soc. London.* **154**, 265–292 (1997).

98. Arenillas, I., Arz, J. A., Metsana-Oussaid, F., Gilabert, V. & Belhai, D. Hypothesis testing on the planktic foraminiferal survival model after the KPB mass extinction: evidence from Tunisia and Algeria. *Fossil Rec.* **25**, 43–63 (2022).

99. Gibbs, S. J. *et al.* Scaled biotic disruption during early Eocene global warming events. *Biogeosciences* **9**, 4679–4688 (2012).

100. Medlin, L. K., Sáez, A. G. & Young, J. R. A molecular clock for coccolithophores and implications for selectivity of phytoplankton extinctions across the K/T boundary. *Mar. Micropaleontol.* **67**, 69–86 (2008).

101. Morard, R. *et al.* Renewal of planktonic foraminifera diversity after the Cretaceous Paleogene mass extinction by benthic colonizers. *Nat Commun* **13**, 7135 (2022).

102. Darling, K. F. *et al.* Surviving mass extinction by bridging the benthic/planktic divide. *Proceedings of the National Academy of Sciences* **106**, 12629–12633 (2009).

103. Schueth, J. D., Bralower, T. J., Jiang, S. & Patzkowsky, M. E. The role of regional survivor incumbency in the evolutionary recovery of calcareous nannoplankton from the Cretaceous/Paleogene (K/Pg) mass extinction. *Paleobiology* **41**, 661–679 (2015).

104. Alegret, L. & Thomas, E. Benthic foraminifera across the cretaceous/paleogene boundary in the southern ocean (ODP site 690): diversity, food and carbonate saturation. *Mar. Micropaleontol.* **105**, 40–51 (2013).

105. Keller, G. The end-cretaceous mass extinction in the marine realm: year 2000 assessment. *Planet. Space Sci.* **49**, 817–830 (2001).

106. *Proceedings of the Ocean Drilling Program, 113 Scientific Reports*. vol. 113 (Ocean Drilling Program, 1990).

107. Jones, H. L., Lowery, C. M. & Bralower, T. J. Delayed calcareous nannoplankton boom-bust successions in the earliest Paleocene Chicxulub (Mexico) impact crater. *Geology* **47**, 753–756 (2019).

108. Alvarez, S. A. *et al*. Diversity decoupled from ecosystem function and resilience during mass extinction recovery. *Nature* **574**, 242–245 (2019).
