# Supplementary material for: Darkness and body size shaped end-Cretaceous marine extinction patterns
Source: Nature. 2026 May 27;655(8124):957–62. doi: 10.1038/s41586-026-10541-4 (PMC13391373; doi:10.1038/s41586-026-10541-4)
Supplement: Supplementary file 3 — Peer Review File [file 41586_2026_10541_MOESM3_ESM.pdf]

Peer Review File

Darkness and body size shaped End-Cretaceous marine extinction patterns

Corresponding Author: Dr Rui Ying

Any redactions in this file are there to maintain patient confidentiality, the confidentiality of unpublished data, or to remove third-party material.

This file contains all reviewer reports in order by version, followed by all author rebuttals in order by version.

Version 0:

Reviewer comments:

Referee #1

(Remarks to the Author)
Dear Dr. Gee,

Thank you for the opportunity to review this interesting manuscript by Ying et al. It addresses the important and long-standing question of the physiological drivers of K/Pg mass extinction in the marine realm. This question of extinction mechanism has been central to K/Pg studies since the impact hypothesis was first put forward, with productivity collapse, ocean acidification, metal toxicity, etc. all considered as primary culprits at different points with no clear answer emerging. The authors used a trait-based ecosystem model combined with independent constraints on impact-induced environmental changes to assess the roles of these different environmental changes on a model plankton ecosystem. This model successfully reproduced geographic and ecological patterns of extinction and indicated that light loss was the key environmental change driving those patterns, with picoplankton and mixotrophs better adapted to a lower energy environment, as well as polar species, which are already adapted to months of darkness every year. This ecosystem modelling is an interesting and clever way to try to understand extinction mechanisms and is a welcome step forward in our understanding of these processes at the K/Pg. However, it is not the last word on this problem because (as far as I can tell) it does not include a very important parameter, namely depth habitat.

Water column structure, and the depth habitats that it creates, has long been known to be important for plankton diversity (Hart, 1980, Cretaceous Research; Leckie et al., 2002, Paleoceanography; Bown et al., 2004, Calcispheres [book]; Ezard et al., 2011 Science; Fraass et al., 2015 Annual Reviews, Lowery et al., 2020 Annual Reviews). Other extinctions in the plankton have been tied to changes in water column structure changes, like Cretaceous OAEs (Leckie, 1989, Palaeo3; Erbacher and Thurow, 1997 Marine Micropaleo; Premoli Silva and Sliter, 1999, GSA Special Pub 332; Leckie et al., 2002, Paleoceanography; Watkins et al., 2005, Paleoceanography; Huber and Leckie, 2011, JFR); see discussion and additional citations in Lowery et al., 2020, Annual Reviews (a paper I bring up not because I expect you to cite it, to be clear! It just goes into way more discussion and citations of this than would be reasonable to include in a review here). One of the most eye-popping results of global climate model simulations of the Chicxulub impact is the huge expansion of the global mixed layer (>1000 m water depth in Brugger et al., for example) as a result of the severe cooling and the massive storms that would have spun up as a result. This destruction of thermal stratification would have obliterated all the sub-mixed layer depth habitat niches occupied by tropical to temperate plankton. Less extreme changes in water column stratification have been associated with foram, nanno, and radiolarian extinctions at other points in Earth history.

The greatest diversity of calcareous plankton is in the tropics, while the greatest diversity of siliceous plankton is near the poles. It is possible, as suggested in this manuscript, that the preferential extinction of the former at the K/Pg is due to the changes in energy availability during the impact winter as the usually sunny tropical to temperate waters went dark, while the latter survived because they were adapted to polar darkness, BUT it is also possible that the preferential extinction of calcareous plankton was due to the destruction of their depth habitat as tropical/subtropical water column stratification was wiped away by cooling (this is supported by the fact that the main survivor species of planktic foraminifera was a neritic taxon, adapted to the unstratified waters of the continental shelf) while the survival of many siliceous plankton is because they lived in polar regions that already lack a thermocline (or a strong, permanent one anyway) and thus they didn't have

depth niches to lose. I tend to think that it's both, that the reason calcareous plankton had such high extinction rates is because they were dealing with the combo-punch of a reduced energy environment from global darkness AND the elimination of their preferred depth habitats. If it were only the depth habitats destroyed, then this may have played out more like Cretaceous OAEs, with only the deeper dwelling forms going extinct, while if it was only darkness then the deeper dwelling forms of zooplankton may have made it through (relatedly, a question I had reading this is how might deeper-dwelling zooplankton have responded to a few years of darkness).

As it is, I did not see anything in the model methods about depth habitat, and because depth habitat is so important to plankton diversity I think that this *must* be addressed to bolster the conclusion that global darkness was the key factor in the extinctions. I don't think this necessarily requires an entirely new model and run of experiments, and the ability of these results to reproduce the geographic patterns is compelling on its face, but I do think that depth habitat, and its implications for the results presented here, needs to be discussed in detail.
The only other major point I want to bring up here is that the planktic foram functional groups in the model are spinose symbiont-bearing and non-spinose non-symbiont bearing. The former didn't exist in the Cretaceous (spines wouldn't evolve until the Danian and all Cretaceous photosymbiont-bearing forams were non-spinose). This should be addressed and justified in the methods. Other minor points are raised by line number below. I am not a modeler, and thus cannot assess the methods related to model construction or the code.

I think this is an interesting and provocative paper that deserves to be published in a journal like Nature, but at the moment I think it has a pretty large blind spot (depth habitat) and I think it will be much more impactful if the points raised above were incorporated into the text before it is published. I would be happy to read a revised version.

Best,
Chris Lowery
University of Texas

Line 1 - A suggestion on the title – a shorter and more easily understood way to say "light loss" would simply be "darkness" (Also, in my opinion, "darkness" is more evocative)

Line 15 – a pedantic point here but it's 75% of species in the *fossil* record.

Line 31 – another pedantic point: the Yucatán wasn't a peninsula when the asteroid hit, it was a carbonate platform.

Line 32 – Oh boy 3 pedantic points in a row, I'm sorry about this: Alvarez et al. didn't know anything about the location of the crater so you should cite another paper here too (Schulte et al., 2010, Science, is the classic, but if you're pressed for references you could cite Morgan et al. here, too)

Line 35 – the target rocks was not gypsum (CaSO4 2H20), but anhydrite (CaSO4), as is well documented from boreholes across the Yucatan. See Brett et al. 1992, for example. https://www.sciencedirect.com/science/article/pii/0016703792904069

Line 40 – there might be something screwy with your references here, as refs 10-12 are papers about the Ir layer, and network analysis, not extinction in the groups listed in this sentence. Schulte et al. 2010 is a good reference for ammonites and rudists if you don't want to cite all the underlying studies. Sibert and Norris, 2015 (PNAS) is good for sharks and fishes. For the plankton, you might use Fraass et al., 2015 (forams) Bown et al., 2005 (nannos).

Line 41-42 – I'm not sure these are the best references for this statement. Thomas et al. 2007 (GSA Special Paper 424) and/or Culver, 2003 (Marine Micropaleontology), are better references for benthic foram survivorship than MacLeod.

Line 47 – the role of ocean acidification at the K/Pg boundary has been discussed as far back as Prinn and Fegley, 1987 (EPSL) MacDougall, 1988 (Science)

Line 49 – I'm not sure if the original K/Pg impact hypothesis put forward by Alvarez et al. should be described as an "alternative hypothesis." I'd probably switch this sentence with the OA sentence, so the impact winter is first and OA is the "alternative"

Line 51-52 – why couldn't productivity collapse explain the observed geographic patterns? Different parts of the ocean have different amounts of primary production, with different seasonalities, supporting ecosystems that are adapted to different amounts and types of food. Why couldn't have those ecosystems responded in geographically distinct ways to a reduction in primary production?

Line 53 – limited AND geographically heterogeneous (see Hull and Norris 2011, for example). Again, I don't think you can write off productivity changes so quickly, and I think this needs a bit more discussion.

Line 67-68 – why are foraminifera separate from zooplankton?

Line 82-84 – did plankton have specific depth habitats, as in the real ocean? This would be very important to mention because of the subsequent changes in ocean structure due to impact-induced cooling.

Line 83-84 – is this similar to diversity trends in the Cretaceous ocean, though? It was a fundamentally different global ocean

than our current icehouse world and we know that, for example, the modern latitudinal diversity gradient only emerged 15 million years ago (Fenton et al., 2023 Nature).

Line 85 – typo ("event")

Line 86 – "late" should be capitalized, as the Late Cretaceous is a formal Epoch.

Line 87 – I think this 700 ppm CO_2 pulse needs further explanation. Is this meant to recreate the 0.3 pH unit change observed across the K/Pg boundary? And, if so, does making the whole pulse out of CO_2 (rather than sulfate and nitrate) get the timing right? Does it reproduce the observed pH change in the model? The timing seems very important here if you're evaluating OA as an extinction mechanism, as quicker acidification is going to be more damaging to pelagic calcifiers. I see you partially address this further down (Line 195) but I think it would be worth mentioning up here too, so you don't have people like me wondering about it for 100 more lines.

Line 126-128 – just like the real post-K/Pg ocean, you should point out! Also, what does the change in plankton size do to export flux, and to nutrient cycling in the euphotic zone? Did a shift to smaller plankton keep more nutrients in the upper ocean?

Line 155-158 – but no one thinks CO_2, specifically, was what caused acidification, it was sulfate and nitrate aerosols converted into sulfuric and nitric acid (in addition to carbonic acid from the CO_2). Does your model include anything about the other, more important climate active gasses? (and if not, you should explain how that impacts your interpretations in the text).

Line 178 – this is in line with predictions by Henehan et al. (2016) and observations by Lowery and Bralower (2022)

Line 172-175 – I think this is exactly right. The drop in productivity was severe but not long enough to filter down to the deeper depths of the ocean.

Line 219 – does this post-K/Pg elevated biomass and diversity in the polar oceans in the model match observations (e.g., from Seymour Island?)

Line 235 – what is "neritic feeding" and how is it different than the grazing that non-photosymbiont-bearing forams do in the open ocean?

Line 301 – note that spines as a trait did not exist in the Cretaceous foraminifera. They evolved in the early Danian in Eoglobigerina. Cretaceous photosymbiont-bearing planktic forams were all non-spinose.

(Remarks on code availability)

Referee #2

(Remarks to the Author)
There seems to be building momentum for the "reduced solar insolation" theory to explain the actual mechanism of the KPg extinction based on geological evidence for fine dust particles (Senel et al, 2023) and based on the apparent mixotrophic nature of coccolithophores that emerge in the aftermath of the extinction (Gibbs et al., 2020). This paper goes one step further to use a trait based ecosystem model to try and explain the differential selectivity patterns of extinction due to either elevated CO_2, raised temperature or reduced solar insolation, coming to the conclusions to further build a picture of solar insolation being the primary driver.

Much as the science is interesting, I did find myself questioning quite what the model was actually doing here to offer something new to the table. It may have been the way the manuscript was written which always discussed "the model" without really saying what features within the model were of interest or were providing any kind of mechanistic insight or understanding. In many places, it struck me that pure logic and observations of the data could have provided the exact same end results. I could not help but feel sceptical with the model, that you put in different light sensitivity as a trait to different components of the ecosystem and the model then tells you that when you dial down insolation, you get differential extinction. The same is true of CO_2- how were the functional types described in relation to their CO_2 sensitivity, and so then is it any surprise that there is no differential impact of elevated CO_2? and to the temperature and to all the other features and how much more does the model tells you than what you prescribe as the traits. Much is made of the energy supply and demand and how that is dictating the differential extinction patterns in the abstract and summary- but the reader is never told how these are parameterised in the main text nor really can the reader follow the reasoning behind why this is so mechanistically important and quite what result the model generates to draw this conclusion The writing was incredibly opaque with regards to true insights revealed as opposed to testing a hypothesis of sensitivity when it was prescribed.

I also have a scepticism over the issue of adaptation of organisms within the model. Phytoplankton populations are typically very large and panmictic i.e. spread ubiquitously and so can generally adapt pretty quickly as new advantageous mutations exist somewhere on the planet, can be selected for easily and can then flourish. This is likely to be so of most small organisms with large population sizes including the cyanobactera. Typically the population sizes get smaller with larger organism size- which then means that adaptation is much slower. So to what extent could this provide an alternative

explanation to the differential extinction, but is not thrown up by the model as it is not put in as a parameter to test? I also wonder what role this could play in the ideas about low light adaptation and how fixed this may be? Perhaps there are oceanic fronts which may limit mixing within the population but again this is not discussed?

I shall make some comments on specific parts of the manuscript below:
Line 68 “To mimic extinction, we
69 also evoke a mechanism based on the minimal biomass required to maintain individuals'
70 metabolic processes by preventing plankton groups from photosynthesizing/grazing if their
71 biomass is lower than an individual's carbon content (Materials and Methods).”
Need to unpick this a bit more for the reader. It is very much not clear from the main text what parameters are being placed into the model that will allow a new insight into the extinction mechanism.

Line 75: “We first applied the model”. Both here and in the methods it is really hard to understand what is unique about the model, what parameters allow the derivation of the conclusions about differential extinction based on energy supply and demand. Nowhere is the construct of the energy supply and demand of cells outlined for the reader here and yet it is an integral part of the conclusion of this manuscript. This has to be outlined clearly so that it can be assessed. If it is as simple as small cells require less energy than large cells, then one does have to wonder quite what value the model truly offers here over pure logic.

Line 77: It appears that sea surface temperature and pH align with proxy data but what about estimates of deep ocean temperature and e.g. deep ocean oxygen content for example. It is important to try and validate the fields with as many apremeters as possible to give credence to the model output. What happens to deep ocean oxygen contents? With such a deep mixed layer depth seems that ocean convection potentially very limited with the atmosphere so presumably this could drive deep ocean O2 to incredibly low values so how did any aerobic organisms in the deep survive? And what about deep ocean CO32- concentrations. Can these be reconciled with sedimentary preservation of CaCO3. Ittwould be helpful to see a range of biogeochemical parameters that are produced via this modelled scenario to understand whether the outcomes are reasonable against the geological/sedimentary record. Certainly the changes to PO4 in surface waters seem pretty extreme.

Figure 1: What is the C isotope gradient doing- looks like it was very small pre-event with I think a positive to negative gradient of 1 ‰ through the water column (depending on whether the difference is the deep- the surface or the surface-the deep). The gradient then becomes very big (-2 ‰) post event so does not seem to mimic at all what is thought in the d13C proxies where the gradient in the Strangelove Ocean is eliminated due to a break down in the biotic pump.

Line 112: OK so there is differential extinction between the foraminifera, zooplankton and photosynthesisers but these statements really beg the question as to why? What is the model doing to realise these differential outcomes?

Line 122: So if there was a post extinction recovery bloom, is there any evidence for such a thing in the geological record? It may be simulated- and again makes total logical sense given a shift down in pumping of nutrients, there inevitably has to be a bloom once life is reinvigorated. But is there good geological evidence for this in the sediments that have generally been poured over for this interesting event?

Line 129 paragraph: Here “the model” seems to be able to do lots of things but yet again it feels like it is treated in the write up as a black box. There is no mechanistic interpretation in a direct way derived from the model. It just does lots of stuff.

Line 135: Why is there this differential extinction rate between the high latitude and low latitude populations?

Line 118: OK so there is a carbon excursion but the model does not simulate the collapse in the 13C gradient at all- it reverses it and makes it bigger than before?

How could adaptation alter the results. The phytoplankton functional types are rather fixed in traits but even on a 30 year timescale there is the potential for adaptation and selction? How would that alter the results?

Line 154/155 etc. The model produces these differential impacts with no differential impact of Co2 across the latitudes. Again- why is this so? Surely this is just dependent on how your parameterise the model according to these drivers? If the functional types are very sensitive to light differentially, then of course differential extinction selectivity will be displayed by forcing through reduction of solar radiation. It would be very good to explore quite where the model shows something different than would have been expected given the assumptions that were parameterised.

Line 165: So temperature did not have much of an impact- how was this parameterised in the model for the different functional types? And so can we mechanistically understand why this is the outcome of the model?

Line 191: No effects of acidification- again surely this depends on how this sensitivity is built into the trait assumptions?

Line 211: How is the model simulating this differential light loss across the latitudes? Is this associated with the distribution of dust from the impact? Or what are the assumptions driving this pattern?

Methods: Again there needs to be much more detail on the functional types and how the traits are prescribed. Selective extinction is a major part of the paper and it is not clear from what is written here how that extinction must work although it is

referred to in other papers. I think it should be reiterated here even if briefly to help the reader understand why there are these differential extinction patterns.

Line 215/216: The hypothesis about preadaptation to low light at the high latitude allowing for preferential survival rates, seems to go against some of the observed rapidity of adaptation typically within the phytoplankton communities given their generation time and typical mutation rates. How would the results of the model be different should it allow for adaptation within the lineages? How would adaptation alter the results of the model?
(e.g. https://aslopubs.onlinelibrary.wiley.com/doi/10.4319/lo.1998.43.4.0679 and many other papers by Geider)

Further, Surely deeper dwellers would also have preferentially survived potentially even in the lower latitudes as the event would have removed the competition from the surface waters. Low light adaptation is quite a plastic response in phytoplankton I believe.

Line 229: Some of the thinking in the literature about enhanced mixotrophy, as is invoked in the aftermath of the KPg extinction, is a greater ability to transfer biomass to higher trophic levels (https://royalsocietypublishing.org/doi/full/10.1098/rspb.2016.1755). These statements seem to be in contradiction of this thinking.

With a significant overhaul of the writing where the model does not just do “stuff” but there are consistent explanations throughout as to why the model produces the results that it does then it could provide a link between a mechanism of reduced solar irradiance and species selectivity of the extinction, since those are the parameters tested in the model. My guess is that this may not be the definitive answer as genetic mutation and adaptation and their capacity to spread across latitudes will play an additional mechanism/role in how populations are impacted. I also found the d13C component of output of the model not well resolved against the records, and the manuscript would have been much improved to see how some of the significant changes to e.g. mixed layer depth could be compatible with a range of sedimentary records of biogeochemical parameters throughout these periods. Validation with data did not take centre stage apart from being discussed in the text i.e. there were no figures that showed statistically that the model simulations were consistent with data from that period of time.

(Remarks on code availability)

Referee #3

(Remarks to the Author)
The manuscript "Selective extinction in the end-Cretaceous oceans driven by light loss" explores the selectivity in the marine extinction during the end-Cretaceous mass extinction using an Earth system model combined with a trait-based model.
I enjoyed reading the manuscript as it is written and structured very clearly. It builds very nicely on the modeling and proxy studies of recent years on this topic and combines insights from different studies in a comprehensive and menaingful way to get deeper insight in the selectivity of the extinction in the ocean and the linked processes. The results are not too surprising and have, to a large part, be suggested in earlier studies, as they build strongly on basic ecological mechanisms during extinctions. However, being able to model this and the way the authors build their study on the best selection of results of recent studies on the end-Cretaceous mass extinction is great success and very well done.

I have many remarks, I would guess most of them are rather minor comments, only one is major:

line 14 and 32: there has been a lot of research about the contribution of volcanism and/or asteroid impact to the end-Cretaceous extinction. Although it is almost certain that the impact had a large contribution it is still debated how relevant Deccan volcanism was. In the abstract the impact is mentioned as the only cause (l.14) and in line 32 of the main text it is stated that the impact is accepted to have caused the extinction by citing the very old 1980 Alvarez paper. This is certainly not justified. I suggest to rephrase these sentences and add that this ist still debated or that recent studies suggest a combination of both with the impact being an essential driver (cite for example Schoene et al. 2019, Hull et al. 2020)

line 6565-67: I suggest to mention the name of the trait-based model here and not only in the Methods section. I also suggest to make here already clear that the plankton ecotypes have been adapted for representing the end-Cretaceous extinction

l.69: I don't really understand this mechanisms and how this is implemented in the model and I am also not sure where to find the information the Method section. Can you either formulate this clearer here or refer more in detail where to find it in the Method section or point it out more clearly in the Method section?

l.75: I suggest to add the CO2 concentration used for the Late Cretaceous climate state as it gives together with the modeled temperature a good idea about the model

l.77: temperatures seem to match the proxy data quite well, but I guess it is high compared to other modeling studies (Niezgodzki et al. 2017, Berugger et al. 2021) - it seems like the model is doing a good job in reproducing the latitudinal temperature gradient which certainly was a problem of the mentioned modeling studies with the lower mean temperatures (as discussed in the SI of Brugger et al. 2021). Perhaps mention this as another achievement?

l.88: can you add briefly which substances are included in Senel et al. to get the solar reduction?

l. 97: for which post impact year are the numbers given for the mixed-layer depth? Would be interesting to compare with Brugger et al. 2017, they have the deepest mixed layer in the mid latitudes

Figure 1: could you shorten the pre-impact time (I guess 5 years is enough, actually only delta13C is changing during the pre-impact phase) in order to make the most relevant years after the impact better visible?

l.112 and Figure 2c: what are the surviving groups the percentage refers to? unclear to me in the text and the figure caption. (In the figure heading of 2c is a typo)

l. 123: MAJOR comment: you mention Brugger et al. 2021. They included additional nutrients coming from the impactor and show this is very relevant. How can you justify to not include this component? I suggest to add this (I would prefer this, but I have no idea how complicated this is to include to your model as a nutrient flux coming from the atmosphere to the ocean) or to find a good way to discuss why it works without doing so and discuss the expected changes
In addition, compared to Brugger et al. 2021 there is no overshoot of productivity - I can imagine that your model has a better representation of the involved processes, but it would be important to explain why you don't have this

l. 125/126: I don't fully understand the remark in the brackets - can you make this clearer, perhaps just by formulating a complete sentence?

l.141: why is this highly accurate? I find this an inappropriate term for a result of a modeling study with a very well-suited, but still very coarse and simplified model. In addition, I what does "reconstruction" refer to? Is it used here for the modeled isotope excursion? I suggest to use reconstruction in the context of isotopes in paleo studies only when it is reconstructed from proxy data, otherwise I find it confusing. Also, I don't see so much similarity to the isotope excursion in reference 36 which you mention here - can you explain what you mean or discuss the comparison of this reconstructed excursion with your modeled?

l.156: delete primarily?

l.167: why does temperature has an influence for high latitudes? Is it because of a limit in temperature under which it gets more difficult to survive?

Figure 3: contains interesting information but I find it a not so intuitive representation (all red looks like a very relevant contribution, but this is not the meaning). I don't have a good idea how to solve this, but just give this as a comment that I find this figure not so intuitively understandable although the content is actaully very basic. In addition, I think the design of the figure would need some improvement

l.195: make sure to call it surface ocean pH

l.196: it is justified to say that acidification played a secondary role, but I would rather discuss it from the perspective that the effect was only in few regions of relevance in contrast to call a change of 0.36 a mild change - this is actually in the order of magnitude of what was found in Henehan et al. 2019 to be a "mechanism for ecological collapse". Also, could you include information on how long the pH decline lasted (I would guess it recovers quickly? - this would also be an important information considering the effect on the oceanic ecosystem)?

l.197: I would not call it consistent with Brugger et al. 2021 - their region of saturation state below 1 is much larger although the effect is still limited to the high latitudes and therefore cannot explain a global extinction.

l. 211-213: Not sure about this: is it really the absolute reduction of light which determines the extinction risk? You should have less radiation in the high latitudes, so the percentage of reduction might still be large for the high latitudes and the amount of incoming radiation small at the high latitudes as well, so you cannot argue that there is a stronger light limitation and a linked productivity loss for the low latitudes, but it would rather be because of adaptation?

l.213/214: "Biologically, (sub)polar phytoplankton was already adapted to the low light environment" - could you add a sentence in the Methods section about how that works in the ECOGEM model?

l.232: Fig. 2e

l.262: why only dust here? In the introduction you mention dust, sulfate aerosols and soot

l. 263: what is the effect of the deepened mixed layer on the light availability? You only mentioned the deepening of the mixed layer in the main part of the manuscript as a mechanism transporting nutrients to the top of the ocean and you don't say anything about its effect on light availability

l.313: the obliquity of 23.5 degree is just the present-day value as there is no knowledge about the end-Cretaceous obliquity

l.324: can you say something on why it is justified to only run it for 100 years? is it stable afterwards? Or: which variables are

stable, which are not? In your figures it looked a bit like delta13C was not in equilibrium

l.333-339: a SI figure showing PAR with time would be helpful

l.369-372: are the reference to Fig S6 and Fig S7 correct? I guess in l.372 there should be Figure S6 referenced and perhaps it is Fig S7 instead of Fig S6 in l. 369? For Fig S7 there is nothing said on the shown influence of solar and CO2 effect. As I don't find it self-explanatory, could you add something on this or not show it in the figure?

(Remarks on code availability)
Due to time constraints, I only reviewed it briefly, but did not try to run it. My first impression is that it is well documented and the readme gives all important information to run it

Referee #4

(Remarks to the Author)
Selective extinction 1 in the end-Cretaceous oceans driven by light loss

Rui Ying, Fanny M. Monteiro, James D. Witts, Daniela N. Schmidt

Abstract: First read of the abstract. Very cool sounding. Novel. Synergistic.

This is an exciting and original paper that applies trait-based ecosystem modelling to the K-Pg mass extinction, potentially offering insight into of observed extinction selectivity. The idea that energy demand played a key role in extinction selectivity, over say skeleton mineralogy (impact related ocean chemistry changes including acidification), is novel, makes sense, and the results do a nice job of linking ecological traits to survival patterns including, some radical proposals about ocean stratification collapse, and a refined explanation for marine carbonate d13C signals. The approach is powerful, but some of the interpretations feel a bit too neatly packaged, and I'd like to dig into whether the patterns in the fossil record fully support the story being told.
Also, as with any modelling study, the details of the model matter (I ask for clarifications), and I have questions about how closely the modelling team have looked at the fossil evidence that is being claimed to support their model explanation. In particular I have questions about the actual planktonic foraminifera fossil record (a big feature in the modelling and K/Pg theory but side stepped at the evidencing stage here), also the way certain assumptions are set up—especially around the role of light/ darkness (and supposed prior ecological predispositions), the real relevance of body size when it comes to already tiny organisms, as well the marine plankton fossil evidence used to support some of the conclusions.
Otherwise, the paper is very well written, the figures professional and clear. If the authors can come back with clarifications and satisfactorily answers to my questions this is potentially of Nature quality.

• Title: Selective extinction in the end-Cretaceous oceans driven by light loss
-This title suggests your conclusions can be extended to all organisms? Do you think this is the case? This study focuses on marine ecosystems. I think it would be better if the title also sticks to that?

• Lines 39-41 "In the ocean, most nannoplankton, planktic foraminifera, all rudist bivalve and ammonoid cephalopod molluscs, many reptiles, and shark species went extinct, except for small and opportunistic taxa". Really!? Are we talking about all these groups here, i.e. do 'small opportunistic' versions of all of these groups exist and is a small reptile the same as small foraminifera (which are already tiny). I would like to see this size argument put in a little more context as we move between hugely different types of organisms. Plus, the model is all about plankton so are you confidenct can be extended to vertebrates?

• Lines 42-44: "Notably, sharks, rays, corals, and nannoplankton had lower extinction rates in the high latitudes or coastal regions than in the low latitudes, and these settings show less ecological disruption and reorganization across the K-PG." I notice there are no references for the extinction response of planktonic foraminifera at high latitudes. Plus, to my knowledge, ammonites, another calcareous group, suffered total extinction both at high and low latitudes. Please address this since these groups have the best fossil records, the vertebrate fossil record is incomplete and there are no high latitude mineralizable corals. This to me is very important to address.

• This current manuscript goes on to focus a lot on planktonic foraminifera, and these are arguably our best bio-evolutionary archive with a variety of insights into ecology and palaeoecology possible (excellent fossil record, well dated, ecological insights from modern species and geochemical constraints). Surely there is massive potential and available records to look at this, i.e. evidence greater extinction survivorship at high latitudes among planktonic foraminifera? No? Why are the PF high latitude papers not cited? What's missing? My quick search of the literature does not really bring up anything convincing. In fact, the K/Pg record from Site 738 reports evidence of a lot of fossil reworking at the classic high latitude site, meaning there is not convincing evidence for enhanced survivorship among planktonic forams. Huber, 1991. But perhaps your model predicts this for planktonic forams (Fig. 2 d). This part needs strengthening and a deeper coupling of the observational data with the results, albeit the different time scales under consideration here.

Huber, B.T., 1991. Paleogene and early Neogene planktonic foraminifer biostratigraphy of Sites 738 and 744, Kerguelen Plateau (southern Indian Ocean), in: Barron, J., Larsen, B., al., e. (Eds.), Proceedings of the Ocean Drilling Program, Scientific Results. Ocean Drilling Program, College Station, TX, pp. 427–449.

• Lines 59-60: If Gibbs et al., already used this trait-based model approach then what is new here? Feels like the text is missing a step that identifies the ‘gap’ in the Gibbs et al. study.

• For easy access, can you please clarify what “traits”- you mean: traits could be morphological but here I assume you are meaning functional traits , i.e. with a focus on how biological traits—such as size, metabolism, reproduction rate, or trophic/nutrient uptake efficiency—drive ecological processes like competition, predation, and resource cycling. A line on this would be helpful.

• Lines 67-68: does the model directly include shell mineralogy and how? Please state.

• Lines 69: ..”evoke a mechanism based on the minimal biomass required to maintain individuals…” How is this done?

I think you need a sentence saying, ‘using plankton as a tracer and emblematic of ecosystems’, otherwise the introduction suggests you are promising the whole ecosystem.

• Lines 89-90: “…based on sedimentological evidence combined with a climate model and indicated that photosynthetically active radiation (PAR) was nearly absent for 1.7 years after the K-Pg and fully recovered by the fourth year”. Please clarify why extinction resilience could not be a function of the existence of or possibility of dormancy strategies, i.e. ability to survive in some form for 2 years. Are there any empirical constraints on this for modern plankton for the key groups that there exist good fossil records? Please comment.

• Lines 110-111: Why are bigger plankton more sensitive to extinction? What is the meaning of size here? Please clarify with references relating size for already tiny things to energy needs.

• Lines 155-156, and lines 190-192. : is the preferential loss of calcareous plankton just a coincidence then? Explain.

• Lines 162-164: - “We first forced the whole plankton community with a constant pre-impact annual mean sea surface temperature while keeping other parameters the same”. Q. What do you think would happen if you extended the sensitivity experiments by repeating with different temperatures? i.e. could there be a lower temperature thresholds at which things behave differently?

• Lines 164-165: “Against expectations, temperature did not have a major impact on extinction patterns (Fig. 3d). “ I’d like to know more about where these expectations come from, in terms of citations. Please reference. What did you expect? I have to say I am also surprised since other model studies using planktonic forams as a model systems find that temperature is critical in terms of environmental responses?

Also, previous observations, including of planktonic foraminifera, find that e.g. test size is sensitive to temperature, i.e. because optimal living environments are temperature controlled (Schmidt et al., 2003). Other recent analyses of planktonic foraminifera biodiversity responses to climatic variability on various time scales also find that temperature IS a primary controlling factor in terms of response and habitat shifts, although new plankton ecological interactions are also important and creat latititudinally heterogeneous responses.

Swain, A., Woodhouse, A., Fagan, W.F., Fraass, A.J., Lowery, C.M., 2024. Biogeographic response of marine plankton to Cenozoic environmental changes. Nature 629, 616-623.

Woodhouse, A., Swain, A., Fagan, W.F., Fraass, A.J., Lowery, C.M., 2023. Late Cenozoic cooling restructured global marine plankton communities. Nature 614, 713-718.

Strack, A., Jonkers, L., C. Rillo, M., Hillebrand, H., Kucera, M., 2022. Plankton response to global warming is characterized by non-uniform shifts in assemblage composition since the last ice age. Nature Ecology & Evolution.

• Lines 183: “…., the modelled changes in the δ13C gradient across the K-Pg do not solely reflect variations in carbon export productivity as previously suggested (Fig. 1) because of the possible injection of light carbon into the atmosphere across the K-Pg.“ This is important. Are there any proxies to support the suggestion of the injection of light carbon into the atmosphere. I know that on longer time scales export productivity change does not seem to be correlated with stratification or terrigenous input and was inferred to be driven by changes in phytoplankton (Lowry et al., 2021). Moreover, Henehan et al., (2019), also with a GENIE model component to look at pelagic d13C gradients explained the observed pattern by a less efficient biological pump (enhanced upper ocean remineralization), balancing with the solubility pump? How do these results connect here?

• Lines 214-215; “Biologically, (sub)polar phytoplankton were already adapted to the low-light environment, as evidenced by their high chlorophyll –to-carbon ratio (Fig. 5a).” REALLY: CAN YOU SAY THAT? High latitude plankton are only adapted to low light 6 months of the year, the rest its high light? No? EXPLORE? “ThISE photoacclimation likely makes them more resilient to prolonged periods of darkness.”

• Lines 229-230: “Autotrophic organisms could directly use solar energy, whereas heterotrophs rely on the diminishing energy transferred from the primary producers“

-Note that N. pachyderma has adapted to food poor, light poor polar conditions, thus likely most resilient?

• Lines 230-231 “Demand-wise, a larger body size requires more energy/biomass to maintain basic metabolism, which translates into the higher vulnerability of large plankton to climate change (Fig. 2f). Do we really see such strong size effects in already tiny organisms? Is the difference between small /300 microns) and smaller 100 microns, significant? This needs some elaboration and refencering.

• Lines 238: benthic foram survival may also be linked to ability to survive low food supply: how long does a benthic foram live? Maybe it could surface for 3-4 years with minimal food?

• Regarding ocean circulation, the pattern of ocean response is very important to the biodiversity/survivorship response. Therefore, I want to know more about the robustness of the dramatic stratification collapse in the 2 years post impact.
-Firstly, does Genie produce a sensible representation of modern ocean circulation, including overturning circulation times (1000 years), and stratification, that would help support that the model is capable of producing a sensible simulate these processes well enough to capture short-time scale stratification responses for the K/Pg event? For example where is deep water produced by Genie in the modern, and where is it produced in the late Cretaceous? – (missing mixed layer depth plots for the late Cretaceous, also modern would be interesting).
-Genie is very simple compared with fully coupled ocean models, but would of these full complexity models also find this dramatic collapse of stratification? How plausible is it?

Figure 2d: Diversity of various plankton functional groups before and after the K-Pg impact. This figure shows that, dramatically the planktonic forams went 100% extinct whereas other plankton groups did not. Why? This is not fully dealt with in the text.
2e. It would be good to add the micro, nano and pico size classes, which are the categories used in the text, to the axis of panel e) for clarity.

MATERIALS AND METHODS
• Lines 285-289: Please explain further this model ecosystem. These are ‘fantasy’ components in a marine ecosystem, correct. You mention some foraminifera – are these assigned a skeleton mineralogy?

• Lines 303-304: Same as I raise above -Does allometry apply even for tiny, tinier and tiniest? Please supply some references bringing in the energy/size theory for tiny plankton across the micro, nano and pico plankton size spectra, and perhaps use these terms in the text.

I think the authors have largely done a good job of explaining and justifying the model parameterizations.

• Lines 322-323 ” …. Can you elaborate on “ (2) the difficulty of simulating the re-diversification process involving physical transport and evolution. This seems pretty fundamental to getting a realistic representation of the recovery process. How does this limitation impact the results?

• Lines 332-333: 2 years darkness, recovering after 4. How long can the different types of plankton survive without light? How much is known about this? Is this a parameter in the model? Please comment.

• Lines 355-357: “ To simulate their impacts on biodiversity, we evoked an extinction mechanism based on plankton individual's biomass”: What about a constraint on the ability of different types of zoo plankton to survive without food? E.g. can planktonic forams become dormant? Living constraints? If not, its worth saying this.

• Lines 371. There is something missing in this sentence.

(Remarks on code availability)

Version 2:

Reviewer comments:

Referee #1

(Remarks to the Author)
Dear Dr. Gee,

I am glad to have another opportunity to review this interesting manuscript by Ying et al. about the drivers of the K/Pg extinction in the marine realm. The revised manuscript is definitely improved, and I think the authors did a good job incorporating the suggestions and concerns of the reviewers, particularly concerning how the model relates to fossil data and paleoceanographic observations. In particular I think this version does a better job of explaining the limitations and assumptions built into the model, which was my main concern last time (although I do think the authors should say something more about water column structure changes – see below).

As I said in my first review, this paper addresses an important and long-standing question about the physiological drivers of the most recent major mass extinction in the marine realm. The authors go about this in a clever and novel way, and the fundamental result, that changing the light level can reproduce the observed extinction patterns, is exciting, and represents a major advance in our understanding of this extinction event. I don't think it's necessarily the last word on this subject (what ever is?) but it reframes the discussion after decades of work arguing back and forth about acidification and the "Strangelove Ocean," and substantively moves things forward based on a mechanistic understanding of marine ecosystems. I think the results presented here certainly deserve to be published in Nature.
There is one point in the response to the reviews where I felt like it would be productive to simply continue the conversation, and so I have pasted that section at the top with my new response (a response to the response to the reviewer), and then I have some additional comments and suggestions by line number below that. I think these amount to minor revisions, and would be happy to see this paper accepted once they're made.

Sincerely,
Chris Lowery
University of Texas at Austin

ORIGINAL REVIEW Line 82-84 – did plankton have specific depth habitats, as in the real ocean? This would be very important to mention because of the subsequent changes in ocean structure due to impact-induced cooling.

RESPONSE: We thank Dr. Lowery for his helpful comments and appreciation of the novelty of our study. We agree that the destruction of vertical habitat could have contributed to the extinction of tropical foraminifera. However, foraminifera only represent a small fraction of the total plankton biomass and diversity considered here (clarified in L19; L76; L86-89). Also, the hypothesis of destratification cannot explain the different extinction rates between calcareous and siliceous phytoplankton, both of which live in the upper ocean due to light needs. Overall, our model was designed to investigate extinction selectivity across the broader plankton community within the mixed layer, including 128 plankton functional types, many of which do not leave a fossil record. In terms of vertical structure, our model represents a vertically integrated plankton population while explicitly accounting for changes in the mixed layer depth. Our approach implicitly, yet efficiently, captures the effects of mixed layer deepening on light availability, temperature and nutrient supply.

We have added clarifications in the Method section (L346-352) to make this more explicit. Finally, we thank the reviewer for noting the misrepresentation of symbiont-bearing foraminifera. We have corrected the model configuration to use symbiont-bearing non-spinose foraminifera.

RESPONSE TO THE RESPONSE: Foraminifera are indeed a small fraction of the plankton considered here (and of course in the actual ocean) but they are representative of the zooplankton as a whole, which otherwise don't have a great fossil record. Additionally, we know from observations of modern plankton that depth habitat (as a physical niche, not a proxy for light or nutrient availability) is important across all groups. The euphotic zone where these phytoplankton live is often still depth-stratified in the tropics and mid-latitudes, with light penetrating to sub-thermocline depths (the DCM is often at or below the thermocline). Cermeño et al. (2008, PNAS) found that the relative proportion of nannos and diatoms is controlled by nutricline depth (which of course is closely related to stratification). Junger et al. (2023, Science) found that modern picoplankton communities vary with water mass (by depth and geographically) and that global community structure may be more controlled by factors related to horizontal and vertical dispersal than environmental drivers like nutrients. So, the physical destruction of depth stratified niches would be expected to cause the extinction of taxa adapted to those niches. This has been interpreted as the main driver of extinction of plankton during Cretaceous oceanic anoxic events (when global nutrient delivery to the euphotic zone *increased*), and this is not just for planktic foraminifera (e.g., Huber and Leckie, 2011 J. Foraminiferal Research), but also calcareous nannoplankton (e.g., Watkins et al., 2005, Paleoceanography) and radiolarians (e.g., Erbacher and Thurow, 1997 Marine Micropaleo). Also, destratification can explain the selective extinction of calcareous taxa the same way darkness can: high latitude assemblages, which tend to be dominated by siliceous taxa, are better adapted to long periods of darkness and live in an environment that is weakly or un-stratified, and so they experienced lower rates of extinction. (an aside: I am not sure how body size could explain the selection against calcareous plankton and it may be interesting to briefly address this, if you have an idea.)

My point here is that the effects of changes in mixed layer depth on nutrients, light, and temperature are certainly well-represented in your model, but the accompanying effects of the destruction of physical habitat space is not. This is fine! I think your model does a great job of illuminating the key role that darkness played in the mass extinction in the oceans, and this is an important advance! I just think this limitation needs to be acknowledged in the text. In particular, I think this would help explain why deep-dwelling low-latitude taxa (which were also presumably adapted to low light and could have just moved up to shallower depths) did not survive, a point also raised by reviewer #2.

New Comments
Line 34-35 – I'm sorry to contradict what another reviewer said but Hull et al. (2020) isn't an appropriate reference for the idea that Deccan volcanism was a driver. They investigated the role of volcanic outgassing and found major outgassing began and ended *before* the extinction and concluded that the extinction was caused by the Chicxulub impact, although post-extinction evolution may have been influenced by Deccan volcanism in the Danian. I would cite this in the next sentence ("However, overwhelming evidence supports…") instead. (I do like how you've framed these two sentences, though).

Line 39-40 – vaporization of anhydrite put the sulphur in the atmosphere but it was not the source of the carbon (note that

anhydrite doesn't have any carbon in it). The carbon instead came from vaporization of carbonates that made up the bulk of the target rock.

Figure 1 – in part (f) it would be good to extend the y-axis to zero, to make it clearer that some functional types survived, and provide an easy visual of the proportion that survived. As it is a quick glance makes it look like they went to zero.

Line 145 – you might note that the modelled result overestimates the extinction in planktic foraminifera, which did have a few survivor species. (it was 95% extinction, not 100%, which of course is not that much but also is the difference between the survival of the group and its disappearance).

Line 195 – I assume that the role of CO2 referred to here is in driving pH change, and I want to reiterate that CO2 was not the main driver of acidification at the boundary, but rather the rapid input of sulfuric acid and nitric acid via acid rain in the years corresponding to the impact winter (e.g., Kring, 2007 Palaeo3). I think this is important context for this discussion, because I don't think we'd expect CO2 alone to cause the observed ocean acidification, and so of course CO2 has a negligible impact in the model.

Line 201-203 – this is really interesting! It's one of those things that shows how completely unique the K/Pg mass extinction was, compared to all of the major and minor extinctions in Earth history.

Line 221-222 – ok but what about calcareous nannoplankton? Nobody would accuse them of being big, but they suffer a much more severe extinction compared to relatively larger diatoms.

Line 346-347 – I know plankton diversity decreases with depth but I am not sure that that means "most" of plankton diversity is found in the mixed layer. Can you add a citation here?

Line 386-393 – I think this paragraph is an important and succinct summary of the model and its limitations and it would be better to put it in the main text rather than the methods, if space allows. Maybe after the paragraph that begins at Line 75.

(Remarks on code availability)
I am still not a modeler, and so have not reviewed the code.

Referee #3

(Remarks to the Author)
Thanks for revising your manuscript so thoroughly, for answering so many of my questions and incorporating a lot of my feedback.

My two major comments have been addressed:
1.the nutrients from the impactor have been implemented and the results are discussed. Please make sure to correct the model name to CLIMBER-3+C and not CLIMBER-X which includes MOM3 and not MOM2 (in Figure S5 and in caption of this figure).
2. it is clear now why there is no overshoot and it seems very plausible.

An important improvement throughout the manuscript is the way the model is introduced, named and explained. It is very understandable and these parts read much better now.

I still have a couple of remarks/questions:

l. 97-98: this is based on my comment on the good performance of the model in reproducing the smaller meridional temperature gradient. I find the wording misleading, as it does not explicitly say that EcoGENIE succeeds in simulating a smaller temperature gradient in contrast to other models. I suggest to rephrase, for example: In contrast to other models (28, 37), EcoGENIE succeeds in simulating a reduced equator-to-pole temperature gradient, in agreement with temperatures from proxy data. Hence, it provides a realistic climatic environment to test extinction selectivity.

l.126: it is not an ocean model and I would also not call it complex, but rather „… results from the more complex model used in Brugger…"

l. 166 and Extended Data Figure 7: can you name the default run in a way it is clear what is included or at least describe it in the figure caption? And at least here, I find only Extended Data Fig.7 a of relevance

l.169: what does „high resolution" mean here?
Extended Data Fig.8: 1. do you compare here to the global mean? Make sure to at least mention as you compare regional data to a global mean signal (which might be acceptable for the ocean)
2. perhaps include ODP Site before the numbers?
3. I don't fully understand the caption of the figure and this is also not clear to me from the text: what are you doing with the isotope signature of the additional C - do you adjust it or not? I thought yes (and only find it justified to look at a figure like this in that case), but then I do not know what you want to say here with „do not subjectively tune"

l.188: do you mean with this sentence that you do not test the sensitivity to increased nutrients by a separate experiment? I guess you included the additional nutrients as in your combined case? or not? I guess this just needs some rephrasing to clarify

l.194: delete „Although“?

l.205: what is the reason for the extinction in the high latitudes when using pre-impact PAR? are the temperatures too low?

l. 219: do you really mean Fig 2d? I don’t see what this refers to?

l.267-270: this sounds like it was a new insight, but this was already discussed in Tyrrell et al. 2015 and Brugger et al. 2021

(Remarks on code availability)
Code and data availability: the link to the specific configuration of the code (which would be the most relevant one) did not work for me. In addition, I find it crucially to find the model output used and discussed in the manuscript somewhere. There was no information on that.

Referee #4

(Remarks to the Author)
This revised version represents a substantial improvement. I like the new title.

The abstract is much improved, better highlighting the trait-based approach, and more reflective of the study (“entire marine plankton community” works well). The incorporation of new sensitivity experiments, and the clearer linkage between extinction dynamics and body size, are also improvements.

In general I find that the theoretical framing now much improved, and most reviewer concerns seem to have been addressed.

However, several issues remain that must be clarified or strengthened before the paper is ready for publication, especially regarding the time scales of the model and alignment with fossil data. Part or this should be that the paper concludes that the model is a powerful hypothesis generator, but its predictions require more robust testing against available fossil evidence before the conclusions can be considered fully substantiated (see below).

1. Clarify model time scales and realism
I notice that the manuscript avoids any central statement or summary about the time scale over which the model operates. From Figure 1, it we see that the model explores processes on a century (~100-year) timescale, but this fundamental information should be stated explicitly and connected throughout the text. I think this is an important feature, i.e. the time scale of extinction response under consideration – this is almost real or near time (population experience of the catastrophe) whereas other studies might look at much longer term responses. Feels like that should be advertised in the abstract as well as woven through the text. Linked to this, I might have missed it but what is the time stepping regime in the model? Please lift this up.

A couple of places where this would benefit:
Lines 19–20: “Here, we use a trait-based ecosystem model… across the K–Pg boundary.”
Specify explicitly over what time period the simulations are run and the time steps (e.g. 100 years following impact?).

Lines 90–108: communicate how fast changes occur in the model and whether the time steps correspond meaningfully to real-world years (“years from impact” is shown in Fig. 1, but this needs explanation in the text).

2. Relationship between model outputs and fossil evidence
The paper still treats the relationship between model predictions and the fossil record somewhat inconsistently/unsatisfactorily.
Line 44: Jiang et al. (2010) is correctly cited as showing lower extinction rates at high latitudes for nannoplankton (phytoplankton)- which supports the model results. These data are taxonomic diversity measures.

Planktonic foraminifera fossil records, however do not show this pattern. That this is the case is important given that they have such a fantastic fossil record, including indicators of ecologic traits together with taxonomic diversity, and are celebrated in many other recent high profile studies as a ‘model system’, including these papers below.

Larina, E., Woodhouse, A., Swain, A., Lowery, C.M., Martindale, R.C., Myers, C.E., 2025. Regional restructuring in planktic foraminifera communities through Pliocene-early Pleistocene climate variability. Nature Communications 16.

Swain, A., Woodhouse, A., Fagan, W.F., Fraass, A.J., Lowery, C.M., 2024. Biogeographic response of marine plankton to Cenozoic environmental changes. Nature 629, 616-623.

Woodhouse, A., Swain, A., Fagan, W.F., Fraass, A.J., Lowery, C.M., 2023. Late Cenozoic cooling restructured global marine plankton communities. Nature 614, 713-718.

The rebuttal tries to side step the issue that the planktonic foram fossil record does not show lower extinction at high latitudes, as predicted by the model, by saying that "the foram fossil record only captures taxonomic diversity, whereas we model trait sensitivity". So why were taxonomic data acceptable evidence for the nannos? This needs some clarification.

Could this mismatch for the planktonic forams not be important on its own, rather than a flaw to step over? This is sort of there already but would benefit from focusing better.
It might even support the idea that a group level, group-specific traits (e.g. large, photosymbiotic micro-zooplankton that calcify) have their extinction selectivity modulated through compound kill mechanisms, e.g. for planktonic foraminifera starvation due to darkness and ocean acidification.
So better to 'own' that there is a mismatch with planktonic foraminifera (massively used group in evolutionary studies with a celebrated fossil record), which maybe supports the idea that depending on group traits (like being a larger microzooplankton calcifier), compound kill factors should be considered.

In this respect, some reflections on the distribution of certain ecological traits in plate Cretaceous planktonic foraminifera are important. Have the authors considered the evidence of distribution of key ecological traits, such as photosymbiosis, across latitudes in the late Cretaceous in this case? E.g. do data on planktonic foram assemblages from Maud Rise ODP Sites (689/690) include photosymbiotic taxa or were there fewer in the high latitudes during the late Cretaceous? Could this be part of the story?

Note some additional references relevant to the high latitude planktonic foraminifera records that are relevant.

-Keller, G., 1993. The Cretaceous-Tertiary boundary transition in the Antarctic Ocean and its global implications. Marine Micropaleontology 21, 1-45.

-Stott, L.D., Kennett, J.P., 1990. Antarctic Paleogene planktonic foraminifera biostratigraphy: ODP Leg 113, Sites 689 and 690., in: Barker, P.F., Kennett, J.P.e.a. (Eds.), Proc. ODP, Sci. Results. (Ocean Drilling Program), College Station, TX, pp. 549-569.

-Huber, B.T., 1990. Maastrichtian planktonic foraminifer biostratigraphy of the Maud Rise (Weddell Sea, Antarctica): ODP Leg 113 Holes 689B and 690C, in: Barker, P.F., Kennett, J.P., al., e. (Eds.), Proceedings of the Ocean Drilling Program, Scientific Results. College Station, TX (Ocean Drilling Program), pp. 489–513.

3. References and fossil context
The extinction process section (around Line 38) is missing relevant literature on post-K–Pg planktonic foram rediversification uncertainty—e.g. whether new planktonic forms evolved from benthic ancestors or surviving planktonic lineages. (Olsson et al., 1999 vs Arenillas et al., 2022; Darling et al., 2009; Morad et al., 2022).

Olsson, R.K., Hemleben, C., Berggren, W.A., Huber, B.T., 1999. Atlas of Paleocene planktonic foraminifera. Smithsonian Contributions to Paleobiology 85, 252.

Darling, K.F., Thomas, E., Kasemann, S.A., Seears, H.A., Smart, C.W., Wade, C.M., 2009. Surviving mass extinction by bridging the benthic/planktic divide. Proc Natl Acad Sci USA 106.

Morard, R., Hassenrück, C., Greco, M., Fernandez-Guerra, A., Rigaud, S., Douady, C.J., Kucera, M., 2022. Renewal of planktonic foraminifera diversity after the Cretaceous Paleogene mass extinction by benthic colonizers. Nature Communications 13, 7135.

Arenillas, I., Arz, J.A., Gilabert, V., 2022. An updated suprageneric classification of planktic foraminifera after growing evidence of multiple benthic-planktic transitions. Spanish Journal of Palaeontology 37, 1-34.

4. Body size and extinction mechanisms
Generally, the stronger focusing on body size works well. The improved background theory in relation to this is an improvement.
In this respect, regards planktonic foraminifera, I suggest you refer to them as 'microzooplankton' since they are certainly not large among the zooplankton generally.

Line 220: Correct the statement—rewrite as : "planktonic foraminifera are large within the microzooplankton community"

5. Model–data comparison and sensitivity tests
Line 186: "The strong model–data agreements allow…" — please verify this claim carefully, as the fit is not uniformly strong across groups. As far as I can see the model data-agreement involves a short written presentation of some fossil record data types (Lines 45-47):

"Notably, high-latitude nannoplankton exhibited lower extinction rates than low-latitude counterparts7, though such latitude-dependent extinction was not found in foraminifera 19 and molluscs 20."

I would say that this is not strong model agreement. The two key groups with excellent fossil records, planktonic foraminifera and molluscs we are told do not show latitude-dependent extinction patterns. Is the type of plankton that do show a pattern also important: nannoplankton = calcareous nano phytoplankton?

7. Conclusions seciton
I suggest that the time frame of extinction should feature in the conclusions. See above.

Considering my comments above, it is also important to state that the model remains a powerful hypothesis generator, with some fossil data support, but its predictions require more robust testing against available fossil evidence before the conclusions can be considered fully substantiated.

Figure 3: Please define PAR (photosynthetically active radiation) clearly in the caption.

(Remarks on code availability)

Version 3:

Reviewer comments:

Referee #1

(Remarks to the Author)
Dear Dr. Gee,
I am satisfied with the revisions that the authors have made and think this paper is ready for publication with a couple very minor changes listed below. I do not need to see it again.

I really like how the authors frame the ongoing uncertainty about specific mechanisms of extinction in the marine realm in the introduction, nicely setting up both the scientific motivation of this work and the use of a mechanistic ecosystem model to address these questions.

The paper does an excellent job of walking through the results of the model runs, their implications, and their limitations. It makes a compelling argument for the importance of energy demand and plankton body size in explaining the selectivity of the K/Pg extinction, and how that can be explained through darkness after the Chicxulub impact. I think it does more than simply serve as a "hypothesis generator" as another reviewer puts it (it does a pretty good job of testing hypotheses for extinction in my opinion), but importantly in light of my earlier reviews I think the authors do a good job stating the limitations of their model and not overstating the strength of the interpretations (which are very strong! just not bulletproof).

Discussions of the mechanisms of the K/Pg mass extinction in the oceans have bounced around the same questions for a few decades now about acidification and Strangelove Ocean vs. Living Ocean, etc., and I think the introduction of ecosystem modelling will reframe the conversation in helpful ways to move us all forward to new questions. This paper thus represents an important step forward in our understanding of the K/Pg mass extinction in the plankton (I will certainly be citing it a lot moving forward) and I think it absolutely deserves to be published in Nature.

Best,
Chris Lowery
University of Texas

Line 156-157 – Here's a pedantic foram point: I don't think anyone debates that at least one planktic foraminifera (Guembelitria cretacea) survived the extinction, though there are some debates about other possible survivor species which really just get into how likely you think that some short-ranged species actually lived in the Danian or were just reworked for a little while (the number of these broadly considered survivors is actually pretty low so one could argue that the current estimate underrepresents survival). Raphael Morard's work suggests that Cenozoic planktic foraminifera had a benthic ancestor, but that does not mean that there were no survivors, just that the survivors have no modern descendants (as Morard et al. point out in their paper). So, observations probably don't overrepresent survival.
Line 186 – "early" Danian is not a formally defined substage and so early should not be capitalized

(Remarks on code availability)

Referee #3

(Remarks to the Author)

I enjoyed reading the manuscript again a lot! It reads very nicely. My comments have all been adressed and I only have two comments linked to two of my comments from the last review round. Both are not critical, they do not necessariliy need to be adressed.

l.169: what does „high resolution“ mean here?
Responses: We meant high temporal resolution in Hull et al. (2020)'s d13C data. To avoid the confusion, we removed 'high resolution' here.

no, this is actually not removed here, it is still here. In principle I don't have a problem with calling it "high resolution", as it is high resolution compared to other records, but I would find it important to clarify here that high temporal resolution of the proxy record for KPg is still not comparable to modeled temporal resolution, as it is only possible to say the record has a resolution between yearly to 100 to 1000 years, so this will stay a limitation (which does not mean that I find it not justified to compare it anyways). You discuss this in the end of the paper, but here it sounds like the temporal resolution is the same for the record and the modeled results. As far as I know Hull et al.(2020) can also not limit the resolution to smaller intervals like yearly?

l.188: do you mean with this sentence that you do not test the sensitivity to increased nutrients by a separate experiment? I guess you included the additional nutrients as in your combined case? or not? I guess this just needs some rephrasing to clarify
Responses: We clarify that we have included the increased nutrient flux in the main combined experiment, but did not conduct a sensitivity experiment. We changed it to "we do not assess nutrient impacts in the sensitivity experiment".

ok, I understand now what you want to say with this sentence. I actually find this sentence not necessary and I wold find it clearer to just delete it, but this is up to you.

My only additional comments refer to the documentation of the model output - here, I see some important information still missing, see below.
If this is adressed, I recommend to accept the paper for publication.

(Remarks on code availability)
I did not try to run the code, but I looked at the structure of the code and the documentation in the readme. For the code this looks good to me.
For the model output, it is not clear to me which files I need to reproduce figures and results. This is not documented in a readme and there is a huge amount of files I would not know what to do with in the zip folder which I downloaded from the zenodo link. In my opinion it is necessary to provide a readme with the model output stating which output files have been used for the figures and for the calculated results. In addition, the scripts to reproduce the figures and results should also be provided (I did not find them), together with comments and a readme file.
I addition, I suggest to not upload one huge compressed file, but compress the directories, which were in there, separately and put the readme(s) not inside a compressed file (then I can find out what to download for a certain question and do not need to download all the data).

Referee #4

(Remarks to the Author)
I am largely satisfied that the authors have addressed my main concerns from the previous review. In particular, the clarification of model time scales and timestepping is now clear and appropriate, which improves the paper.

Regarding data comparison, I focus on the planktonic foraminiferal fossil record, which remains the best available dataset. While gaps remain—particularly concerning ecological trait distributions and continuous high-latitude Cretaceous records—the authors now acknowledge these limitations. A few final clarifications would strengthen the paper

1. Functional type distribution (Lines 85–87): Are the 16 functional types distributed across latitudes in a realistic manner, for example relative to modern observations? This is important because traits such as photosymbiosis are not evenly distributed across latitudes in the modern or Pleistocene oceans, being largely absent at high latitudes. The authors' prior work (Ying et al., 2023, 2024) modeled these distributions in detail, but this is not explicitly referenced here. Please clarify how realistic latitudinal trait distributions are implemented in the model.
References:
• Ying, R., Monteiro, F. M., Wilson, J. D., Ödalen, M., & Schmidt, D. N. (2024). Past foraminiferal acclimatization capacity is limited during future warming. Nature, 636(8042), 385–389.
• Ying, R., Monteiro, F. M., Wilson, J. D., & Schmidt, D. N. (2023). ForamEcoGEnIE 2.0: Incorporating symbiosis and spine traits into a trait-based global planktic foraminiferal model. Geosci. Model Dev., 16(3), 813–832.

2. Extinction selectivity at high latitudes with respect to planktonic forams: I am still unclear on how the scarce high-latitude fossil records support lower extinction selectivity during the K/Pg event. High-latitude sites show lower pre-extinction taxonomic diversity, which implies lower functional trait diversity (modern high-latitude faunas also lack photosymbiotic taxa). Does lower starting functional diversity truly indicate reduced extinction selectivity, or could it instead reflect the predominance of generalist or opportunistic taxa?

Although the study does not focus on planktonic foraminifera, the argument could be strengthened by considering the ecological traits of likely survivors and pre-extinction distributions for this group- mention in the supp infor maybe?. Current evidence suggests very few true survivors globally. Guembelitria cretacea, widely interpreted as non-symbiotic, opportunistic, and stress-tolerant, is the clearest likely survivor. Early Paleocene high-latitude occurrences of Hedbergella and Heterohelix may represent survivors, but reworking cannot be excluded. Other common pre-K/Pg genera, such as Guembelitrioides, were likely not survivors and were non-symbiotic.
This suggests that high-latitude assemblages had few specialized ecological traits prior to the extinction. Keller and Pardo (2004) provide a key reference mapping these high-latitude occurrences and highlight the dominance of opportunistic Guembelitrinidae. How does the model reconcile this with its finding of lower extinction selectivity for planktonic foraminifera? This is where clarify how the initial trait distributions at high latitudes are represented in the model is important, and how they inform the simulated extinction patterns.
References:

• Morard, R., Hassenrück, C., Greco, M., Fernandez-Guerra, A., Rigaud, S., Douady, C. J., & Kucera, M. (2022). Renewal of planktonic foraminifera diversity after the Cretaceous–Paleogene mass extinction by benthic colonizers. Nature Communications, 13, 7135.
• Keller, G., & Pardo, A. (2004). Disaster opportunists Guembelitrinidae: Index for environmental catastrophes.Marine Micropaleontology, 53, 83–116.
• Huber, B. T. (1996). [Relevant pre-K/Pg planktonic foraminiferal taxonomy.]
• Olsson, R. K., et al. (1999). [Early Paleocene high-latitude records.]

Overall, given the limitations of the fossil record, the authors' arguments are reasonable, and the hypothesis is appropriately framed. The phrase “and potentially planktic foraminifera19” adequately acknowledges these limitations, but explicit discussion of functional trait distributions would further clarify the modeling results.

(Remarks on code availability)

Version 4:

Reviewer comments:

Referee #3

(Remarks to the Author)
Thanks for addressing my comments again and especially for providing all data and scripts, a helpful readme and for saving the data in separate zip files. It is clear how to use the data now. I agree that the paper can be published.

Julia Brugger

(Remarks on code availability)
as stated above, this is fine now!

Referee #4

(Remarks to the Author)
Thanks for recognizing the these important aspects of the planktonic foram records - this is satisfactoraly is acknowledged now, although its a shame that a lot of important information about the mechanistics of the model and the sensitive parameters (like distriibution of photosymbiosis) gets shuffled to the SI section.

The manuscript now states in the Abstract (line 24) and main text (line 47) that the model reproduces “reduced diversity loss in high-latitude taxa”, citing Ref. 7 for observational support. However, there are issues with the nannofossil records too, and the cited study on calcareous nannoplankton (Jiang et al. 2010 K/Pg nannoplankton extinction latitudinal patterns) does not provide evidence for a high-latitude refuge effect in the way implied here.

While the study by Jiang et al reports substantial geographic variability in extinction intensity, the dominant pattern identified is hemispheric asymmetry, with the highest extinction levels in the North Atlantic, North Pacific and Tethyan oceans and lower extinction intensities in the Southern and Indian oceans. Importantly, this pattern is not interpreted as reduced diversity loss at high latitudes, but rather as a consequence of the physics of the impact event. The authors conclude that an oblique, northward-directed Chicxulub impact concentrated ejecta in the Northern Hemisphere, suppressing photosynthesis there and producing the observed extinction gradient.

Thus, the cited reference supports hemispheric differences linked to ejecta distribution, rather than preferential survival of high-latitude taxa. The manuscript should revise the text to reflect the conclusions of the cited observational work more accurately.

This hemispheric asymmetry aspect of the Jiang et al paper's conclusions will of course not be in this current model, but the details of the nannofossil observations should not be glossed over. As written, the text presents the observational evidence

as supporting a high-latitude refuge signal that is not clearly demonstrated in the cited study. This must be addressed.

(Remarks on code availability)

Open Access This Peer Review File is licensed under a Creative Commons Attribution 4.0 International License, which permits use, sharing, adaptation, distribution and reproduction in any medium or format, as long as you give appropriate credit to the original author(s) and the source, provide a link to the Creative Commons license, and indicate if changes were made.
In cases where reviewers are anonymous, credit should be given to 'Anonymous Referee' and the source.
The images or other third party material in this Peer Review File are included in the article's Creative Commons license, unless indicated otherwise in a credit line to the material. If material is not included in the article's Creative Commons license and your intended use is not permitted by statutory regulation or exceeds the permitted use, you will need to obtain permission directly from the copyright holder.
To view a copy of this license, visit https://creativecommons.org/licenses/by/4.0/

Responses to reviewers

Overview

We thank our reviewers for their constructive comments and the editor for the opportunity to revise and improve our manuscript. Below, we provide a summary of the main revisions, followed by detailed, point-by-point responses to each reviewer comment. Reviewer comments are shown in grey with our responses in black.

Main concerns	Actions
Unclear model novelty (Reviewer #2)	We clarify our novelties now: (1) our study introduces the first trait-based ecosystem model that explicitly simulates marine plankton extinction, overcoming previous issues of unrealistic immediate recovery and productivity overshoot after the K-Pg event; (2) by mechanistically linking extinction to body size, the model reproduces observed fossil patterns. (3) coupled with a 3D ocean circulation and biogeochemistry framework, it quantifies regional extinction differences and impacts on biological pump efficiency. Unlike prior studies such as Gibbs et al. (2020), which focused on post-extinction recovery, our approach directly addresses the extinction process itself.
The lack of key model components, including vertical habitat, nutrient fluxes (Reviewers #1,3)	As requested, we have added iron recycling and projectile-derived nutrient fluxes into the model. We also corrected the modelling of symbiont-bearing foraminifera from spinose to non-spinose. These modifications do not change the main results, as nutrients were already sufficient due to enhanced mixing in the initial manuscript, and all foraminifer groups still go extinct.<br><br>Our model also captures the effects of mixed-layer deepening on plankton dynamics, including changes in light and nutrients, by representing a vertically integrated diversity and biomass of plankton within the mixed layer.
Lack of method details and request of more validations (Referee #2,4)	We have added further details on the model structure and extinction mechanism in the Method section (L322, L365) and Supplementary Information.<br><br>We have also expanded our model validations by including:<br>(1) Late Cretaceous vertical carbon isotopic profiles, ocean circulation, and pre-industrial model performance (SI);<br>(2) Extended model simulations up to 5 kyr after the K-Pg boundary to allow comparison with proxy evidence:<br>• the K-Pg carbon isotope excursion (Extended Data Fig. 8)<br>• Ocean deoxygenation (Extended Data Fig. 9)<br>• Enhanced $CaCO_3$ preservation following alkalinity changes during the early Danian (Extended Data Fig. 9)<br><br>However, directly comparison with fossil diversity remains limited because (1) fossil data are sparse and biased; and (2) our model simulates functional diversity rather than taxonomy diversity.
Other notable changes	We have conducted additional sensitivity tests to assess the robustness of our findings. These include simulations in which plankton extinction is prevented and where the POC:DOC export ratio is made independent of plankton size, allowing us to evaluate their respective impacts on the biological carbon pump and patterns of extinction.<br><br>Additionally, we identified and corrected an error in the originally reported CO_2 emissions. The correct value is 1750 Pg C, half of the initially stated 3500 Pg C. This revision has been validated and updated throughout the manuscript.

Referee #1

Ying et al addresses the important and long-standing question of the physiological drivers of K/Pg mass extinction in the marine realm. ... However, it is not the last word on this problem because it does not include depth habitat. The greatest diversity of calcareous plankton is in the tropics, while the greatest diversity of siliceous plankton is near the poles. It is possible, as suggested in this manuscript, that the preferential extinction of former is due to the changes in energy availability during the impact winter .... BUT it is also possible that the preferential extinction of calcareous plankton was due to the destruction of their depth habitat ...I tend to think that it's both, ...

As it is, I did not see anything in the model methods about depth habitat, and because depth habitat is so important to plankton diversity I think that this *must* be addressed to bolster the conclusion that global darkness was the key factor in the extinctions. .. but I do think that depth habitat, and its implications for the results presented here, needs to be discussed in detail.

The only other major point I want to bring up here is that the planktic foram functional groups in the model are spinose symbiont-bearing and non-spinose non-symbiont bearing. ...This should be addressed and justified in the methods.

Line 82-84 – did plankton have specific depth habitats, as in the real ocean? This would be very important to mention because of the subsequent changes in ocean structure due to impact-induced cooling.

Response: We thank Dr. Lowery for his helpful comments and appreciation of the novelty of our study. We agree that the destruction of vertical habitat could have contributed to the extinction of tropical foraminifera. However, foraminifera only represent a small fraction of the total plankton biomass and diversity considered here (clarified in L19; L76; L86-89). Also, the hypothesis of destratification cannot explain the different extinction rates between calcareous and siliceous phytoplankton, both of which live in the upper ocean due to light needs. Overall, our model was designed to investigate extinction selectivity across the broader plankton community within the mixed layer, including 128 plankton functional types, many of which do not leave a fossil record.

In terms of vertical structure, our model represents a vertically integrated plankton population while explicitly accounting for changes in the mixed layer depth. Our approach implicitly, yet efficiently, captures the effects of mixed layer deepening on light availability, temperature and nutrient supply. We have added clarifications in the Method section (L346-352) to make this more explicit.

Finally, we thank the reviewer for noting the misrepresentation of symbiont-bearing foraminifera. We have corrected the model configuration to use symbiont-bearing non-spinose foraminifera.

Line 1 - A suggestion on the title – a shorter and more easily understood way to say "light loss" would simply be "darkness" (Also, in my opinion, "darkness" is more evocative)

Response: We have revised the title accordingly.

Line 15 – a pedantic point here but it's 75% of species in the *fossil* record.

Line 31 – another pedantic point: the Yucatán wasn't a peninsula when the asteroid hit, it was a carbonate platform.

Line 32 – Oh boy 3 pedantic points in a row, I'm sorry about this: Alvarez et al. didn't know anything about the location of the crater so you should cite another paper here too (Schulte et al., 2010, Science, is the classic, but if you're pressed for references you could cite Morgan et al. here, too)

Line 35 – the target rocks was not gypsum ($CaSO_4$ $2H_20$), but anhydrite ($CaSO_4$), as is well documented from boreholes across the Yucatan. See Brett et al. 1992, for example.https://www.sciencedirect.com/science/article/pii/0016703792904069

Response: We have corrected the terminologies and cited Morgan et al as suggested. Additionally, we have clarified the chemical formula of anhydrite for non-specialist readers (L40).

Line 40 – there might be something screwy with your references here
....Line 41-42 – I'm not sure these are the best references for this statement. Thomas et al. 2007 (GSA Special Paper 424) and/or Culver, 2003 (Marine Micropaleontology), are better references for benthic foram survivorship than MacLeod.
Line 47 – the role of ocean acidification at the K/Pg boundary has been discussed as far back as Prinn and Fegley, 1987 (EPSL) MacDougall, 1988 (Science)
Response: Thank you for suggesting more appropriate references. We have incorporated them accordingly.

Line 49 – I'm not sure if the original K/Pg impact hypothesis put forward by Alvarez et al. should be described as an "alternative hypothesis." I'd probably switch this sentence with the OA sentence, so the impact winter is first and OA is the "alternative"
Response: Thanks for pointing this out, we have switched the order as recommended.

Line 51-52 – why couldn't productivity collapse explain the observed geographic patterns? Different parts of the ocean have different amounts of primary production, with different seasonalities, supporting ecosystems that are adapted to different amounts and types of food. Why couldn't have those ecosystems responded in geographically distinct ways to a reduction in primary production?
Line 53 – limited AND geographically heterogeneous (see Hull and Norris 2011, for example). ....
Response: We agree and have now incorporated this perspective into the main text (L53-55) to reflect this possibility.

Line 67-68 – why are foraminifera separate from zooplankton?
Response: The model distinguishes foraminifera from non-calcifying zooplankton to characterise the broader plankton community, including non-fossilised types. We have clarified this by using "other/generic zooplankton"" (e.g., L83) in the revised paper and provided further explanation in Method (L357-359).

Line 83-84 – is this similar to diversity trends in the Cretaceous ocean, though? It was a fundamentally different global ocean than our current icehouse world and we know that, for example, the modern latitudinal diversity gradient only emerged 15 million years ago (Fenton et al., 2023 Nature).
Response: The model simulates functional diversity grounded in the ecological theory that higher nutrient availability supports a greater number of coexisting size classes (Ward et al. 2014; Journal of Plankton Research). Such ecological theory is based on an idealised zero-dimension model and should not be limited to specific time as it is not bounded to a certain geography and ocean state (see their Fig. 4). We clarified this in L103-105.

Nevertheless, we acknowledge the difficulty to validate our diversity pattern due to the lack of fossil records' preservation and sampling biases. Therefore, we added in L105-107 to suggest that our model-based estimate of functional diversity provides a complementary perspective, helping to overcome limitations of fossil data and to better capture broader aspects of plankton community structure and extinction risk.

Line 85 – typo ("event")
Line 86 – "late" should be capitalized, as the Late Cretaceous is a formal Epoch.
Response: corrected.

Line 87 – I think this 700 ppm CO2 pulse needs further explanation. I...The timing seems very important here if you're evaluating OA as an extinction mechanism, as quicker acidification is going to be more damaging to pelagic calcifiers. I see you partially address this further down (Line 195) but I think it would be worth mentioning up here too, so you don't have people like me wondering about it for 100 more lines.

Response: Thank you for pointing this out. The 700 ppm CO_2 pulse is taken directly from boron isotope data and used here only as an input, not to reconstruct pH change (which does not influence foraminifera in our model). We now clarify this at L113-115.

Line 126-128 – just like the real post-K/Pg ocean, you should point out! Also, what does the change in plankton size do to export flux, and to nutrient cycling in the euphotic zone? Did a shift to smaller plankton keep more nutrients in the upper ocean?
Response: Yes! To investigate this further, we have added a sensitivity experiment keeping POC:DOC export ratio independent of plankton size, which ultimately controls nutrient recycling in the mixed layer (Supplementary Information L195). The results show that a shift to smaller plankton reduces export by 30% and hence retains more nutrients in the upper ocean, consistent with observations. We also now highlight this agreement in the main text (L161).

Line 155-158 – but no one thinks CO_2, specifically, was what caused acidification, it was sulfate and nitrate aerosols converted into sulfuric and nitric acid (in addition to carbonic acid from the CO_2). Does your model include anything about the other, more important climate active gasses? (and if not, you should explain how that impacts your interpretations in the text).
Response: Our model does not include sulphur and nitrogen aerosols, as it cannot directly simulate their acidification impacts (L117 in supplementary information). This makes our interpretation of extinction risk for calcareous plankton conservative (L361-363). However, our results suggest that ocean acidification was not the primary extinction mechanism, as we are able to reproduce most observed fossil record patterns through changes in light availability alone (Fig. 3). We now clarify this limitation and its implication in the text.

Line 172-175 – I think this is exactly right. The drop in productivity was severe but not long enough to filter down to the deeper depths of the ocean.
Line 178 – this is in line with predictions by Henehan et al. (2016) and observations by Lowery and Bralower (2022)
Response: Exactly. We have added both references to support this point (L259).

Line 219 – does this post-K/Pg elevated biomass and diversity in the polar oceans in the model match observations (e.g., from Seymour Island?)
Response: There might be some misunderstanding on this point (and we welcome further clarification from the reviewer). Both our modelled diversity (Fig. 2) and productivity (Extended Data Fig. 4) are lower (not higher) in the polar oceans. This trend aligns with recent proxy evidence for a high-latitude productivity decline during the K-Pg interval, as observed in the Seymour Island section (da Silva et al. 2023, Marine Micropaleontology).

Line 235 – what is "neritic feeding" and how is it different than the grazing that non-photosymbiont-bearing forams do in the open ocean?
Response: Thank you for pointing this out. We meant detritus feeding and corrected the text accordingly (L239).

Line 301 – note that spines as a trait did not exist in the Cretaceous foraminifera. They evolved in the early Danian in Eoglobigerina. Cretaceous photosymbiont-bearing planktic forams were all non-spinose.
Response: Thank you for this important clarification. We have resolved it by simulating symbiont-bearing non-spinose foraminifera in our revised model experiment.

Referee #2:
Much as the science is interesting, I did find myself questioning quite what the model was actually doing here to offer something new to the table. It may have been the way the manuscript was written which always discussed "the model" without really saying **what features within the model were of interest or were providing any kind of mechanistic insight or understanding**. .... reader is never

told how these are parameterised in the main text nor really can the reader follow the reasoning behind why this is so mechanistically important and quite what result the model generates to draw this conclusion The writing was incredibly opaque with regards to true insights revealed as opposed to testing a hypothesis of sensitivity when it was prescribed.
Response: We acknowledge that the original manuscript did not provide sufficient detail on the model's design, novelty, and mechanistic insights, which limited clarity. We have substantially revised the text to address these issues (L322, 356; Supplementary Information).

- **Novelties**: Our study presents the first trait-based ecosystem model that explicitly incorporates marine plankton extinction, a key advancement allowing us to dynamically simulate the regional diversity changes (L63-66). Unlike previous approaches, extinction in our model is mechanistically linked to body size, which successfully reconstructs extinction patterns seen in the fossil record (Extended Data Fig. 6). Moreover, our model is coupled to 3D ocean circulation and biogeochemistry framework (L78), unlike Gibbs et al. (2020), allowing us to quantify regional differences in extinction and its impacts on biological pump efficiency (Extended Data Fig. 7). While Gibbs et al. (2020) focused on post-extinction recovery, our study addresses the extinction process itself, which their framework could not resolve due to the lack of extinction mechanism.

- **Mechanistic insights**: Our study reveals that extinction patterns across plankton groups are primarily driven by impact-induced darkness interacting with body size-dependent survival. Although we cannot fully discount contributions from ocean acidification, sensitivity tests show these effects likely play a smaller role. This work provides new mechanistic understanding of why certain groups and regions were disproportionally affected at the K-Pg boundary.

- **Model parameters**: We now explain how the model dynamics work throughout the manuscript (L75-89, Method and Supp. Mat.). Our model does not assign different sensitivities to light, CO_2 or temperature, nor does it impose predefined ecological niches in simulating plankton dynamics (clarified in L386). Instead, differences among plankton groups arise from physiological traits, including body size and trophic strategy. Therefore, the success of plankton groups and biodiversity are emergent properties of the model, rather than preset assumptions.

- **Clarity of Writing**: We have revised the manuscript throughout to improve structure and presentation. Key changes include: (1) clearly introducing limitations of existing approaches before presenting the novelty of our framework; (2) more explicitly integrating the model results with observations to strengthen our arguments (e.g., L154-176); (3) integrating the discussions of drivers of selectivity in [**Drivers of selective extinction**] and move the comparisons with previous studies to [**Reconciling with previous studies**].

I also have a scepticism over the issue of adaptation of organisms within the model. ... Typically the population sizes get smaller with larger organism size- which then means that adaptation is much slower. So to what extent could this provide an alternative explanation to the differential extinction, but is not thrown up by the model as it is not put in as a parameter to test? I also wonder what role this could play in the ideas about low light adaptation and how fixed this may be? Perhaps there are oceanic fronts which may limit mixing within the population but again this is not discussed?
Response: We agree that our model does not simulate evolutionary processes such as adaptation, so we cannot rule out their potential role in shaping differential extinction. However, adaptation occurs across generations and need sufficient population size for natural selection, with larger population more likely to adapt and survive (as reviewer commented). This effect is implicitly represented in our model simulating population size, which is used to determine extinction/survival (L369, L382). The success of our size-based extinction threshold (as we highlight in the revised paper) also indirectly reflect the advantages of having small size but large population size.

In relation to light limitation, while true evolutionary adaptation is not represented, the model does include photoacclimation using a revised Geider 1998 model, allowing phytoplankton to dynamically adjust (so, not fixed) their light-capture efficiency (via variable Chl:C ratio) depending on light availability (L390-392).

Finally, the reviewer refers to the role of ocean fronts in preventing plankton from spreading across latitudes. This is possible, but ocean is generally well connected, and dispersal is generally not an issue for plankton (e.g., Darling et al. 2000, Nature). The K-Pg oceans were characterised by even stronger mixing with worldwide tsunamis. Therefore, it is more likely that low-latitude plankton cannot tolerate the high-latitude environment than that dispersal limits them to migrate. B. Ward et al. (2021, PNAS) have reached the same conclusion that environmental selection places strong constraints on global dispersal. We clarify this in L383-385.

Minor comments:
Line 68-71 Need to unpick this a bit more for the reader. It is very much not clear from the main text what parameters are being placed into the model that will allow a new insight into the extinction mechanism.
Response: we have added a new section in Method (L365, Fig. S1) and revise this paragraph to more clearly explain the extinction mechanism, and to highlight why this represents a novel approach not previously applied in a biogeochemical model with a trait-based ecosystem component.

Line 75: "We first applied the model". Both here and in the methods, it is really hard to understand what is unique about the model, what parameters allow the derivation of the conclusions about differential extinction based on energy supply and demand. ..This has to be outlined clearly so that it can be assessed.
Response: We have highlighted the novelty of our model and the parameterisation that underpin its ability to simulate differential extinction. See comments and responses above.

Line 77: It appears that sea surface temperature and pH align with proxy data but what about estimates of deep ocean temperature and e.g. deep ocean oxygen content for example. ... It would be helpful to see a range of biogeochemical parameters that are produced via this modelled scenario to understand whether the outcomes are reasonable against the geological/sedimentary record. Certainly, the changes to PO4 in surface waters seem pretty extreme.
Response: Deep-sea oxygen level in the model initially increases due to enhanced mixing before gradually declining on kyr timescale (Extended Fig. 9). This trend is consistent with observations from proxy and fossil records (Kaiho et al. 1999 *Paleoceaongraphy*; Vellekoop et al. 2018 *Geology*). The model also reproduces higher benthic alkalinity post-impact (Extended Fig. 9), matching improved CaCO3 preservation observed in the early Danian sedimentary record (Henehan et al., 2016 Philosophical Transactions of the Royal Society). Surface PO4 in the model exhibits a substantial but short-lived peak in the first few years, caused by enhanced mixing and the collapse of primary production, before returning rapidly toward lower values. This response is too brief to be captured in the proxies, preventing us to make direct comparison. We have expanded the discussion of these results and their alignment with the geological record in the revised text (L171-174).

Figure 1: What is the C isotope gradient doing- ... does not seem to mimic at all what is thought in the d13C proxies where the gradient in the Strangelove Ocean is eliminated due to a break down in the biotic pump.
Response: We believe this discrepancy comes from comparing short-term model outputs with longer-term proxy records. To address this, we have extended the model run to 5 kyr post-impact (L170 in Supp. Info.). This extended run reproduces a carbon isotopic record that is consistent with observations, specifically, showing the collapse of the biological pump and flattening of the vertical d13C gradient (extended Data Fig. 8).

Line 112: OK so there is differential extinction between the foraminifera, zooplankton and

photosynthesisers but these statements really beg the question as to why? What is the model doing to realise these differential outcomes?
Response: These were explained in the later place of the original manuscript. But for clarity, we now move these discussions in a dedicated section of the revised manuscript (L216, section Drivers of selective extinction).

Line 122: So if there was a post extinction recovery bloom, is there any evidence for such a thing in the geological record?
Response: Multiple studies (e.g., Bralower et al. 2020 EPSL) support a post-extinction plankton bloom, which we cited in the original manuscript. For instance, our results are consistent with post-extinction biomarker data (Sepulveda et al., 2009, Science) and demonstrate the dominance of non-fossilizing plankton groups. To reduce such confusion, we discuss these comparisons earlier now in the revised manuscript (L158-160).

Line 129 paragraph: Here "the model" seems to be able to do lots of things but yet again it feels like it is treated in the write up as a black box. There is no mechanistic interpretation in a direct way derived from the model. It just does lots of stuff.
Response: We appreciate the reviewer's concern and agree again that the mechanistic interpretations needed to be clearer. We have added a dedicated section "Drivers of selective extinction".

Line 135: Why is there this differential extinction rate between the high latitude and low latitude populations?
Response: We discuss this in L216 and Fig. 4. A summary of the explanation is also provided above.

Line 118: OK so there is a carbon excursion but the model does not simulate the collapse in the 13C gradient at all- it reverses it and makes it bigger than before?
Response: Our model does reproduce the collapse in 13C gradient (see Fig. 1). We have clarified the plot by adding the title of Fig. 1i.

How could adaptation alter the results. The phytoplankton functional types are rather fixed in traits but even on a 30 year timescale there is the potential for adaptation and selection? How would that alter the results?
Response: Please see discussion above.

Line 154/155 etc. The model produces these differential impacts with no differential impact of Co2 across the latitudes. Again- why is this so? Surely this is just dependent on how your parameterise the model according to these drivers?
Response: See comments above about the mechanisms of differential extinction. In addition, as stated above for Reviewer #1, our model does not simulate the CO2 impact of ocean acidification and our results suggest that acidification was not the primary extinction mechanism, as able to reproduce most observed fossil record patterns without it.

Line 211: How is the model simulating this differential light loss across the latitudes?
Response: The model only changes the solar constant, and naturally more light loss happens in the low latitudes due to the Solar zenith angle. Our implementation assumes a globally uniform distribution of ejecta materials, based on the reference study (Senel et al. 2023), which suggests rapid global dispersal of the impact within 1 day.

Methods: Again there needs to be much more detail on the functional types and how the traits are prescribed. Selective extinction is a major part of the paper and it is not clear from what is written here how that extinction must work
Response: We have expanded the Method providing more detailed description of the functional types, traits and extinction mechanism (L322-382, and Supplementary Information).

Line 215/216: The hypothesis about preadaptation to low light at the high latitude allowing for preferential survival rates, seems to go against some of the observed rapidity of adaptation typically within the phytoplankton communities given their generation time and typical mutation rates. How would the results of the model be different should it allow for adaptation within the lineages? How would adaptation alter the results of the model?
(e.g. https://aslopubs.onlinelibrary.wiley.com/doi/10.4319/lo.1998.43.4.0679 and many other papers by Geider)
Further, Surely deeper dwellers would also have preferentially survived potentially even in the lower latitudes as the event would have removed the competition from the surface waters. Low light adaptation is quite a plastic response in phytoplankton I believe.
Response: The reviewer refers to the Geider formulation, which is already incorporated in our model to simulate photoacclimation (L391; L41 in Supp. Info.). As suggested by the reviewer, this allows plankton to adjust their Chlorophyll:carbon ratio in response to light availability, giving a selective advantage to plankton living in high-latitude, low light environments following the K-Pg impact. However, as mentioned above, we acknowledge that our model does not simulate evolutionary adaptation, which involves changes in biological traits (e.g., initial slopes of photosynthesis-irradiance curve). While adaptation could influence survivorship over long timescales, we argue that it also depends on the population size that we simulate (L369). Regarding vertical structure, the low-latitude deep dwellers did not show preferential survival across the boundary (see the comments of reviewer #1). We now clarify these points in the revised manuscript (say where specifically).

Line 229: Some of the thinking in the literature about enhanced mixotrophy, as is invoked in the aftermath of the KPg extinction, is a greater ability to transfer biomass to higher trophic levels (https://royalsocietypublishing.org/doi/full/10.1098/rspb.2016.1755). These statements seem to be in contradiction of this thinking.
Response: Our model uses a similar representation of mixotrophy as Knoll and Follows (2017). The key difference is that their work focused on the long-term evolution rise of mixotrophy (e.g., dinoflagellates) in the Mesozoic, whereas our study focuses on short-term extinction selectivity during the K-Pg event. In our simulations, mixotrophy survival mainly comes from their metabolic flexibility under extreme low-light conditions, rather than from enhanced transfer efficiency to higher trophic levels.

Referee #3
...However, being able to model this and the way the authors build their study on the best selection of results of recent studies on the end-Cretaceous mass extinction is great success and very well done.
Response: We thank the reviewer for their encouragement, and for recognizing the clear novelty of our study.

MAJOR comment (l. 123): you mention Brugger et al. 2021. They included additional nutrients coming from the impactor and show this is very relevant. How can you justify to not include this component? I suggest to add this ....or to find a good way to discuss why it works without doing so and discuss the expected changes
In addition, compared to Brugger et al. 2021 there is no overshoot of productivity - I can imagine that your model has a better representation of the involved processes, but it would be important to explain why you don't have this
Response: We now explicitly include the additional iron and phosphorus from projectile dust as in Brugger et al. (2021). To do this, we incorporated dust deposition fields from their CLIMBER model experiments into our pre-impact model run with the iron cycle (L120-123 in SI; Extended Data Fig. 3). Then we re-gridded the CLIMBER dust evolution and calculated relevant Fe and P flux from ejecta (L117; L120-123 in SI). However, these additional nutrient inputs have only a limited impact on our model results, because nutrients were already non-limiting in our initial manuscript. The main difference is that additional iron limitation reduces plankton biomass in the Late Cretaceous but does not alter extinction patterns. Importantly, our model does not show the productivity overshoot reported by Brugger et al. (2021). We attribute this difference to a key structural feature of our

approaches: our model explicitly includes plankton extinction, which Brugger et al. do not have. We now clarify this point in the revised manuscript (L164-166; Extended Data Fig. 7).

line 14 and 32: there has been a lot of research about the contribution of volcanism and/or asteroid impact to the end-Cretaceous extinction.... I suggest to rephrase these sentences and add that this is still debated or that recent studies suggest a combination of both with the impact being an essential driver (cite for example Schoene et al. 2019, Hull et al. 2020)
Response: We now acknowledge the potential role of the Deccan Trap volcanism, citing Schoene et al. (2019) and Hull et al. (2020) (L33-35).

line 6565-67: I suggest to mention the name of the trait-based model here and not only in the Methods section. I also suggest to make here already clear that the plankton ecotypes have been adapted for representing the end-Cretaceous extinction
Response: We now mention the name of model (EcoGENIE) throughout the main text. We also add "Late Cretaceous plankton" in L86 as the reviewer's suggestion.

l.69: I don't really understand this mechanisms and how this is implemented in the model and I am also not sure where to find the information the Method section. Can you either formulate this clearer here or refer more in detail where to find it in the Method section or point it out more clearly in the Method section?
Response: We added a new section in the Method (L365) to further clarify this as per the reviewer's suggestion.

l.75: I suggest to add the CO2 concentration used for the Late Cretaceous climate state as it gives together with the modeled temperature a good idea about the model
Response: The CO_2 concentration is now added (L91).

l.77: temperatures seem to match the proxy data quite well, but I guess it is high compared to other modeling studies (Niezgodzki et al. 2017, Berugger et al. 2021) - it seems like the model is doing a good job in reproducing the latitudinal temperature gradient which certainly was a problem of the mentioned modeling studies with the lower mean temperatures (as discussed in the SI of Brugger et al. 2021). Perhaps mention this as another achievement?
Response: Thanks for pointing this out. We have now added a line to highlight this strength of our model for readers less familiar with the broader context of previous model limitations (L97-99).

l.88: can you add briefly which substances are included in Senel et al. to get the solar reduction?
Response: Yes. We now specify that the Senel's model includes sulphur, soot and silicate dust (L115-117).

l. 97: for which post impact year are the numbers given for the mixed-layer depth? Would be interesting to compare with Brugger et al. 2017, they have the deepest mixed layer in the mid latitudes
Response: The number corresponds to the second year post-impact, which we have now clarified in the main text (L125). This impact is very similar to Brugger et al. (2017), who also report the deepest MLD around ~60° N/S during the same timeline (see Fig below, both showing the 2nd year after the KPg). This consistency is added in L126.

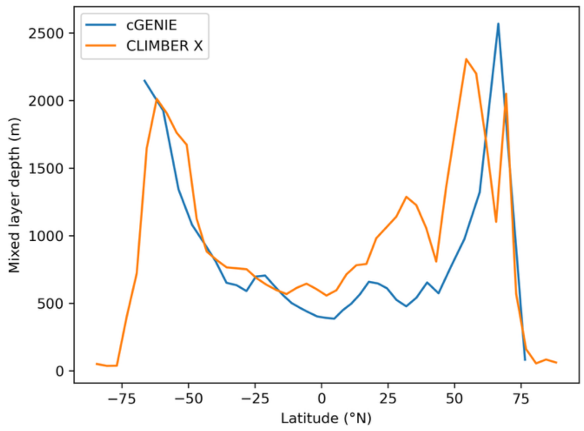

Figure 1: could you shorten the pre-impact time (I guess 5 years is enough, actually only delta13C is changing during the pre-impact phase) in order to make the most relevant years after the impact better visible?
Response: We now show 10 years of the pre-impact time in Fig. 1 so that the post-impact dynamics are more visible.

l.112 and Figure 2c: what are the surviving groups the percentage refers to? unclear to me in the text and the figure caption. (In the figure heading of 2c is a typo)
Response: Thank you for pointing this out. We have removed the reference to the "percentage surviving groups" as not needed and corrected the typo in Fig. 2c heading.

l. 125/126: I don't fully understand the remark in the brackets - can you make this clearer, perhaps just by formulating a complete sentence?
Response: We have revised this statement for clarity (L157-158).

l.141: why is this highly accurate? I find this an inappropriate term for a result of a modeling study with a very well-suited, but still very coarse and simplified model. In addition, I what does "reconstruction" refer to? Is it used here for the modeled isotope excursion? I suggest to use reconstruction in the context of isotopes in paleo studies only when it is reconstructed from proxy data, otherwise I find it confusing. Also, I don't see so much similarity to the isotope excursion in reference 36 which you mention here - can you explain what you mean or discuss the comparison of this reconstructed excursion with your modeled?
Response: We have revised the text to remove the terms "reconstruction" and "highly accurate" to avoid confusion, as suggested. We have also improved the description of our extended model experiments with carbon isotopes from Hull et al. (2020) (L167-169).

l.156: delete primarily?
Response: Done

l.167: why does temperature has an influence for high latitudes? Is it because of a limit in temperature under which it gets more difficult to survive?
Response: Both temperature and light are main limiting factors for polar plankton groups in our model. Besides, we do not impose an artificially temperature threshold; instead, these limitations emerge dynamically from the physiological constraints represented for each plankton group in the model (see comments to reviewer #2).

Figure 3: contains interesting information but I find it a not so intuitive representation I think the design of the figure would need some improvement
Response: We have revised this figure to show the extinction fraction rather than the survivor fraction, which improves its information.

l.195: make sure to call it surface ocean pH

Response: Added.

l.196: it is justified to say that acidification played a secondary role, but I would rather discuss it from the perspective that the effect was only in few regions of relevance in contrast to call a change of 0.36 a mild change - this is actually in the order of magnitude of what was found in Henehan et al. 2019 to be a "mechanism for ecological collapse". Also, could you include information on how long the pH decline lasted (I would guess it recovers quickly? - this would also be an important information considering the effect on the oceanic ecosystem)?
Response: We replaced total plankton biomass with surface pH in Fig. 1, which shows pH did not recover within the simulated time frame. Observations, albeit impacted by the coarse resolution of the fossil record in the aftermath of the KPg boundary, suggest that ocean pH took about 0.7 Myr to normalise to a pre-impact level (Henehan et al. 2019 PNAS).
We also now explicitly discuss the latitudinal variability in pH change and how it contrasts with the extinction severity (L282-284). Finally, we highlight a similar magnitude of pH change for the PETM which experienced no major extinction of planktic foraminifers and coccolithophore (L285-287).

l.197: I would not call it consistent with Brugger et al. 2021 - their region of saturation state below 1 is much larger although the effect is still limited to the high latitudes and therefore cannot explain a global extinction.
Response: we have replaced "consistent" with "similar" to better reflect the comparison with Brugger et al. (2021) (L281). See comments above about the latitudinal inconsistency.

l. 211-213: Not sure about this: is it really the absolute reduction of light which determines the extinction risk? You should have less radiation in the high latitudes, so the percentage of reduction might still be large for the high latitudes and the amount of incoming radiation small at the high latitudes as well, so you cannot argue that there is a stronger light limitation and a linked productivity loss for the low latitudes, but it would rather be because of adaptation?
Response: The extinction risk in the model depends on both the absolute light loss and the ability for photoacclimation. Although high latitudes receive less overall radiation, the absolute decrease in light at low latitudes is greater, leading to more severe biomass loss there (Fig. 4). Additionally, plankton at high latitudes tend to be better adapted or acclimated to low-light conditions (characterised by a higher Chl:C ratio), which further moderates their extinction risk. Thus, both absolute light loss and physiological adaptation contribute to the observed patterns (L219-227).

l.213/214: "Biologically, (sub)polar phytoplankton was already adapted to the low light environment" - could you add a sentence in the Methods section about how that works in the ECOGEM model?
Response: This is from a photoacclimation model (Geider 1998 L&O). We have added this into the Supplementary Information (L41).

l.232: Fig. 2e
Response: Fixed.
l.262: why only dust here? In the introduction you mention dust, sulfate aerosols and soot
Yes, we corrected them to
Response: Added.

l. 263: what is the effect of the deepened mixed layer on the light availability? You only mentioned the deepening of the mixed layer in the main part of the manuscript as a mechanism transporting nutrients to the top of the ocean and you don't say anything about its effect on light availability
Response: Thank you for your observation. Indeed, a deepened mixed layer reduces light availability, thereby diluting phytoplankton within a deeper, less illuminated layer. However, in our simulations, this effect should be minor compared to the far more pronounced reduction in PAR caused directly by the impact (Fig. 4b). For this reason, we have removed reference to mixed-layer deepening in the conclusion but kept it in the Method (L348).

l.313: the obliquity of 23.5 degree is just the present-day value as there is no knowledge about the

end-Cretaceous obliquity
Response: Added.

l.324: can you say something on why it is justified to only run it for 100 years? is it stable afterwards? Or: which variables are stable, which are not? In your figures it looked a bit like delta13C was not in equilibrium
Response: Thank you for the thoughtful comment. We have extended the run to 5,000 years to allow for the carbon isotopic record to stabilise, resulting in better agreement with geological data. Additional experiments are now described in Supp. Info. (L170).

l.333-339: a SI figure showing PAR with time would be helpful
Response: A figure has been added (Extended Data Fig. 2)

l.369-372: are the reference to Fig S6 and Fig S7 correct? I guess in l.372 there should be Figure S6 referenced and perhaps it is Fig S7 instead of Fig S6 in l. 369? For Fig S7 there is nothing said on the shown influence of solar and CO2 effect. As I don't find it self-explanatory, could you add something on this or not show it in the figure?
Response: You are right. The two figure references were indeed switched. We have corrected these and remade the figure (Extended fig. 7). We have also improved this paragraph, which is now in the Supp. Info (L44).

Referee #4:
Abstract: First read of the abstract. Very cool sounding. Novel. Synergistic.
...some of the interpretations feel a bit too neatly packaged, and **I'd like to dig into whether the patterns in the fossil record fully support the story being told.**
Also, as with any modelling study, **the details of the model matter (I ask for clarifications**), ... the way certain assumptions are set up—especially around the role of light/ darkness (and supposed prior ecological predispositions), **the real relevance of body size when it comes to already tiny organisms**, as well the **marine plankton fossil evidence** used to support some of the conclusions. Otherwise, the paper is very well written, the figures professional and clear. If the authors can come back with clarifications and satisfactorily answers to my questions this is potentially of Nature quality.
Response: We thank the reviewer's appreciation for the novelty of our study, and the recognition of the synergistic nature of our approach.

- **Model details**: We have expanded the model's description as requested (see summary above and response to reviewer#2), including how the processes related to photosynthesis are set up (L36 in SI).

- **Body size**: The reviewer is correct that body size scaling is an important aspect for extinction in the model. Extinction in the model is defined by the requirement that population must remain above one single individual, a criterion that applies across all size classes, even the tiniest plankton. We have expanded the text to clarify this point (L371-376). That aside, the allometric scaling holds even for tiny body mass as low as 10^{-10}g (Hatton et al. 2019 PNAS).

- **Fossil records**: We appreciate the reviewer's concern about aligning our model results with observational evidence. In this revision, we have expanded the model comparison with fossil record of various groups, including the prevalence of picoplankton and the extinction of foram (L146-149). Besides, in response to the other reviewers, we have strengthened the validation of our modelling approach by generating timeseries of key biogeochemical properties, such as carbon isotope, oxygen, and alkalinity to directly compare (where relevant) with available data from the fossil record.

That said, we emphasize that the goal and novelty of this work are to simulate the entire plankton community (128 plankton groups), instead of focusing on the fossilised group like foraminifera (L86-89). Moreover, the model simulates functional (not taxonomic) diversity, whereas fossil datasets are largely taxonomic and often spatially limited across the K-Pg. These mismatches prevent us against making a quantitative comparison with fossil data at specific locations. However, we believe the model still offer valuable perspective of diversity dynamics and mechanistic insights into causes of the selective K-Pg extinction.

Minor comments

• This title suggests your conclusions can be extended to all organisms? Do you think this is the case? This study focuses on marine ecosystems. I think it would be better if the title also sticks to that?
Response: We agree and have revised the title to explicitly refer to marine extinctions..

• Lines 39-41 Are we talking about all these groups here, i.e. do 'small opportunistic' versions of all of these groups exist and is a small reptile the same as small foraminifera (which are already tiny).
Response: We have rewrite this sentence to avoid such confusion (L45-49).

• Lines 42-44: I notice there are no references for the extinction response of planktonic foraminifera at high latitudes. Plus, to my knowledge, ammonites, another calcareous group, suffered total extinction both at high and low latitudes. Please address this since these groups have the best fossil records, the vertebrate fossil record is incomplete and there are no high latitude mineralizable corals.
Response: We now cite such observation for better-studied foraminifera (Huber, 1996) and replace our earlier citations for sharks and rays (L44) with one which deals with geological data from high latitude molluscs (including both ammonites, which as the reviewer suggest, suffer total extinction irrespective of latitude, and benthic molluscs) (Witts et al., 2016 Nat. Comm.). This study found identical rates of extinction in benthic molluscs as those seen at lower latitudes.

• ... i.e. evidence greater extinction survivorship at high latitudes among planktonic foraminifera? No? Why are the PF high latitude papers not cited? This part needs strengthening and a deeper coupling of the observational data with the results, albeit the different time scales under consideration here.
Response: We now cite Huber (1996), which focuses on high-latitude foraminiferal survivorship. A revision of the taxonomy of planktic foraminifer (Olsson et al., 1999 including Huber) which was published after the suggested paper (Huber 1996) examined differential extinction and appears to invalidate some of the earlier diversity analysis. More fundamentally, it is important to note that the fossil record relies primarily on presence/absence data to estimate diversity and richness. Our approach, however, encompasses all plankton groups, not only well-preserved groups like foraminifera. Besides, our approach examines changes in functional groups, which inherently limits direct comparisons of taxonomic diversity within foraminifers. This is beyond the scope of this paper (see earlier responses to reviewer 1). We have clarified these points in the revised text (L86-89).

• Lines 59-60: If Gibbs et al., already used this trait-based model approach then what is new here? Feels like the text is missing a step that identifies the 'gap' in the Gibbs et al. study.
Response: Please see our response to reviewer#2 above about differences and novelty compared to Gibbs et al. study.
• For easy access, can you please clarify what "traits"- you mean:
Response: We now define traits in the Method (L324).

• Lines 67-68: does the model directly include shell mineralogy and how? Please state.
Response: No, it does not include shell minerology (clarified in L361-363).

• Lines 69: .."evoke a mechanism based on the minimal biomass required to maintain individuals…" How is this done?
Response: this part is now detailed in the Methods (L371-374).

I think you need a sentence saying, 'using plankton as a tracer and emblematic of ecosystems', otherwise the introduction suggests you are promising the whole ecosystem.
Response: We agree and added a sentence clarifying the role of plankton as an indicator of ecosystem changes in the Method (L319-321).
• Lines 89-90: Please clarify why extinction resilience could not be a function of the existence of or possibility of dormancy strategies, i.e. ability to survive in some form for 2 years.
Response: We agree. While dormancy is not explicitly included in the model, we acknowledge its ecological relevance and address it in the revised manuscript (L236).

• Lines 110-111: Why are bigger plankton more sensitive to extinction? What is the meaning of size here? Please clarify with references relating size for already tiny things to energy needs.
Response: We now clarify the link between plankton size and extinction sensitivity in the revised text (L69-74) and have added size class information to Figure 2 to improve clarity. By size, we refer to the body size of individual planktonic organisms. Even within these tiny organisms, size plays a critical role in determining metabolic demands, resource acquisition and ecological resilience (Hatton, et. al. 2019 PNAS). Larger plankton generally have higher energy demands and lower population, making them more vulnerable to sustained environmental stress (L71).
The geological record shows that extinction events often disproportionately affect larger taxa (see Feng et al., 2024 Science for examples using foraminifera).

• Lines 155-156, and lines 190-192: is the preferential loss of calcareous plankton just a coincidence then? Explain.
Response: We now clarify this point in the revised text (L218-222). In our model, the preferential loss of calcareous plankton is due to starvation and not ocean acidification (L287-290).

• Lines 162-164: What do you think would happen if you extended the sensitivity experiments by repeating with different temperatures? i.e. could there be a lower temperature thresholds at which things behave differently?
• Lines 164-165: I'd like to know more about where these expectations come from, in terms of citations. Please reference. What did you expect? I have to say I am also surprised since other model studies using planktonic forams as a model systems find that temperature is critical in terms of environmental responses?
Response: We expect that lowering the temperature thresholds would have a limited effect. In our sensitivity experiments, we imposed large global mean temperature changes (~14 degrees), yet this produced little changes in extinction patterns (Fig. 3e). By contrast, varying light intensity strongly affects survivals, with geographical patterns in line with K-Pg fossil records. For this reason, we view light limitation as a more convincing primary driver than temperature (L201-207). This result is unexpected because our model incorporates a temperature's exponential effect on growth rates and well-documented correlation between temperature change and extinction risk of marine organisms. However, as mentioned above, our model is not simply considering foraminifera but all plankton groups and their interactions (see comments to Reviewer #1).

• Lines 183: This is important. **Are there any proxies to support the suggestion of the injection of light carbon into the atmosphere**. I know that on longer time scales export productivity change does not seem to be correlated with stratification or terrigenous input and was inferred to be driven by changes in phytoplankton (Lowry et al., 2021). Moreover, Henehan et al., (2019), also with a GENIE model component to look at pelagic d13C gradients explained the observed pattern by a less efficient biological pump (enhanced upper ocean remineralization), balancing with the solubility pump? How do these results connect here?
Response: Yes, recent works suggest injection of light carbon into the atmosphere during the first decade of the Danian, likely from wildfires and fossil carbon burning causing a rapid warming (MacLeod et al., 2018, Science; MacLeod et al., 2025, Global and Planetary Change). Additional evidence supports a CO_2 release from the Chicxulub impact from physical modelling (Kring, 2007 Paleogeography, Paleoclimatology, Paleoecology), and CO_2 reconstructions from plant megafossils (Beerling et al., 2002 PNAS). We have added references to this evidence in the revised text (L271).

The main difference between our study and Henehan2019 is the timescale. Henehan2019 reproduced the observed d13C/pH pattern at ~1myr after the boundary by changing ocean carbon cycle-related parameters. Our study however focuses on extinction with a timescale at 100-1000 years and includes the variation of atmosphere d13C. Therefore, the solubility effect will be buffered by photosynthesis/sedimentation burial within the 1-Myr window, despite it masking the rapid recovery of productivity as we proposed. We highlight such timescale difference in L273 and L315.

• Lines 214-215; REALLY: CAN YOU SAY THAT? High latitude plankton are only adapted to low light 6 months of the year, the rest its high light? No? EXPLORE?
Response: Yes, photoacclimation is well established by satellite and lab data (Behrenfeld et al. 2005, Global Biogeochemical Cycles), allowing phytoplankton to increase their Chl:C ratio in low light regardless of seasonal cycle.

• Lines 229-230: Note that N. pachyderma has adapted to food poor, light poor polar conditions, thus likely most resilient?
Response: Yes polar plankton are most resilient as we showed (Fig. 2). but N. pachyderma is a species evolved in Miocene.

• Lines 230-231 Do we really see such strong size effects in already tiny organisms? Is the difference between small /300 microns) and smaller 100 microns, significant? This needs some elaboration and refencering.
Response: As noted above, size has strong influence on ecosystem dynamics even for the tiniest organisms. Additionally, the model relies on biovolume (proportional to diameter^3) instead of diameter alone, to calculate metabolism rates, which leads to substantially greater metabolic rates in larger organisms. This scaling is well supported in metabolic theory and microbial ecology (e.g., Kyle Edwards et al. 2012, Limnology & Oceanography). We have expanded on this and included references in the revised manuscript (L371-376).

• Lines 238: benthic foram survival may also be linked to ability to survive low food supply: how long does a benthic foram live? Maybe it could surface for 3-4 years with minimal food?
Response: Yes, Hemleben & Kitazato (1995, Deep-Sea Research I) found benthic foraminifera can survive for up to five years without active feeding, relying on organic matter within sediment. This ability might have played a role in their survival across the K-Pg boundary, which may correspond to the community turnover towards infaunal taxa during the event.

• Does Genie produce a sensible representation of modern ocean circulation, including.... **where is deep water** produced by Genie in the modern, and where is it produced in the late Cretaceous? – (missing mixed layer depth plots for the late Cretaceous, also modern would be interesting).
-Genie is very simple compared with fully coupled ocean models, but would of these full complexity models also find this dramatic collapse of stratification? How plausible is it?
Response: We have added multiple new figures to the Supplementary material (L231) showing modern and Maastrichtian mixed layer depth, deep-water formation and global meridional overturning circulation. While cGENIE is a simplified Earth system model, our model outputs compare well with observational estimates of the modern ocean and more complex models for the Maastrichtian (Fig. S4). Specifically, our finding of a collapse in ocean stratification under post-impact conditions aligns with simulations from the CLIMBER model (Brugger et al., 2021), which includes the MOM2 ocean component based on the full primitive equations (Fig. S5). This consistency supports the plausibility of the stratification collapse we find. We extended the text to highlight the fidelity of our model and acknowledge its limitations, including its representation of the Arctic, which is poorly represented in the model grid.

Figure 2d: Diversity of various plankton functional groups before and after the K-Pg impact. This figure shows that, dramatically the planktonic forams went 100% extinct whereas other plankton groups did not. Why? This is not fully dealt with in the text.

2e. It would be good to add the micro, nano and pico size classes, which are the categories used in the text, to the axis of panel e) for clarity.
Response: This is likely due to their large body size and higher metabolic demand for calcification. We now clarify this point in the main text (L220-222). We have also updated Fig. 2e adding lines to distinguish the 3 plankton size categories to improve the interpretation of size-related extinction patterns.

MATERIALS AND METHODS

• Lines 285-289: Please explain further this model ecosystem. These are 'fantasy' components in a marine ecosystem, correct. You mention some foraminifera – are these assigned a skeleton mineralogy?
Response: Our model simulates the costs and benefits of calcification on zooplankton physiology but does not explicitly resolve the chemical production of foraminiferal shells. We now clarify this distinction in L357-359 in main text and L29 in SI.

• Lines 303-304: Same as I raise above -Does allometry apply even for tiny, tinier and tiniest? Please supply some references bringing in the energy/size theory for tiny plankton across the micro, nano and pico plankton size spectra, and perhaps use these terms in the text.
Response: Please refer to the answer to major comment.

I think the authors have largely done a good job of explaining and justifying the model parameterizations.
Responses: We thank the reviewer's encouragement!

• Lines 322-323" Can you elaborate on "(2) the difficulty of simulating the re-diversification process involving physical transport and evolution. This seems pretty fundamental to getting a realistic representation of the recovery process. How does this limitation impact the results?
Responses: We expand on this point in the revised text (L381-385). Indeed, as mentioned for reviewer #2, a main limitation in our model is its inability to simulate evolution of new traits to changing environments, preventing us to assess the potential impact of adaptive evolution on long-term recovery (L381). However, adaptation requires large population size for environmental selection, which we do simulate (L369).
• Lines 332-333: 2 years darkness, recovering after 4. How long can the different types of plankton survive without light? How much is known about this? Is this a parameter in the model? Please comment.
• Lines 355-357: " To simulate their impacts on biodiversity, we evoked an extinction mechanism based on plankton individual's biomass": What about a constraint on the ability of different types of zoo plankton to survive without food? E.g. can planktonic forams become dormant? Living constraints? If not, its worth saying this.
Responses: We do not set a parameter for how long a plankton can survive under complete darkness/starvation (L386-393), though we acknowledge the importance of such hypothesis. We now discuss them as unexamined trait in L235-238.

• Lines 371. There is something missing in this sentence.
Responses: This paragraph has been rewritten and hence this point has been addressed.

Responses to reviewers

Overview

We again thank our reviewers for their through and constructive comments. We have carefully revised our manuscript accordingly, as in the point-by-point responses to each comment.

Referee #1 (Remarks to the Author):

Foraminifera are indeed a small fraction of the plankton considered here (and of course in the actual ocean) but they are representative of the zooplankton as a whole, which otherwise don't have a great fossil record. Additionally, we know from observations of modern plankton that depth habitat (as a physical niche, not a proxy for light or nutrient availability) is important across all groups... So, the physical destruction of depth stratified niches would be expected to cause the extinction of taxa adapted to those niches...Also, destratification can explain the selective extinction of calcareous taxa the same way darkness can: high latitude assemblages, which tend to be dominated by siliceous taxa, are better adapted to long periods of darkness and live in an environment that is weakly or un-stratified, and so they experienced lower rates of extinction. (an aside: I am not sure how body size could explain the selection against calcareous plankton and it may be interesting to briefly address this, if you have an idea.)

My point here is that the effects of changes in mixed layer depth on nutrients, light, and temperature are certainly well-represented in your model, but the accompanying effects of the destruction of physical habitat space is not. This is fine! I think your model does a great job of illuminating the key role that darkness played in the mass extinction in the oceans, and this is an important advance! I just think **this limitation needs to be acknowledged in the text**. In particular, I think this would help explain why deep-dwelling low-latitude taxa (which were also presumably adapted to low light and could have just moved up to shallower depths) did not survive, a point also raised by reviewer #2.

Response: We thank Dr. Lowery for his insightful comments, which helped us clarify an important limitation of our approach. As expressed in our response to the previous rounds of review, we fully agree that the vertical habitat of plankton is critical in building a comprehensive view of plankton diversity and extinction. Although our model explicitly simulates the indirect effects of stratification through nutrient, temperature and light change, it does not explicitly represent the direct effects of stratification on distinct depth habitats, which many plankton occupy as dynamic preferential niches. To clearly acknowledge this limitation, we now explicitly mention this in our Method section in L373-376 and in main text (L131-132).

New Comments

Line 34-35 – I'm sorry to contradict what another reviewer said but Hull et al. (2020) isn't an appropriate reference for the idea that Deccan volcanism was a driver. They investigated the role of volcanic outgassing and found major outgassing began and ended *before* the extinction and concluded that the extinction was caused by the Chicxulub impact, although post-extinction evolution may have been influenced by Deccan volcanism in the Danian. I would cite this in the next sentence ("However, overwhelming evidence supports…") instead. (I do like how you've framed these two sentences, though).

Response: We thank the reviewer for this clarification and have moved the Hull et al. (2020) citation to the following sentence as suggested.

Line 39-40 – vaporization of anhydrite put the sulphur in the atmosphere but it was not the source of the carbon (note that anhydrite doesn't have any carbon in it). The carbon instead came from vaporization of carbonates that made up the bulk of the target rock.

Response: We agree and have revised the text clarifying this.

Figure 1 – in part (f) it would be good to extend the y-axis to zero, to make it clearer that some functional types survived, and provide an easy visual of the proportion that survived. As it is a quick

glance makes it look like they went to zero.
Response: We extended y axis of Fig. 1f to 0 as suggested.

Line 145 – you might note that the modelled result overestimates the extinction in planktic foraminifera, which did have a few survivor species. (it was 95% extinction, not 100%, which of course is not that much but also is the difference between the survival of the group and its disappearance).
Response: we have added clarifications in L154-157. In particular, we now state that the model might slightly overestimate the extinction of planktic foraminifera and nannoplankton. This discrepancy likely comes from that survival of nanoplankton is associated with coastal areas which are not well characterized in the model (Bown et al. 2005, detailed in Supplementary Information Line 265) and that planktic foraminifers might have been reseeded from benthic ancestors (Morard et al 2022, L157), a process not included in the model.

Line 195 – I assume that the role of CO2 referred to here is in driving pH change, and I want to reiterate that CO2 was not the main driver of acidification at the boundary, but rather the rapid input of sulfuric acid and nitric acid via acid rain in the years corresponding to the impact winter (e.g., Kring, 2007 Palaeo3). I think this is important context for this discussion
Response: We clarify this and address the reviewer point in the text in L206-208.

Line 221-222 – ok but what about calcareous nannoplankton? Nobody would accuse them of being big, but they suffer a much more severe extinction compared to relatively larger diatoms.
Response: We added an explanation for nanoplankton (and phytoplankton) in L238-240.

Line 346-347 – I know plankton diversity decreases with depth but I am not sure that that means "most" of plankton diversity is found in the mixed layer. Can you add a citation here?
Response: Thanks for spotting this. We have rewritten this for better accuracy (L365-367): *This simplified strategy improves the computational efficiency and reflects the nature that most plankton abundance[64] and diversity (particularly phytoplankton diversity[65]) are found in the upper ocean.*

We highlight phytoplankton because the diversity of prokaryotes is reversed and increasing with depth (Junger et al. 2023, Science Advance), but these prokaryotes are mostly bacteria.

Line 386-393 – I think this paragraph is an important and succinct summary of the model and its limitations and it would be better to put it in the main text rather than the methods, if space allows. Maybe after the paragraph that begins at Line 75.
Response: We agree this limitation would be better addressed in the main text. However, due to space constraints and reference limits, we have retained it in the Method section for the time being. We will ask the editor for permission to go slightly over the word limit to accommodate moving it into the main text.

Referee #3 (Remarks to the Author):

Thanks for revising your manuscript so thoroughly, for answering so many of my questions and incorporating a lot of my feedback. An important improvement throughout the manuscript is the way the model is introduced, named and explained. It is very understandable and these parts read much better now.

My two major comments have been addressed:
1.the nutrients from the impactor have been implemented and the results are discussed. Please make sure to correct the model name to CLIMBER-3+C and not CLIMBER-X which includes MOM3 and not MOM2 (in Figure S5 and in caption of this figure).
2. it is clear now why there is no overshoot and it seems very plausible.
Responses: We have corrected the CLIMBER model version in Fig. S5 caption.

l. 97-98: this is based on my comment on the good performance of the model in reproducing the smaller meridional temperature gradient. I find the wording misleading, as it does not explicitly say that EcoGENIE succeeds in simulating a smaller temperature gradient in contrast to other models. I suggest to rephrase, for example: In contrast to other models (28, 37), EcoGENIE succeeds in simulating a reduced equator-to-pole temperature gradient, in agreement with temperatures from proxy data. Hence, it provides a realistic climatic environment to test extinction selectivity.
l.126: it is not an ocean model and I would also not call it complex, but rather „… results from the more complex model used in Brugger…"
Responses: Both are rephrased as suggested.

l. 166 and Extended Data Figure 7: can you name the default run in a way it is clear what is included or at least describe it in the figure caption? And at least here, I find only Extended Data Fig.7 a of relevance
Responses: We add proper description of sensitivity runs in the caption of Extended Data Figure 7 now and a sentence in L175-178.

l.169: what does „high resolution" mean here?
Responses: We meant high temporal resolution in Hull et al. (2020)'s d13C data. To avoid the confusion, we removed 'high resolution' here.

Extended Data Fig.8: 1. do you compare here to the global mean? Make sure to at least mention as you compare regional data to a global mean signal (which might be acceptable for the ocean)
2. perhaps include ODP Site before the numbers?
3. I don't fully understand the caption of the figure and this is also not clear to me from the text: what are you doing with the isotope signature of the additional C - do you adjust it or not? I thought yes (and only find it justified to look at a figure like this in that case), but then I do not know what you want to say here with „do not subjectively tune"
Responses:
1. We added "global annual mean" in the caption of Extended Data Fig.8.
2. The ODP/IODP prefix is added.
3. We indeed have changed the isotopic signature of additional C. But here we mean the other uncertain parameter, the amount of emitted carbon in the model. To match the d13C data higher carbon emissions appear to be needed. We did not focus on this aspect as (1) our carbon emission is following the CO2 proxy of Henehan et al. (2019); (2) the Brugger et al. (2021) study has done sensitivity experiment to reflect this. It is an interesting observation though which should be followed up in the future to fully understand the perturbation to the carbon cycle across the boundary. We have rephrased the caption of this figure to avoid confusion.

l.188: do you mean with this sentence that you do not test the sensitivity to increased nutrients by a separate experiment? I guess you included the additional nutrients as in your combined case? or not? I

guess this just needs some rephrasing to clarify
Responses: We clarify that we have included the increased nutrient flux in the main combined experiment, but did not conduct a sensitivity experiment. We changed it to “we do not assess nutrient impacts in the sensitivity experiment”.

l.194: delete „Although“?
Responses: Done.

l.205: what is the reason for the extinction in the high latitudes when using pre-impact PAR? are the temperatures too low?
Responses: Yes, temperature limitation causes some extinction and we have added this in Line 219.

l. 219: do you really mean Fig 2d? I don’t see what this refers to?
Responses: Fig. 2d shows the different extinction ratio among different functional groups.

l.267-270: this sounds like it was a new insight, but this was already discussed in Tyrrell et al. 2015 and Brugger et al. 2021
Responses: We agree. Several previous studies have provided similar arguments (Alegret et al. 2022 for example had good discussion on 13C proxy). Therefore, we have revised this paragraph (L285-296) to reflect earlier works.
Alegret, Laia, Gabriela J. Arreguín-Rodríguez, and Ellen Thomas. "Oceanic productivity after the Cretaceous/Paleogene impact: Where do we stand? The view from the deep." (2022).

Code and data availability: the link to the specific configuration of the code (which would be the most relevant one) did not work for me. In addition, I find it crucially to find the model output used and discussed in the manuscript somewhere. There was no information on that.
Responses: We corrected the hyperlink, apologize for the problem, and uploaded all the model results to Zenodo (https://doi.org/10.5281/zenodo.17742291). We also provide more detailed instruction to compile/run the model in modern HPC environments (https://github.com/ruiying-ocean/install.cgenie).

Referee #4 (Remarks to the Author):

This revised version represents a substantial improvement. I like the new title. The abstract is much improved, better highlighting the trait-based approach, and more reflective of the study ("entire marine plankton community" works well). The incorporation of new sensitivity experiments, and the clearer linkage between extinction dynamics and body size, are also improvements. In general I find that the theoretical framing now much improved, and most reviewer concerns seem to have been addressed.

However, several issues remain that must be clarified or strengthened before the paper is ready for publication, especially regarding the **time scales of the model** and **alignment with fossil data**. Part or this should be that the paper concludes that the model is a powerful hypothesis generator, but its predictions require more robust testing against available fossil evidence before the conclusions can be considered fully substantiated (see below).
Response: We thank referee#4 for their encouraging and constructive assessment of the revised manuscript. We have further improved the discussion of model timestep and model-data comparison, which is now detailed in the Supplementary Information (Line 265, new section "K-Pg model-data comparisons"). While we agree that more model testing against additional fossil constraints would improve our analysis, we argue that the model presents sufficient agreements in the perspective of functional diversity to robustly support our main conclusions. We address these points in detail in the responses below.

1. **Clarify model time scales and realism**
I notice that the manuscript avoids any central statement or summary about the time scale over which the model operates. ..Linked to this, I might have missed it but what is the time stepping regime in the model? **Please lift this up**.

A couple of places where this would benefit:
Lines 19–20: "Here, we use a trait-based ecosystem model… across the K–Pg boundary."
Specify explicitly over what time period the simulations are run and the time steps (e.g. 100 years following impact?).
Lines 90–108: communicate how fast changes occur in the model and whether the time steps correspond meaningfully to real-world years ("years from impact" is shown in Fig. 1, but this needs explanation in the text).
Response: We now explicitly highlight the model time scales in the main text, at both positions requested by the reviewer (L20, 112) and in the Method (L337). We also clarify in Method (L337) that the chosen timestep (7.3 h) is sufficient small to capture real-world effects, including rapid processes such as trophic cascades. More technical details of other components' timestep have been added to the Supplementary Information (L125-135).

2. Relationship between model outputs and fossil evidence
The paper still treats the relationship between model predictions and the fossil record somewhat inconsistently/unsatisfactorily.
Line 44: Jiang et al. (2010) is correctly cited as showing lower extinction rates at high latitudes for nannoplankton (phytoplankton)- which supports the model results. **These data are taxonomic diversity measures. …** The rebuttal tries to side step the issue that the planktonic foram fossil record does not show lower extinction at high latitudes, as predicted by the model, by saying that "the foram fossil record only captures taxonomic diversity, whereas we model trait sensitivity". **So why were taxonomic data acceptable evidence for the nannos?** This needs some clarification.
Response: We agree that a consistent model-data comparison is needed regarding functional diversity vs. taxonomic diversity. We now emphasize in the main text that the model's extinction represents functional extinction (L152-154, L164), reflected in losses of ecological traits and ecosystem function, rather than taxonomic extinction *per se*. For example, both planktic foraminifera and calcareous nannoplankton become functionally extinct in the model, consistent with the near collapse

in abundance and $CaCO_3$ flux export across the boundary reported in the fossil record (D'Hondt, 2005, Annu. Rev. Ecol. Evol. Syst; Line 154). Similarly, other results of the selectivity of body size and dominant mixotroph after the boundary (which are functional traits) also remain robust. Therefore, the model still generates most key features of plankton extension patterns observed in the fossil record after the K-Pg boundary.

Planktonic foraminifera fossil records, however, do not show this pattern… Could this mismatch for the planktonic forams not be important on its own, rather than a flaw to step over? This is sort of there already but would benefit from focusing better.

It might even support the idea that a group level, group-specific traits (e.g. large, photosymbiotic micro-zooplankton that calcify) have their extinction selectivity modulated through compound kill mechanisms, e.g. for planktonic foraminifera starvation due to darkness and ocean acidification. So better to 'own' that there is a mismatch with planktonic foraminifera (massively used group in evolutionary studies with a celebrated fossil record), which maybe supports the idea that depending on group traits (like being a larger microzooplankton calcifier), compound kill factors should be considered.
Response: As in our last response letter, the latitudinal selectivity of planktic foraminifera is not firm. For instance, the references cited by the reviewer do not seem to contradict foram's latitudinal extinction patterns. Huber (1990) only covers the lower part of the late Maastrichtian and therefore does not include the K-Pg interval. He states that planktic foraminifera at high latitudes had lower pre-extinction diversity than at low latitudes: "*Diversity is low compared to tropical and subtropical assemblages, with a maximum within sample diversity of 16 planktonic foraminifer species and a diversity total for the Maastrichtian of 24 species.*". Stott and Kennett (1990) report similar survival levels across latitudes for this group after the K-Pg event: "*The lowermost Paleogene fossil assemblages following the mass extinction event closely resemble those of lower latitudes*". Collectively, the two references suggest that planktic foraminifera experienced less extinction at high latitudes, presenting a similar latitudinal extinction pattern to nannoplankton. However, we do recognise that Huber (1996, GSA special paper) reported reworking in these sites. Therefore, although we acknowledge that the full extinction selectively likely reflects compound effects, reworking prevents us to conclude whether the latitudinal selectivity of planktic foraminifera exists. These are expanded in the Supplementary Information. And we have carefully revised related description in the manuscript (L46-47).

Nevertheless, zooming out from discussions on planktic foraminiferal taxonomy/stratigraphy and focusing on the broader planktic ecosystem, the latitudinal selectivity for marine ecosystem extinction is robust. This is the central focus of the paper and the strength of the model.

In this respect, some reflections on the distribution of certain ecological traits in plate Cretaceous planktonic foraminifera are important. Have the authors considered the evidence of distribution of key ecological traits, such as photosymbiosis, across latitudes in the late Cretaceous in this case? E.g. do data on planktonic foram assemblages from Maud Rise ODP Sites (689/690) include photosymbiotic taxa or were there fewer in the high latitudes during the late Cretaceous? Could this be part of the story?
Response: We appreciate the reviewer's comments about the latitudinal distribution of ecological traits through this event. At present, however, direct constraints on the trait distribution of symbiosis of Cretaceous taxa is limited (particularly on the sites highlighted by the reviewer). The most relevant available studies include Houston and Huber (1998, Marine Micropaleontology), Hondt and Zachos (1998, Paleobiology) and Abramovich et al. (2003, Marine Micropaleontology). According to these studies, it appears likely that many of the high latitude taxa did not harbour symbionts. For example, D'Hondt and Zachos (1998) stated: "*Like modern photosymbiotic taxa (Hemleben et al. 1989), Pseudoguembelina species, Racemiguembelina species, and Planoglobulina acervulinoides were generally limited to tropical and subtropicalopen oceans (Nederbragt 1989; Huber 1992)"*. This inferred trait distribution generally agrees with our model outputs, which show preferential lass of symbiotic foraminifera in low (oligotrophic) latitudes (figure below and added in Supplementary Line

257-263). However, the further exploration and relation with the reviewer's hypothesis is beyond this study's scope because of the difficulty of identifying latitudinal selectivity of foraminifera as in the last response.

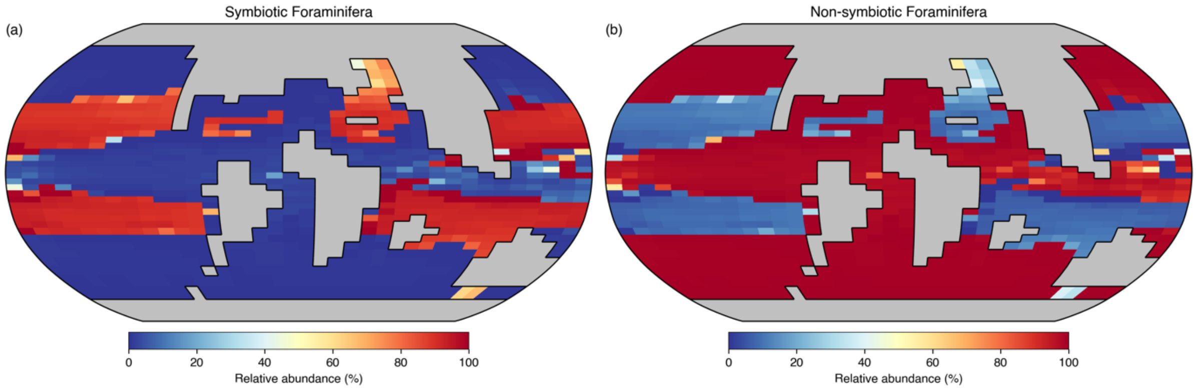

Fig. S9. The simulated Late Cretaceous symbiosis trait distribution for planktic foraminifera.

3. References and fossil context

The extinction process section (around Line 38) is missing relevant literature on post-K–Pg planktonic foram rediversification uncertainty—e.g. whether new planktonic forms evolved from benthic ancestors or surviving planktonic lineages. (Olsson et al., 1999 vs Arenillas et al., 2022; Darling et al., 2009; Morad et al., 2022).

Olsson, R.K., Hemleben, C., Berggren, W.A., Huber, B.T., 1999. Atlas of Paleocene planktonic foraminifera. Smithsonian Contributions to Paleobiology 85, 252.

Darling, K.F., Thomas, E., Kasemann, S.A., Seears, H.A., Smart, C.W., Wade, C.M., 2009. Surviving mass extinction by bridging the benthic/planktic divide. Proc Natl Acad Sci USA 106.

Morard, R., Hassenrück, C., Greco, M., Fernandez-Guerra, A., Rigaud, S., Douady, C.J., Kucera, M., 2022. Renewal of planktonic foraminifera diversity after the Cretaceous Paleogene mass extinction by benthic colonizers. Nature Communications 13, 7135.

Arenillas, I., Arz, J.A., Gilabert, V., 2022. An updated suprageneric classification of planktic foraminifera after growing evidence of multiple benthic-planktic transitions. Spanish Journal of Palaeontology 37, 1-34.

Response: We thank the reviewer for the suggestion. The Morard et al. (2024) paper is cited in Line 153-155. However, because our simulations focus on the first 100 years after the extinction and aim to detect extinction mechanisms across the entire plankton, the question of re-diversification over subsequent millions of years fall outside the scope of our study.

(other related comments aggregated to here by Authors)

Note some additional references relevant to the high latitude planktonic foraminifera records that are relevant.

-Keller, G., 1993. The Cretaceous-Tertiary boundary transition in the Antarctic Ocean and its global implications. Marine Micropaleontology 21, 1-45.

-Stott, L.D., Kennett, J.P., 1990. Antarctic Paleogene planktonic foraminifera biostratigraphy: ODP Leg 113, Sites 689 and 690., in: Barker, P.F., Kennett, J.P.e.a. (Eds.), Proc. ODP, Sci. Results. (Ocean Drilling Program), College Station, TX, pp. 549-569.

-Huber, B.T., 1990. Maastrichtian planktonic foraminifer biostratigraphy of the Maud Rise (Weddell

Sea, Antarctica): ODP Leg 113 Holes 689B and 690C, in: Barker, P.F., Kennett, J.P., al., e. (Eds.), Proceedings of the Ocean Drilling Program, Scientific Results. College Station, TX (Ocean Drilling Program), pp. 489–513.
Response: These references have been added to the new K-Pg model-data comparison in the supplementary information.

Planktonic foraminifera fossil records, however do not show this pattern. That this is the case is important given that they have such a fantastic fossil record, including indicators of ecologic traits together with taxonomic diversity, and are celebrated in many other recent high profile studies as a 'model system', including these papers below.

Larina, E., Woodhouse, A., Swain, A., Lowery, C.M., Martindale, R.C., Myers, C.E., 2025. Regional restructuring in planktic foraminifera communities through Pliocene-early Pleistocene climate variability. Nature Communications 16.

Swain, A., Woodhouse, A., Fagan, W.F., Fraass, A.J., Lowery, C.M., 2024. Biogeographic response of marine plankton to Cenozoic environmental changes. Nature 629, 616-623.

Woodhouse, A., Swain, A., Fagan, W.F., Fraass, A.J., Lowery, C.M., 2023. Late Cenozoic cooling restructured global marine plankton communities. Nature 614, 713-718.
Response: We fully agree that planktic foraminifera are an excellent group with a long track record on studying their ecology and evolution. But we have too limited reference space to cite these papers because they focus on Cenozoic diversity, whereas our study specifically focuses on extinction mechanisms at the K-Pg boundary.

4. Body size and extinction mechanisms
Generally, the stronger focusing on body size works well. The improved background theory in relation to this is an improvement.
In this respect, regards planktonic foraminifera, I suggest you refer to them as 'microzooplankton' since they are certainly not large among the zooplankton generally.

Line 220: Correct the statement—rewrite as : "planktonic foraminifera are large within the microzooplankton community"
Response: Accepted.

5. Model–data comparison and sensitivity tests
Line 186: "The strong model–data agreements allow…" — please verify this claim carefully, as the fit is not uniformly strong across groups. As far as I can see the model data-agreement involves a short written presentation of some fossil record data types (Lines 45-47):
"Notably, high-latitude nannoplankton exhibited lower extinction rates than low-latitude counterparts7, though such latitude-dependent extinction was not found in foraminifera 19 and molluscs 20."

I would say that this is not strong model agreement. The two key groups with excellent fossil records, planktonic foraminifera and molluscs we are told do not show latitude-dependent extinction patterns.
Response: See the response above to this comment and our synthesis in the Supplementary Information (Line 265). We believe that the model reproduces essential patterns observed in the fossil observations for climate, ecology and biogeochemistry across the K-Pg boundary and in the Late Cretaceous. For example, the model captures the severe functional loss of planktic foraminifera and nannoplankton, consistent with the literature, in agreement with the reviewer. There are ample threads of evidence that survivors are small or mixotrophic, similarly to the model results that highlight the ecological benefit of mixotrophy in post-K-Pg environments. Extended model runs now allow comparison with carbon isotope gradients, showing a decline of vertical carbon isotopes gradient, in

line with the observations. Molluscs are not represented in our model, as our analysis focuses on planktonic communities.

At the same time, we acknowledge the model limitations, including slight overestimation of foraminiferal extinction. Consequently, we have revised the text describing the model fit as an “overall agreement” rather than “strong agreement”, reflecting both the model successes and its limitations.

Is the type of plankton that do show a pattern also important: nannoplankton = calcareous nano phytoplankton?
Response: The record of non-calcifying nannoplankton is limited. However, evidence from the modern ocean suggests body size is a master ecological trait influencing geographical distribution with smaller plankton mostly occupying oligotrophic oceans. Therefore, as our main argument, body size is likely more important in shaping extinction and distribution patterns than calcification.

7. Conclusions section
I suggest that the time frame of extinction should feature in the conclusions. See above.

Considering my comments above, it is also important to state **that the model remains a powerful hypothesis generator**, with some fossil data support, but its predictions require more robust testing against available fossil evidence before the conclusions can be considered fully substantiated.
Response: We fully agree here and acknowledge the model’s limitation. The conclusion is revised as suggested (Lines 312 and 320).

Figure 3: Please define PAR (photosynthetically active radiation) clearly in the caption.
Response: Added.

Referee #4 - remarks on the earlier report from Referee #2
-Novelties and mechanistic insights
Ref 2 was worried about this, raising the question of novelity of the model and its results multiple times. I.... I was similarly wondering what made this study so radically new compared to others but that is articulated now.

-Its clearer now also how the model is contstructed- the 'body size' and 'organism-sensitivity to light' features (the latter being indirect through the distribution of trophic traits in the organisms), being the additional ecological parameters 'feeling' the effects of the imapact event, and allowing the new conclusions.
Response: We sincerely thank referee #4 for carefully considering referee #2's comments and recognising the improvements we made to address their concerns.

Ref 2 was concerned about and was sceptic over the issue of adaptation of organisms within the model. The authors combat this by making the argument: "However, adaptation occurs across generations and need sufficient population size for natural selection, with larger population more likely to adapt and survive (as reviewer commented). This effect is implicitly represented in our model simulating population size, which is used to determine extinction/survival (L369, L382)." So if I understand right they 'lump' potential to adapt with population size, by which I suppose they are implying that since extinction first requires a decimateion of population size, adaptation becomes difficult?

The comment about the modelled surface-deep DIC d13C gradient not looking at all like the proxy data by Ref -2 is very valid. The authors comment and that this discrepancy cames from comparing short-term model outputs with longerterm proxy records, now backed up with an extended model run that now reproduces a carbon isotopic record consistent with observations (showing collapse of the biological pump and flattening of the vertical d13C gradient) is definitely reassuring that the model is making a decent job of mimicking carbon cycling at the time.
The other concerns by ref 2 seem to have been addressed.
Response: Again, we thank referee #4 for the endorsement.

Responses to reviewers

Overview

We thank our reviewers for their appreciation in our last round of revision. We have further revised our manuscript according to the new comments with the point-by-point responses to each comment. The new changes in the manuscripts are highlighted in yellow for tracking changes.

Referee #1 (Remarks to the Author):
I am satisfied with the revisions that the authors have made and think this paper is ready for publication with a couple very minor changes listed below. I do not need to see it again.

I really like how the authors frame the ongoing uncertainty about specific mechanisms of extinction in the marine realm in the introduction, nicely setting up both the scientific motivation of this work and the use of a mechanistic ecosystem model to address these questions.

The paper does an excellent job of walking through the results of the model runs, their implications, and their limitations. It makes a compelling argument for the importance of energy demand and plankton body size in explaining the selectivity of the K/Pg extinction, and how that can be explained through darkness after the Chicxulub impact. I think it does more than simply serve as a "hypothesis generator" as another reviewer puts it (it does a pretty good job of testing hypotheses for extinction in my opinion), but importantly in light of my earlier reviews I think the authors do a good job stating the limitations of their model and not overstating the strength of the interpretations (which are very strong! just not bulletproof).

Discussions of the mechanisms of the K/Pg mass extinction in the oceans have bounced around the same questions for a few decades now about acidification and Strangelove Ocean vs. Living Ocean, etc., and I think the introduction of ecosystem modelling will reframe the conversation in helpful ways to move us all forward to new questions. This paper thus represents an important step forward in our understanding of the K/Pg mass extinction in the plankton (I will certainly be citing it a lot moving forward) and I think it absolutely deserves to be published in Nature.
Response: We thank Dr Lowery for his helpful comments throughout the reviewing process and his generous support of our model interpretation.

Line 156-157 – Here's a pedantic foram point: I don't think anyone debates that at least one planktic foraminifera (Guembelitria cretacea) survived the extinction, though there are some debates about other possible survivor species which really just get into how likely you think that some short-ranged species actually lived in the Danian or were just reworked for a little while (the number of these broadly considered survivors is actually pretty low so one could argue that the current estimate underrepresents survival). Raphael Morard's work suggests that Cenozoic planktic foraminifera had a benthic ancestor, but that does not mean that there were no survivors, just that the survivors have no modern descendants (as Morard et al. point out in their paper). So, observations probably don't overrepresent survival.
Response: This is correct. We removed this sentence which might over-interpret Morard's work.

Line 186 – "early" Danian is not a formally defined substage and so early should not be capitalized
Response: Fixed.

Referee #3 (Remarks to the Author):
I enjoyed reading the manuscript again a lot! It reads very nicely. My comments have all been adressed and I only have two comments linked to two of my comments from the last review round. Both are not critical, they do not necessariliy need to be adressed.
Response: We thank the referee for their appreciation, which helped to improve our revision. The two comments are addressed in the new manuscript and we have updated the instructions on model output.

l.169: what does „high resolution" mean here?

Responses: We meant high temporal resolution in Hull et al. (2020)'s d13C data. To avoid the confusion, we removed 'high resolution' here.

no, this is actually not removed here, it is still here. In principle I don't have a problem with calling it "high resolution", as it is high resolution compared to other records, but I would find it important to clarify here that high temporal resolution of the proxy record for KPg is still not comparable to modeled temporal resolution, as it is only possible to say the record has a resolution between yearly to 100 to 1000 years, so this will stay a limitation (which does not mean that I find it not justified to compare it anyways). You discuss this in the end of the paper, but here it sounds like the temporal resolution is the same for the record and the modeled results. As far as I know Hull et al.(2020) can also not limit the resolution to smaller intervals like yearly?
Response: The original statement was deleted but this one [new Line 169] points to similar studies (albeit also temporally of a broader duration than our model)) which use organic matter (biomarker) proxies to 'fingerprint' changes in the plankton community in the early Danian. Nevertheless, we do agree that no proxy reaches the temporal resolution of our model and it could cause confusion by saying "high resolution" proxy. We delete it at both places.

l.188: do you mean with this sentence that you do not test the sensitivity to increased nutrients by a separate experiment? I guess you included the additional nutrients as in your combined case? or not? I guess this just needs some rephrasing to clarify
Responses: We clarify that we have included the increased nutrient flux in the main combined experiment, but did not conduct a sensitivity experiment. We changed it to "we do not assess nutrient impacts in the sensitivity experiment".

ok, I understand now what you want to say with this sentence. I actually find this sentence not necessary and I wold find it clearer to just delete it, but this is up to you.
Response: We find this sentence redundant too. It is removed now as the referee's suggestion.

My only additional comments refer to the documentation of the model output - here, I see some important information still missing, see below.
If this is adressed, I recommend to accept the paper for publication.

Referee #3 (Remarks on code availability):
I did not try to run the code, but I looked at the structure of the code and the documentation in the readme. For the code this looks good to me.
For the model output, it is not clear to me which files I need to reproduce figures and results. This is not documented in a readme and there is a huge amount of files I would not know what to do with in the zip folder which I downloaded from the zenodo link. In my opinion it is necessary to provide a readme with the model output stating which output files have been used for the figures and for the calculated results. In addition, the scripts to reproduce the figures and results should also be provided (I did not find them), together with comments and a readme file.
I addition, I suggest to not upload one huge compressed file, but compress the directories, which were in there, separately and put the readme(s) not inside a compressed file (then I can find out what to download for a certain question and do not need to download all the data).
Response: Thanks for the suggestion. We compress each model run separately now and upload a more detailed readme instruction (https://doi.org/10.5281/zenodo.17742290). We also uploaded the scripts for generating the figures in https://github.com/ruiying-ocean/kpg_selectivity (added in the main text code availability), to help reproduce our figures.

Referee #4 (Remarks to the Author):

I am largely satisfied that the authors have addressed my main concerns from the previous review. In particular, the clarification of model time scales and timestepping is now clear and appropriate, which

improves the paper.
Regarding data comparison, I focus on the planktonic foraminiferal fossil record, which remains the best available dataset. While gaps remain—particularly concerning ecological trait distributions and continuous high-latitude Cretaceous records—the authors now acknowledge these limitations. A few final clarifications would strengthen the paper
Response: We thank the reviewer for their positive feedback and are glad that the revisions have addressed their main concerns. The two clarifications are done as suggested.

1. Functional type distribution (Lines 85–87): Are the 16 functional types distributed across latitudes in a realistic manner, for example relative to modern observations? This is important because traits such as photosymbiosis are not evenly distributed across latitudes in the modern or Pleistocene oceans, being largely absent at high latitudes. The authors' prior work (Ying et al., 2023, 2024) modeled these distributions in detail, but this is not explicitly referenced here. Please clarify how realistic latitudinal trait distributions are implemented in the model.
References:
• Ying, R., Monteiro, F. M., Wilson, J. D., Ödalen, M., & Schmidt, D. N. (2024). Past foraminiferal acclimatization capacity is limited during future warming. Nature, 636(8042), 385–389.
• Ying, R., Monteiro, F. M., Wilson, J. D., & Schmidt, D. N. (2023). ForamEcoGEnIE 2.0: Incorporating symbiosis and spine traits into a trait-based global planktic foraminiferal model. Geosci. Model Dev., 16(3), 813–832.
Response: Yes, the model uses the same plankton configuration as in our prior work, where the latitudinal distributions were evaluated against observations to show realistic patterns. We have updated Fig. S9 to show this. The model for example does not have symbiotic foraminifera in high latitudes, consistent with modern observations and related to your next comment. We now clarify this at Lines 381-384 and explicitly cite our previous papers.

2. Extinction selectivity at high latitudes with respect to planktonic forams: I am still unclear on how the scarce high-latitude fossil records support lower extinction selectivity during the K/Pg event. High-latitude sites show lower pre-extinction taxonomic diversity, which implies lower functional trait diversity (modern high-latitude faunas also lack photosymbiotic taxa). **Does lower starting functional diversity truly indicate reduced extinction selectivity, or could it instead reflect the predominance of generalist or opportunistic taxa?**

Although the study does not focus on planktonic foraminifera, the argument could be strengthened by considering **the ecological traits of likely survivors and pre-extinction distributions** for this group- mention in the supp infor maybe? Current evidence suggests very few true survivors globally. Guembelitria cretacea, widely interpreted as non-symbiotic, opportunistic, and stress-tolerant, is the clearest likely survivor. Early Paleocene high-latitude occurrences of Hedbergella and Heterohelix may represent survivors, but reworking cannot be excluded. Other common pre-K/Pg genera, such as Guembelitrioides, were likely not survivors and were non-symbiotic.
This suggests that high-latitude assemblages had few specialized ecological traits prior to the extinction. Keller and Pardo (2004) provide a key reference mapping these high-latitude occurrences and highlight the dominance of opportunistic Guembelitrinidae. **How does the model reconcile this with its finding of lower extinction selectivity for planktonic foraminifera**? This is where clarify how the initial trait distributions at high latitudes are represented in the model is important, and how they inform the simulated extinction patterns.
References:
• Morard, R., Hassenrück, C., Greco, M., Fernandez-Guerra, A., Rigaud, S., Douady, C. J., & Kucera, M. (2022). Renewal of planktonic foraminifera diversity after the Cretaceous–Paleogene mass extinction by benthic colonizers. Nature Communications, 13, 7135.
• Keller, G., & Pardo, A. (2004). Disaster opportunists Guembelitrinidae: Index for environmental catastrophes.Marine Micropaleontology, 53, 83–116.
• Huber, B. T. (1996). [Relevant pre-K/Pg planktonic foraminiferal taxonomy.]
• Olsson, R. K., et al. (1999). [Early Paleocene high-latitude records.]

Overall, given the limitations of the fossil record, the authors' arguments are reasonable, and the hypothesis is appropriately framed. The phrase "and potentially planktic foraminifera19" adequately acknowledges these limitations, but explicit discussion of functional trait distributions would further clarify the modeling results.
Response: We thank the reviewer's acknowledgment that our hypothesis is appropriately framed but a few clarifications are needed. Specifically, the reviewer raises an important point that high-latitude assemblages might not show true selectivity because pre-extinction diversity was low. We clarify that this does not confound our interpretation because the latitudinal gradient in extinction selectivity directly reflects selection against photosymbiotic taxa. High-latitude assemblages experienced lower selectivity precisely because they did not rely on photosymbiosis, reducing the impact of light limitation associated with the K-Pg event. Therefore, the lower selectivity at high latitudes reflects the pre-extinction absence of the symbiosis trait, rather than an artefact of sparse fossil records or low diversity per se.

We add this clarification in the supplementary material (Lines 298-302) noting that the latitudinal gradient in selectivity reflects the pre-extinction distribution of photosymbiotic taxa, citing key references, including Keller and Pardo (2004). We also explain how the initial trait distributions at high latitudes are represented in the model and how it strengthens our interpretation (Lines 263–265).

Responses to reviewers

Referee #3 (Remarks to the Author):
Thanks for addressing my comments again and especially for providing all data and scripts, a helpful readme and for saving the data in separate zip files. It is clear how to use the data now. I agree that the paper can be published.
Julia Brugger

Remarks on code availability:
as stated above, this is fine now!
Response: Thank you Dr. Brugger!

Referee #4 (Remarks to the Author):
Thanks for recognizing the these important aspects of the planktonic foram records - this is satisfactorally is acknowledged now, although its a shame that a lot of important information about the mechanistics of the model and the sensitive parameters (like distriibution of photosymbiosis) gets shuffled to the SI section.

The manuscript now states in the Abstract (line 24) and main text (line 47) that the model reproduces "reduced diversity loss in high-latitude taxa", citing Ref. 7 for observational support. However, there are issues with the nannofossil records too, and the cited study on calcareous nannoplankton (Jiang et al. 2010 K/Pg nannoplankton extinction latitudinal patterns) does not provide evidence for a high-latitude refuge effect in the way implied here.

While the study by Jiang et al reports substantial geographic variability in extinction intensity, the dominant pattern identified is hemispheric asymmetry, with the highest extinction levels in the North Atlantic, North Pacific and Tethyan oceans and lower extinction intensities in the Southern and Indian oceans. Importantly, this pattern is not interpreted as reduced diversity loss at high latitudes, but rather as a consequence of the physics of the impact event. The authors conclude that an oblique, northward-directed Chicxulub impact concentrated ejecta in the Northern Hemisphere, suppressing photosynthesis there and producing the observed extinction gradient.

Thus, the cited reference supports hemispheric differences linked to ejecta distribution, rather than preferential survival of high-latitude taxa. The manuscript should revise the text to reflect the conclusions of the cited observational work more accurately.

This hemispheric asymmetry aspect of the Jiang et al paper's conclusions will of course not be in this current model, but the details of the nannofossil observations should not be glossed over. As written, the text presents the observational evidence as supporting a high-latitude refuge signal that is not clearly demonstrated in the cited study. This must be addressed.
Response: We thank the reviewer for their detailed interest in our model, and for continuing to recognise the importance of comparing model output to available fossil data.

Jiang et al. interpret the observed geographic variability in extinction intensity as a consequence of hemispheric asymmetry driven by northward-directed ejecta from the Chicxulub impact. However, the underlying data themselves cannot distinguish between a latitudinal pattern and a hemispheric pattern, because Northern Hemisphere high-latitude sites are lacking (most sites in Jiang et al. are around 30°N). In fact, Jiang et al also acknowledge the importance of adaptation to light loss in driving survival of small high-

latitude forms, which is entirely consistent with our results. Additionally, more recent work based on data from IODP Expedition 364 to the Chicxulub crater (Collins et al., 2020) suggests that the angle of impact was steeper than previously thought (45 – 60 degrees) and from the northeast – at odds with the hypothesis of Jiang et al.

To reflect the truth of data, we added "potential for reduced diversity loss in high-latitude settings" in Line 24 and revised the Introduction (L47-51, copied below). These changes are highlighted as yellow in main text.

"Notably, high-latitude nannoplankton[7], particularly in the Southern Ocean, have exhibited lower extinction rates than low-latitude counterparts, and a similar pattern might exist for planktic foraminifera[19]. However, Northern Hemisphere high-latitude data remain limited[7] and such a latitude-dependent extinction was not found in molluscs[20]"

References

Collins, G.S., Patel, N., Davison, T.M., Rae, A.S.P., Morgan, J.V., Gulick, S.P.S. IODP-ICDP Expedition 364 Science Party & Third-Party Scientists. Nature Communications 11, 1480.

Jiang, S., Bralower, T.J., Patzkowsky, M.E., Kump, L.R., Schueth, J.D. 2010. Geographic controls on nannoplankton extinction across the Cretaceous/Paleogene boundary. Nature Geoscience 3, 280-285.
